# Global burden of disease study analysis of thyroid cancer burden across 204 countries and territories from 1990 to 2019

**DOI:** 10.3389/fonc.2024.1412243

**Published:** 2024-05-28

**Authors:** Zhili Dou, Yanyan Shi, Jinzhu Jia

**Affiliations:** ^1^ Department of Biostatistics, School of Public Health, Peking University, Beijing, China; ^2^ Research Center of Clinical Epidemiology, Peking University Third Hospital, Beijing, China; ^3^ Center for Statistical Science, Peking University, Beijing, China

**Keywords:** burden of disease, disability-adjusted life-years, prevalence, incidence, deaths

## Abstract

**Background:**

The purpose of this study is to assess the burden of thyroid cancer over the course of 30 years in 204 countries and territories.

**Methods:**

Data from the Global Burden of Disease (GBD) 2019 database was analyzed to extract information on prevalence, deaths, DALYs(disability-adjusted life-years), YLL(years of life los), YLD(years lived with disability), and their corresponding age-standardized rates at global, regional, and national levels. The primary focus of the study was to examine trends in thyroid cancer from 1990 to 2019, specifically looking at the Estimated Annual Percentage Change (EAPC) for ASPR, ASDR, and ASDR. Additionally, the study investigated the relationship between cancer burden and the Socio-Demographic Index (SDI).

**Results:**

Globally, there will be approximately 18.3 million thyroid cancer (TC) cases in 2019; China and the USA are projected to be the most significant with 310,327 and 220,711 cases (16.17 and 14.82 cases per 100,000 people, respectively).Over the period from 1990 to 2019, age-standardized prevalence rates exhibited a global rise, whereas deaths and DALYs saw a decrease(EAPC:1.63, –0.15- –0.14, respectively). Significantly, the age-standardized prevalence rate increased in 21 GBD regions, affecting 195 out of 204 countries or territories. Over the studied period, thyroid cancer cases, deaths, and DALYs were consistently higher among females than males. Furthermore, a higher Socio-demographic Index was associated with increased age-standardized prevalence rates.

## Introduction

Thyroid cancer(TC) stands as the most prevalent endocrine cancer on a global scale (1). Over recent decades, numerous research have noted a consistent upward trend (2) in thyroid cancer incidence across various countries and regions, including Canada (3), the US (4), Australia (5), Asia (6–8), South America (9), and Europe (10–13).While several regional studies have provided insights into thyroid cancer’s incidence and mortality rates (14, 15), research examining its correlation with factors like country, gender, age, and sociodemographic index (SDI) remains limited. A comprehensive analysis of thyroid cancer worldwide, considering a range of factors, could significantly enhance healthcare planning and the allocation of resources. The Global Health Data Exchange, a publicly accessible platform covering numerous human diseases and injuries across many countries and territories, provides an invaluable opportunity to explore thyroid cancer’s distribution and evolving patterns (16). Utilizing age-standardized rates for analysis can assist policymakers in understanding the burden of thyroid cancer, tracking treatment progress, optimizing resource allocation, and developing applicable regulations. This study seeks to explore the ongoing trends and changes in the incidence, mortality rates, and disability-adjusted life-years (DALYs) related to thyroid cancer.

## Methods

### Collection of data

The methodology employed in the GBD 2019 study has been thoroughly explained in previous publications (17–19). In essence, data for the GBD Study were gathered from various sources such as censuses, surveys of households, and others (18). We utilized a Bayesian meta-regression modeling tool known as Dis Mod-MR 2.1 to analyze and estimate the burden across different conditions in 204 countries and territories from January 1,1990, to December 31, 2019 (18, 20). This model incorporated information available for each disease and applied corrective measures for known biases to calculate country-specific prevalence and burden estimates, as detailed in previous studies (17). Uncertainty intervals (UIs) were determined using ordered draw values of the posterior distribution (18). We retrieved annual crude and age-standardized estimates of TC burden from the GBD database covering the period from 1990 to 2019, along with their corresponding 95% uncertainty intervals (UIs), using the Global Health Data Exchange query tool. The variables obtained encompassed prevalent cases, deaths, disability-adjusted life-years (DALYs), years of life lost (YLL), years lived with disability (YLD), and their age-standardized rates (ASRs) at the global, regional, and national levels. The prevalence rate per 100,000 individuals was computed by dividing the total cases (including new and previously diagnosed cases) by the population size.

These data were further stratified by age groups, calendar years, regions, and countries/territories. Geographically, the world was categorized into 21 regions, and the 204 countries/territories were classified into five SDI categories (high, high-middle, middle, low-middle, and low), with higher SDI denoting more developed countries (18).

### Statistical analysis

Estimated average percentage changes (EAPCs) were utilized to assess trends in ASRs of prevalence, deaths, and DALYs over a specified timeframe. The assumption of linearity between the natural logarithm of ASRs and time was adopted, following the equation y=α+βχ+ε where y=ln (rate), x=calendar year and *ε*=errorterm (21). Based on this formula, *β* represents the positive or negative ASR trends. The EAPC was calculated as 100 × (exp (*β*) – 1), with its 95% confidence interval (CI) derived from this model (21). An ASR was considered to exhibit an increasing trend if the 95% CI of the EAPC estimation was >0, a decreasing trend if the 95% CI was <0, and stability if the 95% CI included 0. Statistical analyses and visualizations were conducted using the R statistical software program (version 4.1.0), with a significance level of p < 0.05 (21). This study followed the guidelines outlined in the Guidelines for Accurate and Transparent Health Estimates Reporting for cross-sectional studies (22).

## Results

### Thyroid cancer worldwide

**Figure 1:**
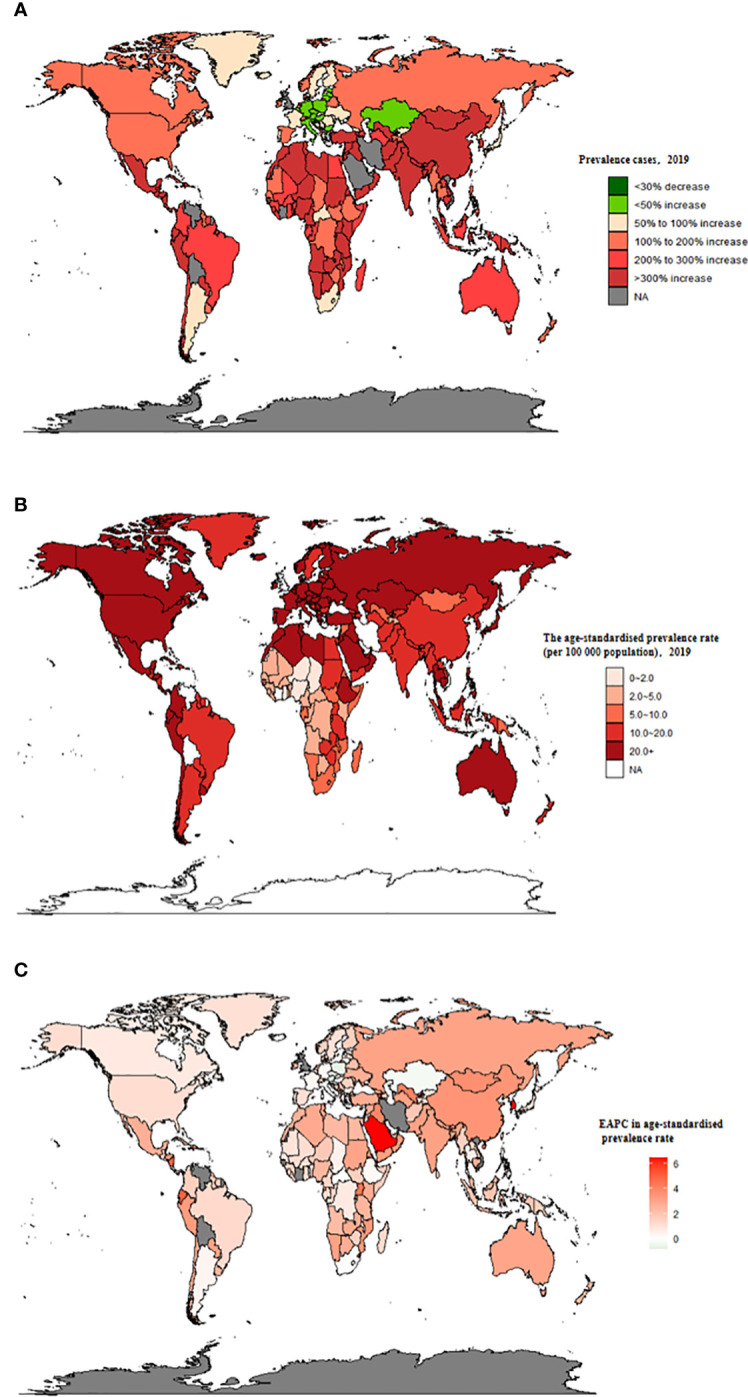
The global prevalence burden of TC in 204 countries and territories. **(A)** The absolute number of TC prevalent cases in 2019. **(B)** The age-standardised prevalence rate (per 100 000 population) of TC in 2019. **(C)** The EAPC of age-standardised prevalence rate for TC between 1990 and 2019. EAPC, estimated annual percentage change; TC, thyroid cancer.

Globally, thyroid cancer saw 87,583 incident cases (95% uncertainty interval [UI]: 82,236–92,717) and 22,966 deaths (95%UI: 21,554–25,228) in 1990, increasing to 233,847 incident cases (95% UI: 211,637–252,807) and 45,576 deaths (95%UI: 41,290–48,775) in 2019 (**Supplementary Table S4**). This translated to a total of 1,231,841 disability-adjusted life-years (DALYs) (95% UI: 1,113,585–1,327,064) in 2019. Over the 1990–2019 period, there were notable increases in incident cases (167%), deaths (98%), and DALYs (85%) attributed to TC. In addition, the age-standardized rates (**Figure 1B**; **Supplementary Figures S3, S10**) and the changing trends differed among various countries (**Figure 1C**; **Supplementary Figures S11, S12**). The age-standardized death rate (ASDR) (EAPC: -0.15%; 95%CI: -0.19 - -0.12) and age-standardized DALY rate (EAPC: -0.14%; 95%CI: -0.18 –0.1) demonstrated a declining trend (**Table 1**; **Supplementary Table S5**). Furthermore, ASIR trends differed by gender, with males experiencing an increasing trend (EAPC: 1.89%; 95% CI: 1.77–2.02) while females showed a slight increase (EAPC: 0.98%; 95% CI: 0.85–1.12). In terms of ASDR and age-standardized DALY rate, males displayed an increasing trend (EAPC of ASDR: 0.69; 95% CI: 0.61–0.76; EAPC of DALY rate: 0.61; 95% CI: 0.53–0.68), whereas females exhibited a decreasing trend (EAPC of ASDR: -0.59%; 95% CI: -0.63 - -0.56; EAPC of DALY rate: -0.57; 95% CI: -0.62 –0.52).

**Table 1:** Age-standardised prevalence, death and DALY rates for inflammatory bower disease (TC) in 1990 and 2019 and their temporal trends from 1990 to 2019.

	Age-standardised prevalence rate per 100000 population			Age-standardised death rate per 100000 population			Age-standardised DALY rate per 100000 population		
Characteristics	1990no(95%UI)	2019no(95%UI)	EAPC no(95%UI)	1990no(95%UI)	2019no(95%UI)	EAPC no(95%UI)	1990no(95%UI)	2019no(95%UI)	EAPC no(95%UI)
Global	14.06 (13.19–14.74)	22.02 (19.91–23.86)	1.63% (1.48–1.78)	0.6 (0.56–0.66)	0.57 (0.51–0.61)	-0.15% (-0.19–0.11)	15.55 (14.4–17.02)	14.98 (13.55–16.14)	-0.14% (-0.18–0.1)
Sex									
Male	7.22 (6.67–7.68)	13.7 (12.25–14.97)	2.42% (2.26–2.57)	0.43 (0.4–0.48)	0.51 (0.46–0.55)	0.69% (0.61–0.76)	11.21 (10.21–12.64)	12.93 (11.7–14.02)	0.61% (0.53–0.68)
Female	20.75 (19.07–22.02)	30.17 (26.71–33.15)	1.32% (1.16–1.47)	0.73 (0.66–0.82)	0.62 (0.55–0.68)	-0.59% (-0.63–0.56)	19.48 (17.33–21.7)	16.94 (14.72–18.62)	-0.57% (-0.62–0.52)
SDI quintile									
High SDI	27.18 (26.41–28.04)	38.87 (35.35–42.64)	1.42% (1.08–1.75)	0.56 (0.52–0.58)	0.47 (0.42–0.5)	-0.55% (-0.62–0.48)	13.87 (13.15–14.67)	12.29 (11.13–13.31)	-0.37% (-0.5–0.23)
High-middle SDI	16.7 (15.43–17.61)	24.89 (22.51–27.49)	1.44% (1.32–1.56)	0.6 (0.56–0.63)	0.47 (0.43–0.51)	-0.9% (-0.97–0.83)	15.45 (14.37–16.34)	12.35 (11.27–13.41)	-0.91% (-0.99–0.84)
Low SDI	5.6 (4.01–7.47)	9.65 (7.87–11.28)	1.92% (1.85–1.98)	0.73 (0.61–0.9)	0.72 (0.59–0.84)	-0.07% (-0.1–0.03)	21.13 (16.86–26.82)	19.57 (16.03–22.66)	-0.34% (-0.37–0.3)
Low-middle SDI	5.9 (4.81–7)	12.83 (10.91–14.42)	2.67% (2.61–2.73)	0.59 (0.52–0.7)	0.66 (0.58–0.73)	0.4% (0.32–0.48)	16.08 (13.93–19.28)	17.75 (15.52–19.65)	0.32% (0.24–0.4)
Middle SDI	7.95 (7.12–8.78)	18.73 (16.38–20.94)	3.1% (3.01–3.19)	0.56 (0.51–0.68)	0.59 (0.53–0.66)	0.39% (0.31–0.48)	14.33 (13.23–16.91)	15.02 (13.35–16.61)	0.3% (0.23–0.37)
GBD region									
Andean Latin America	8.97 (7.64–10.96)	25.39 (19.49–32.21)	3.77% (3.42–4.13)	0.95 (0.83–1.15)	1.13 (0.86–1.38)	0.81% (0.65–0.96)	23.71 (20.7–28.31)	26.74 (20.77–32.71)	0.54% (0.39–0.69)
Australasia	18.32 (16.67–20.14)	37.84 (29.42–49.03)	2.96% (2.58–3.34)	0.43 (0.4–0.45)	0.48 (0.4–0.51)	0.77% (0.6–0.94)	10.98 (10.27–11.91)	12.94 (10.98–14.56)	0.96% (0.78–1.14)
Caribbean	11.39 (10.47–12.24)	18.06 (15.04–21.7)	1.77% (1.66–1.87)	0.59 (0.53–0.64)	0.62 (0.52–0.72)	0.44% (0.23–0.65)	15.6 (13.97–17.16)	16.77 (13.87–19.77)	0.45% (0.25–0.66)
Central Asia	9.94 (8.79–11.54)	12.37 (10.95–13.97)	0.68% (0.32–1.03)	0.46 (0.41–0.54)	0.45 (0.4–0.49)	-0.38% (-0.59–0.16)	13.23 (11.44–15.38)	11.46 (10.27–12.79)	-0.88% (-1.11–0.65)
Central Europe	22.24 (20.28–23.27)	25.14 (21.75–29.15)	0.21% (0.13–0.29)	0.8 (0.72–0.83)	0.48 (0.42–0.56)	-2.02% (-2.2–1.84)	21.29 (18.56–22.17)	12.73 (11.04–14.75)	-2.08% (-2.28–1.89)
Central Latin America	10.31 (9.71–10.82)	20.98 (17.76–24.74)	2.36% (2.24–2.47)	0.84 (0.77–0.87)	0.82 (0.71–0.94)	-0.09% (-0.22–0.03)	20.24 (18.8–21)	20.04 (17.11–23.15)	-0.08% (-0.21–0.06)
Central Sub-Saharan Africa	2.51 (1.77–3.44)	3.49 (2.4–4.99)	1.12% (0.96–1.29)	0.51 (0.36–0.69)	0.49 (0.33–0.71)	-0.14% (-0.16–0.12)	12.69 (9.23–17)	11.63 (8.08–16.52)	-0.32% (-0.34–0.3)
East Asia	6.82 (5.65–8.02)	16.65 (13.89–20.23)	3.31% (3.16–3.45)	0.43 (0.38–0.53)	0.4 (0.33–0.46)	0.04% (-0.1–0.17)	11.07 (9.57–13.32)	9.86 (8.3–11.35)	-0.22% (-0.32–0.13)
Eastern Europe	17.35 (16.28–19.6)	35.09 (30.35–40.83)	2.84% (2.55–3.13)	0.51 (0.48–0.56)	0.55 (0.49–0.62)	0.04% (-0.26–0.33)	13.56 (12.68–15.18)	15.82 (14.08–17.81)	0.26% (-0.04–0.57)
Eastern Sub-Saharan Africa	8.67 (5.63–12.57)	12.43 (9.54–15.98)	1.16% (0.96–1.37)	1.16 (0.89–1.5)	1.04 (0.8–1.26)	-0.41% (-0.5–0.33)	34.72 (24.99–47.12)	27.78 (21.85–34.1)	-0.9% (-1–0.8)
High-income Asia Pacific	26.03 (24.49–29.37)	42.29 (35.62–49.33)	2.5% (1.66–3.34)	0.63 (0.58–0.72)	0.53 (0.43–0.58)	-0.45% (-0.75–0.16)	14.06 (13.16–16.49)	12.46 (10.69–13.99)	-0.05% (-0.53–0.44)
High-income North America	33.28 (32.16–34.32)	46.14 (39.61–53.68)	1.11% (0.91–1.3)	0.39 (0.37–0.41)	0.43 (0.4–0.45)	0.35% (0.27–0.43)	11.05 (10.27–11.88)	12.23 (11.1–13.34)	0.33% (0.22–0.43)
North Africa and Middle East	11.78 (9.72–13.61)	28.17 (22.9–32.54)	3.19% (3.11–3.27)	0.54 (0.44–0.74)	0.54 (0.47–0.66)	0.13% (0.03–0.24)	14.42 (11.8–17.96)	14.88 (12.81–17.09)	0.18% (0.09–0.28)
Oceania	7.57 (5.78–9.66)	10.49 (7.3–14.2)	0.97% (0.82–1.12)	0.74 (0.6–0.97)	0.78 (0.59–1.04)	0.23% (0.19–0.28)	18.15 (14.67–23.81)	19.04 (14.17–25.95)	0.18% (0.12–0.25)
South Asia	5.29 (4.38–6.56)	12.8 (10.66–14.9)	3.13% (3–3.26)	0.57 (0.49–0.72)	0.65 (0.56–0.74)	0.42% (0.26–0.57)	15.89 (13.63–20.27)	18.33 (15.98–20.88)	0.46% (0.31–0.6)
Southeast Asia	13.29 (10.65–15.36)	26.85 (21.32–31.59)	2.44% (2.36–2.51)	0.98 (0.86–1.12)	1.02 (0.88–1.15)	0.21% (0.14–0.28)	25.14 (21.35–28.51)	25.34 (21.37–28.88)	0.07% (0.01–0.12)
Southern Latin America	13.31 (12.08–14.42)	19.85 (15.25–25.71)	1.22% (1.09–1.35)	0.75 (0.68–0.79)	0.58 (0.53–0.62)	-0.99% (-1.16–0.81)	18.6 (16.84–19.58)	14.25 (13.18–15.48)	-1.03% (-1.22–0.83)
Southern Sub-Saharan Africa	5.81 (4.89–6.49)	6.78 (5.82–8.01)	0.52% (0.25–0.79)	0.45 (0.38–0.51)	0.48 (0.4–0.54)	0.29% (0.04–0.54)	11.81 (10.28–13.08)	11.96 (10.11–13.61)	0.19% (-0.04–0.43)
Tropical Latin America	8.98 (8.54–9.53)	13.14 (12.3–14.55)	1.33% (0.98–1.68)	0.69 (0.65–0.72)	0.52 (0.48–0.58)	-0.81% (-0.91–0.71)	16.74 (16.04–17.61)	12.82 (12.02–14.57)	-0.82% (-0.95–0.69)
Western Europe	28.73 (26.93–29.91)	33.21 (28.61–37.98)	0.39% (0.16–0.62)	0.67 (0.63–0.7)	0.46 (0.41–0.48)	-1.49% (-1.55–1.43)	16.53 (15.27–17.32)	11.66 (10.59–12.79)	-1.32% (-1.37–1.28)
Western Sub-Saharan Africa	1.41 (1.13–1.68)	2.18 (1.68–2.73)	1.61% (1.5–1.71)	0.24 (0.18–0.28)	0.23 (0.18–0.27)	-0.06% (-0.1–0.03)	5.89 (4.62–6.89)	5.67 (4.43–6.7)	-0.13% (-0.16–0.1)

The top three countries in terms of the number of incident cases of TC remained consistent throughout the study period: the US (11,689 in 1990 and 26,270 in 2019), China (10,030 in 1990 and 39,079 in 2019), and India (5,988 in 1990 and 23,833 in 2019) (**Supplementary Table S6**). China also had the highest number of deaths associated with thyroid cancer globally (3,318; 95%UI: 2,861–4,133 in 1990; 7,239; 95%UI: 6,011–8,476 in 2019) (**Supplementary Table S2**). However, there was a change in the country with the highest thyroid cancer DALYs, moving away from China (103,492.62; 95% CI: 87,958.27–124,715.04) in 1990 to India (217,465.02; 95% CI: 181,111.87–254,845.52) in 2019 (**Supplementary Table S5**).

### Thyroid cancer prevalence

**Figure 2:**
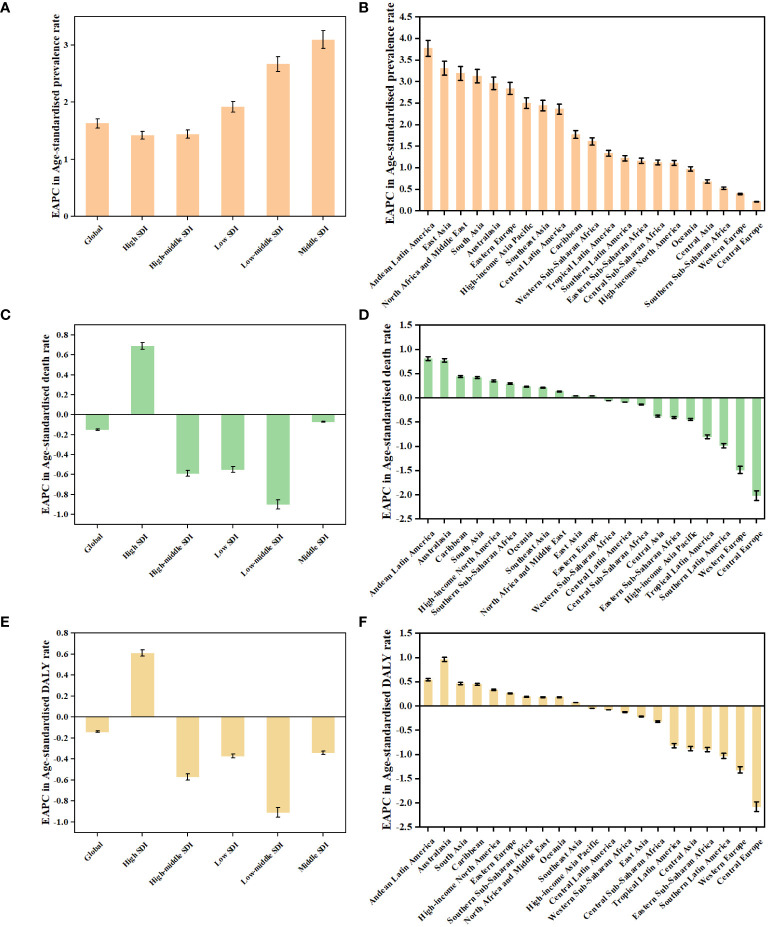
The EAPCs in age-standardised rates of prevalence **(A, B)**, deaths **(C, D)**, and DALYs **(E, F)** due to TC from 1990 to 2019, both sexes, by GBD region and by SDI quintile. DALYs, disability-adjusted life-years; EAPC, estimated annual percentage change; GBD, Global Burden of Disease; TC, thyroid cancer; SDI, Socio-demographic Index.

**Figure 3:**
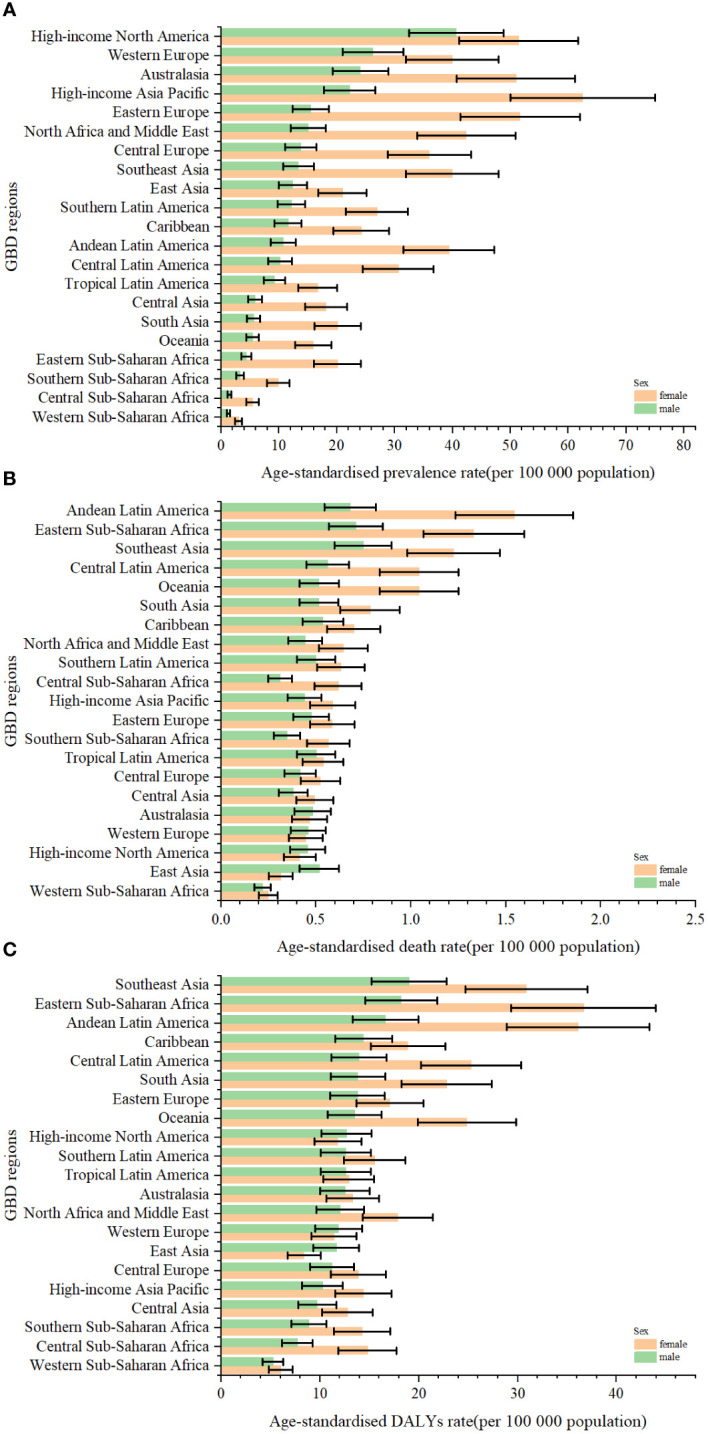
The age-standardised rates of prevalence **(A)**, deaths **(B)**, and DALYs **(C)** due to TC by sex, across 21 regions, in 2019. Error bars indicate the 95% uncertainty interval (UI) for the age-standardised rates. DALYs, disability-adjusted life-years; GBD, Global Burden of Disease; TC, thyroid cancer.

**Figure 4:**
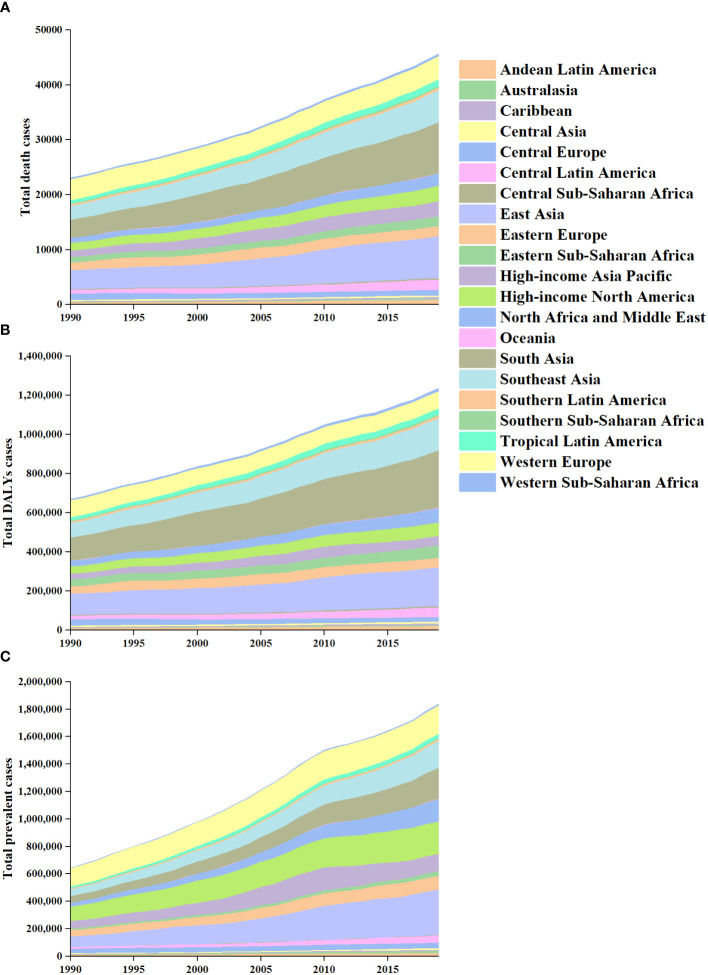
The absolute number of prevalent cases **(A)**, deaths **(B)**, and DALYs **(C)** due to TC for all GBD regions from 1990 to 2019. DALYs, disability-adjusted life-years; GBD, Global Burden of Disease; TC, thyroid cancer.

**Figure 5:**
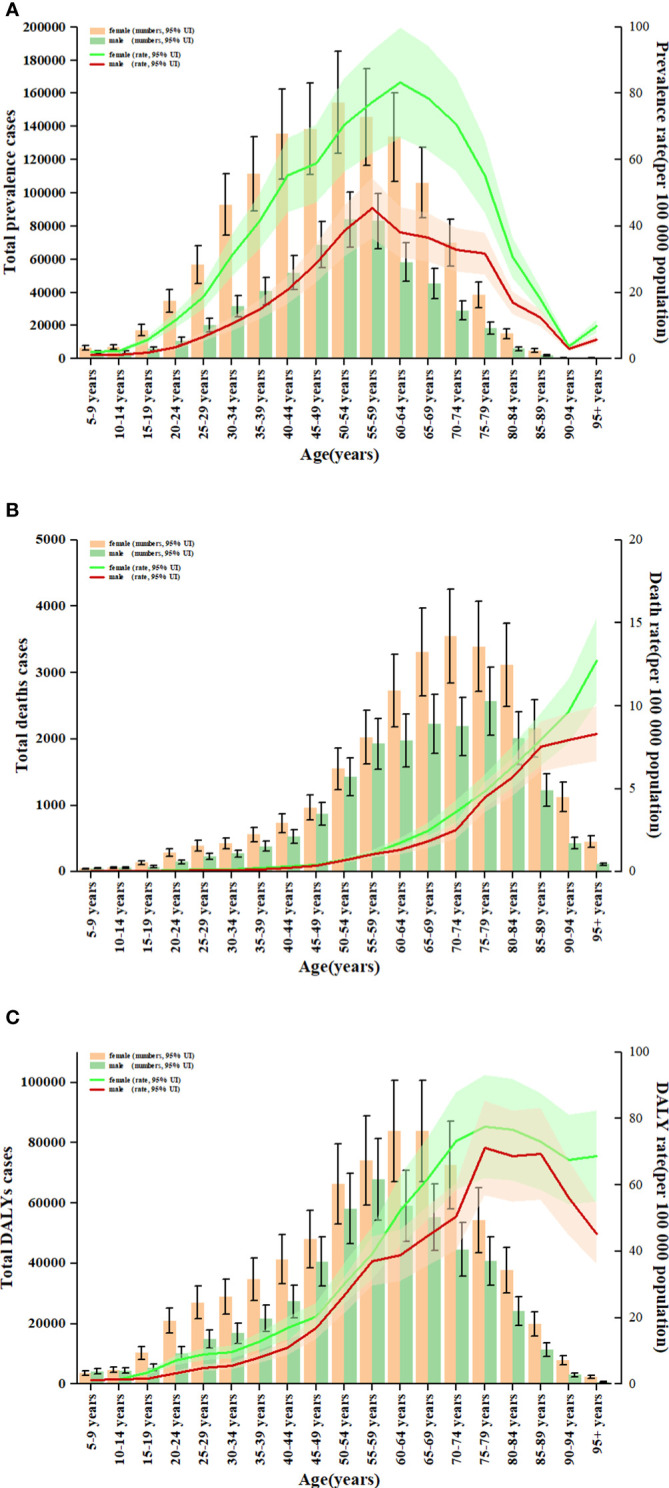
Age patterns by sex of the total number and age-standardised rates of prevalent cases **(A)**, deaths **(B)** and DALYs **(C)** due to TC at the global level in 2019. Error bars indicate the 95% uncertainty interval (UI) for the number of cases. Shading indicates the 95% UI for the rates. DALYs, disability-adjusted life-years; TC, thyroid cancer.

Globally, TC accounted for an estimated 6.39 million cases (95% UI 5.96–6.71) in 1990, increasing to 18.31 million cases (16.56–19.85) in 2019, representing a 3.78% increase over this period (**Supplementary Table S1**; **Figure 1A**). The global age-standardized prevalence rate of TC rose from 14.06 per 100,000 people (13.19–14.74) in 1990 to 22.02 per 100,000 people (19.91–23.86) in 2019, with an EAPC of 1.63% (95% CI 1.48–1.78) (**Table 1**; **Figures 1B, C**, **2A**). While visual representations indicated consistent data quality on a global scale, it’s important to exercise caution due to the reliance on limited data in specific regions. In 2019, The highest number of prevalent cases was observed in East Asia (3.30 million; 95% UI 2.74 - 4.03) (**Supplementary Table S1**), while the high-income North America region recorded the highest age-standardized prevalence rate (46.14 per 100,000 population; 39.61 - 53.68) (**Figure 3A**). Across most GBD regions, an increase in total prevalent cases was observed from 1990 to 2019 (**Figure 4A**), along with a rise in age-standardized prevalence rates in all GBD regions, with the highest increase seen in Andean Latin America (EAPC:3.77%; 95%CI 3.42–4.13) and the lowest in Central Europe (EAPC:0.21%; 0.13–0.29) (**Figure 2B**). In 2019, China and the USA had the highest number of prevalent cases at the national and territorial levels (310,327; 95% UI 255,040–382,138 and 220,711; 187,749–257,904, respectively) (**Supplementary Table S2**; **Figure 1A**). Iceland had the highest age-standardized prevalence rates of TC in 2019 (71.43; 59.51–84.72) (**Supplementary Table S3**; **Figure 1B**). During the period from 1990 to 2019, age-standardized prevalence rates increased across all 204 countries or territories, with Saudi Arabia showing the most significant rise. (EAPC:6.45%; 95% CI 6.12–6.78) (**Figure 1C**). In 2019, the prevalence of thyroid cancer cases was higher among females than males overall (**Supplementary Table S1**). In 2019, the peak prevalence and age-specific rates were observed in the 50 to 54 years and 60 to 64 years age groups for both females and males. (**Figure 5A**). Prevalent cases continued to rise for both sexes from 1990 to 2019, with age-standardized prevalence rates increasing across the board (**Supplementary Figure S1**).

### Thyroid cancer deaths

In 2019, thyroid cancer (TC) caused 22,966 deaths worldwide (95% UI 21,554–25,228), marking a 98% increase (75% to 114%) from 11,689 deaths (95% UI 10,823–12,672) in 1990 (**Supplementary Table S1**; **Supplementary Figure S2**). The global age-standardized death rate stood at 0.60 per 100,000 population (0.56–0.66) in 2019, up from 0.57 per 100,000 people (0.51–0.61) in 1990, with EAPC of -0.15% (95% CI -0.19–0.11) (**Table 1**; **Figures 2C**, **3**, **4**). At the regional level, South Asia recorded the highest number of deaths (9,195; 95% UI 7,978–10,476), while Andean Latin America reported the highest age-standardized death rate (1.13 per 100,000 population; 0.86–1.38) in 2019 (**Supplementary Table S1**; **Figure 3B**). Over the period spanning from 1990 to 2019, most GBD regions saw an increase in total deaths from TC (**Figure 4B**). However, the age-standardized death rate decreased in over half of the GBD regions, including Central Europe [EAPC:-2.02 (95% CI -2.2–1.84)], Western Europe (EAPC:-1.49 (-1.16–0.81)), and Tropical Latin America [EAPC:-0.81 (-0.91–0.71)] (**Figure 2D**). In 2019, China and India recorded the highest death tolls at the national and territorial levels (7,239; 95% UI 6,011–8,476 and 7,074; 5,978–8,314, respectively) (**Supplementary Table S2**; **Supplementary Figure S2**). In 2019, Honduras reported the highest age-standardized death rate (3.17 per 100,000 population; 0.95–4.35) (**Supplementary Table S3**; **Supplementary Figure S3**). During the period from 1990 to 2019, more than half of the countries or territories observed a decline in the age-standardized death rate related to thyroid cancer, with the largest increase noted in Armenia (EAPC:3.03; 2.34–3.72) (**Supplementary Figure S4**). In 2019, the global mortality rate due to thyroid cancer was higher among females compared to males(**Supplementary Table S2**). The age-standardized death rate was 0.43 per 100,000 population (95% UI 0.4–0.48) among males and 0.73 per 100,000 population (0.66–0.82) among females (**Table 1**). In 2019, males aged 75–79 years and females aged 70–74 years exhibited the highest number of deaths and the highest age-standardized death rate due to thyroid cancer (**Figure 5B**). There was a consistent increase in deaths related to TC for both sexes from 1990 to 2019, with females experiencing higher numbers throughout the entire period. However, there was a decrease in the age-standardized death rate for both sexes during this timeframe (**Supplementary Figure S5**).

### Thyroid cancer DALYs

Globally, thyroid cancer (TC) resulted in 6.67 million DALYs (95% UI 6.13–7.32), comprising 0.41 million YLD (0.28 to 0.56) and 6.26 million YLL (5.77 to 6.88) in 2019, reflecting an increase of 29.98 percent, 47.96 percent, and 18.31 percent, respectively, from 1990 (**Supplementary Tables S1**, **S4**; **Supplementary Figures S6–S9**). The age-standardized DALY rate globally declined from 15.55 per 100,000 population (14.4–17.02) in 1990 to 14.98 per 100,000 population (13.55–16.14) in 2019, showing an EAPC of -0.14% (95% CI -0.18–0.1) (**Table 1**; **Figure 2E**; **Supplementary Figures S10, S11**). Similar declining trends were observed in the age-standardized YLL rate [EAPC=-0.26% (-0.3–0.23)] and the age-standardized YLD rate [EAPC=1.39% (1.26–1.53)] (**Supplementary Table S5**; **Supplementary Figures S12–S14**). At the regional scale, South Asia recorded the highest count of DALYs (291,574; 95% UI 254,403–330,796) in 2019 (**Supplementary Table S1**). In 2019, Eastern Sub-Saharan Africa exhibited the highest age-standardized DALY rates (27.78 per 100,000 population; 95% UI 21.85–34.1) (**Figure 3C**). Total DALYs increased across most GBD regions from 1990 to 2019 (**Figure 4C**). Nonetheless, the age-standardized DALY rate decreased across the majority of GBD regions, with Central Europe experiencing the most substantial decline. [EAPC:-2.08% (95% CI -2.28–1.89)] (**Figure 2F**). In 2019, India and China recorded the highest DALY numbers at the national level (217,465; 95% UI 181,111–254,845 and 187,318; 156,236–219,112, respectively) (**Supplementary Table S2**; **Supplementary Figure S6**). Honduras had the highest age-standardized DALY rate in 2019 (76.23 per 100,000 people; 23.25–108.35) (**Supplementary Table S3**; **Supplementary Figure S10**). More than 54% of countries or territories experienced a decline in the age-standardized DALY rate related to thyroid cancer, with Poland showing the most substantial decrease. [EAPC:-3.34% (95% CI -3.76–2.92)] (**Supplementary Figure S11**). In 2019, the global DALYs were higher among females than males (**Supplementary Table S1**). The age-standardized DALY rate was 12.93 per 100,000 population (95% UI 11.7–14.02) among males and 16.94 per 100,000 population (14.72–18.62) among females (**Table 1**). The peak number of DALYs was observed among females aged 60–64 years and 65–69 years, and among males aged 55–59 years. (**Figure 5C**). YLL was highest in the age group 60–64 years (142,963; 129,900–153,911), while YLD was highest in the age group 50–54 years (13,966; 9,535–19,341) (**Supplementary Figure S15**). DALYs increased for both sexes from 1990 to 2019, with higher rates among females throughout this period. However, the age-standardized DALY rate decreased for both genders throughout this period (**Supplementary Figure S16**).

### Burden of thyroid cancer by SDI

**Figure 6:**
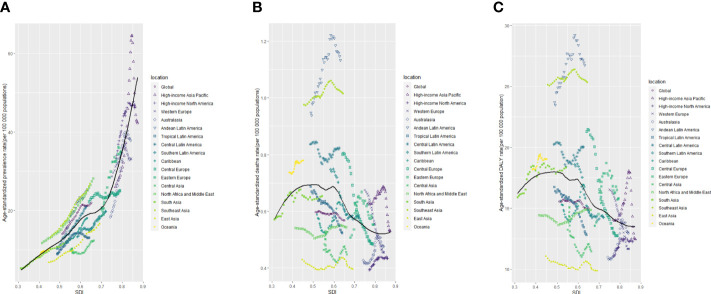
The age-standardised rates of TC prevalence **(A)**, deaths **(B)**, and DALYs **(C)** globally and for 21 GBD regions by SDI from 1990 to 2019. The expected age-standardised rates in 2019 based solely on SDI were represented by the black line. For each region, points from left to right depict estimates from each year from 1990 to 2019. DALYs, disability-adjusted life-years; GBD, Global Burden of Disease; TC, thyroid cancer; SDI, Socio-demographic Index.

**Figure 7:**
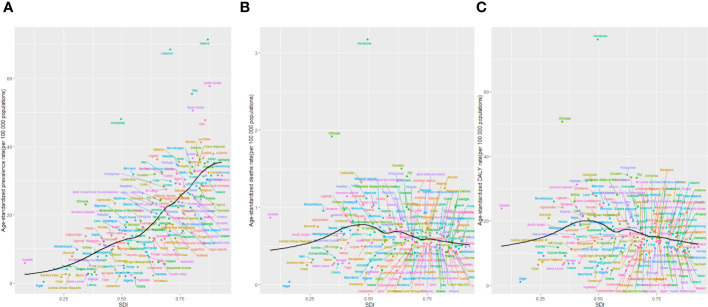
The age-standardised rates of TC prevalence **(A)**, deaths **(B)**, and DALYs **(C)** globally and for 204 territories by SDI from 1990 to 2019. The expected age-standardised rates in 2019 based solely on SDI were represented by the black line. For each region, points from left to right depict estimates from each year from 1990 to 2019. DALYs, disability-adjusted life-years; GBD, Global Burden of Disease; TC, thyroid cancer; SDI, Socio-demographic Index.

Higher levels of SDI were linked to increased age-standardized prevalence rates of TC, exceeding the global rate in the high and high-middle SDI quintiles, and remaining lower in the other three SDI quintiles (**Table 1**). In 2019, the high-SDI quintile showed the highest age-standardized prevalence rate, while the low-SDI quintile reported the highest age-standardized death rate and DALY rate. From 1990 to 2019, the age-standardized death rate and DALY rate decreased in all SDI quintiles except the high-SDI quintile, where they increased [EAPC:0.82 (95% CI 0.64–1.01)] and remained stable [EAPC: -0.55 (95% CI -0.62–0.48)], respectively (**Table 1**; **Figures 2C, E**). On the contrary, the age-standardized prevalence rate increased across all SDI quintiles, whereas the age-standardized death rate and DALY rate decreased in the High SDI, High-middle SDI, and Low SDI quintiles but rose in the remaining two SDI quintiles during the study period(**Table 1**; **Figure 2A**). The global and regional age-standardized prevalence, mortality, and DALY rates related to SDI are illustrated in **Figure 6**, spanning the annual time series from 1990 to 2019. Generally, regions showed an increase in mortality and DALY rates with higher SDI levels. However, more than 60% of regions displayed an upward trend in the age-standardized prevalence rate over the study period. Globally, the age-standardized mortality and DALY rates decreased as SDI values increased but remained higher than expected levels over the past three decades. In 2019, a negative correlation was observed between age-standardized DALY rates for TC and SDI at the national level, with some exceptions (**Figure 7**).

## Discussion

We noted a significant global rise in both thyroid cancer incidence and prevalence over the 30-year study period, alongside a notable decrease in deaths and DALYs. Our findings also highlighted a positive correlation between thyroid cancer prevalence and the SDI level. However, such associations were not evident concerning deaths and DALYs attributed to thyroid cancer. Females across various age groups exhibited higher prevalence, deaths, and DALYs of thyroid cancer compared to males. Notably, fatalities related to thyroid cancer were predominantly concentrated in the 65–79-year-old age group for both genders. Compared with previous GBD studies (23–26), our research utilized current epidemiological data to investigate thyroid cancer’s burden and its connection to SDI levels across a broader spectrum of locations, covering 204 countries and territories. This underscores the importance of timely epidemiological analysis in providing evidence to guide strategic planning for healthcare services and resource allocation aimed at addressing thyroid cancer.

Between 1990 and 2019, there was an increasing trend in the ASPR, contrasting with the declining trends observed in ASMR and ASDR. This evolving landscape in thyroid cancer incidence can be attributed to several factors. Firstly, advancements in diagnostic technologies have led to the inadvertent discovery of more occult thyroid carcinomas (27, 28). Secondly, advancements in socioeconomic status and heightened health awareness have led to an increased rate of early detection for thyroid cancer. Thirdly, overdiagnosis has notably contributed to the rise in thyroid cancer prevalence (29). Moreover, factors including but not limited to family history, obesity, dietary habits (such as red meat and processed food consumption), iodine intake, psychological factors, and environmental pollutants could impact the prevalence of thyroid cancer (30). Furthermore, in our observations, females exhibited higher rates of prevalence, mortality, and DALYs compared to males across various age groups. Thyroid cancer is thought to be influenced by abnormal levels of estrogen in females and unhealthy dietary habits (31, 32). Moreover, women’s increased healthcare engagement, influenced by reproductive and perimenopausal factors, likely leads to more frequent thyroid examinations (28), contributing to the observed higher prevalence rates among females.

Previous research has highlighted differences in thyroid cancer outcomes based on gender and socioeconomic status. While research indicated that males residing in lower socioeconomic communities exhibited lower thyroid cancer-specific survival rates, this pattern was not observed among females (29). Similarly, research by Nilubol et al (30) Studies have suggested that males diagnosed with thyroid cancer often present with advanced stages of the disease, resulting in earlier cause-specific deaths, potentially attributed to biological and behavioral disparities in accessing medical care (32). However, well-known risk factors such as radiation exposure and familial history did not fully explain the increased prevalence (33). Furthermore, reproductive factors such as menstrual, reproductive, or hormonal history showed no significant association with thyroid cancer risk (31, 34, 35). Another study observed differences in estrogen receptor subtypes depending on the histologic factors of thyroid cancer (36).

We noticed substantial differences in thyroid cancer prevalence among various regions and countries. In 2019, East Asia and North America stood out with the highest incidence of thyroid cancer cases, which can be attributed in part to their sizable populations. Notably, Iceland recorded the highest ASPR globally, possibly due to a screening program initiated in 1999 (37). The intensive monitoring and use of advanced diagnostic technologies, such as ultrasound, likely contribute to higher detection rates in Iceland (38). Our analysis also showed a positive correlation between the SDI and ASPR of thyroid cancer prevalence at both regional and national levels. This suggests that regions with higher SDI levels often have increased prevalence rates of thyroid cancer, possibly due to advanced healthcare infrastructure and greater public health awareness facilitating early detection. Moreover, those with elevated socioeconomic status tend to have greater access to healthcare services and are more engaged with the healthcare system, thereby contributing to the observed correlation between economic status and the burden of thyroid cancer (39).

To our understanding, this study presents the latest epidemiological patterns concerning thyroid cancer’s impact, encompassing global, regional, and national perspectives across various demographic factors such as gender, age, and SDI categories. Notably, Asia bears the highest burden of thyroid cancer, whereas Oceania exhibits the lowest burden. The typical age of onset for individuals developing thyroid cancer has decreased, while the age at which those with thyroid cancer pass away has increased worldwide. Moreover, individuals in lower SDI quintiles tend to develop and succumb to thyroid cancer at earlier ages compared to those in higher quintiles. Additionally, growth patterns in thyroid cancer incidence vary significantly between genders and seem to exhibit a reversal during the later years of the study timeframe (40, 41).

## Conclusion

In this study, the prevalence, deaths, DALYs, and ASIR for thyroid cancer exhibited a global increase, indicating a heightened burden on healthcare systems worldwide, especially among females and in countries with a high SDI. However, there was a decrease noted in the ASDR and age-standardized DALY rate for thyroid cancer, possibly linked to advancements in therapeutic strategies. The variability in thyroid cancer burden across different categories underscores the influence of diverse genetic and environmental risk factors, alongside variations in socioeconomic status, educational attainment, lifestyle choices, and availability of medical screening and treatment services. Further exploration is needed to uncover the intricate mechanisms behind these factors. In summary, the prevalence of thyroid cancer worldwide has shown a consistent increase over the last thirty years. Our examination of the epidemiological patterns in thyroid cancer can provide valuable insights for decision-makers in resource allocation and policy formulation to ensure more equitable distribution of limited resources.

## Data availability statement

The original contributions presented in the study are included in the article/**Supplementary Material**. Further inquiries can be directed to the corresponding authors.

## Author contributions

ZD: Data curation, Formal analysis, Investigation, Methodology, Software, Visualization, Writing – original draft. YS: Funding acquisition, Project administration, Writing – review & editing. JJ: Project administration, Writing – review & editing.
