# Supplementary material for: Global burden of disease study analysis of thyroid cancer burden across 204 countries and territories from 1990 to 2019
Source: Front Oncol. 2024 May 28;14:1412243. doi: 10.3389/fonc.2024.1412243 (PMC11175622; doi:10.3389/fonc.2024.1412243)
Supplement: Supplementary file 1 [file DataSheet_1.docx]

**Supplement to: The global, regional and national burden of Thyroid cancer in 204 countries and territories from 1990 to 2019, based on Global Burden Disease Study 2019**

**Contents:**

**Supplementary Figures**

Supplementary Figure S1: Trends from 1990 to 2019 in number and age-standardised prevalence rate of TC at the global level.

Supplementary Figure S2: The absolute number of deaths due to TC for 204 countries and territories in 2019.

Supplementary Figure S3: The age-standardised death rate (per 100 000 population) of TC for 204 countries and territories in 2019.

Supplementary Figure S4: The EAPC of age-standardised death rate for TC in 204 countries and territories between 1990 and 2019.

Supplementary Figure S5: Trends from 1990 to 2019 in number and age-standardised death rate of TC at the global level.

Supplementary Figure S6: The absolute number of DALYs due to TC for 204 countries and territories in 2019.

Supplementary Figure S7: The absolute number of incident cases due to TC for 204 countries and territories in 2019.

Supplementary Figure S8: The absolute number of YLLs due to TC for 204 countries and territories in 2019.

Supplementary Figure S9: The absolute number of YLDs due to TC for 204 countries and territories in 2019.

Supplementary Figure S10: The age-standardised DALY rate (per 100 000 population) of TC for 204 countries and territories in 2019.

Supplementary Figure S11: The EAPC of age-standardised DALY rate for TC in 204 countries and territories between 1990 and 2019.

Supplementary Figure S12: The EAPC of age-standardised incidence rate for TC in 204 countries and territories between 1990 and 2019.

Supplementary Figure S13: The EAPC of age-standardised YLL rate for TC in 204 countries and territories between 1990 and 2019.

Supplementary Figure S14: The EAPC of age-standardised YLD rate for TC in 204 countries and territories between 1990 and 2019.

Supplementary Figure S15: Global counts and age-specific rates of YLLs and YLDs due to TC across age group in 2019.

Supplementary Figure S16: Trends from 1990 to 2019 in number and age-standardised DALY rate of TC at the global level.

**Supplementary Tables**

Supplementary Table S1: Prevalent cases, deaths and disability adjusted life years (DALYs) of Thyroid cancer (TC) in 1990 and 2019 and their percentage change from 1990 to 2019

Supplementary Table S2: Prevalent cases, deaths and disability adjusted life years (DALYs) for Thyroid cancer (TC) by 204 countries or territories in 2019 and 2019, and their percentage change from 1990 to 2019.

Supplementary Table S3: Age-standardised prevalence, death and DALY rates for Thyroid cancer (TC) by 204 countries or territories in 1990 and 2019, and their temporal trends from 1990 to 2019.

Supplementary Table S4: Incident cases, YLLs and YLDs of Thyroid cancer (TC) in 1990 and 2019 and their percentage change from 1990 to 2019.

Supplementary Table S5: Age-standardised incidence, YLL and YLD rates for Thyroid cancer (TC) in 1990 and 2019 and their temporal trends from 1990 to 2019.

**Supplementary Figure S1:** Trends from 1990 to 2019 in number and age-standardised

prevalence rate of TC at the global level.


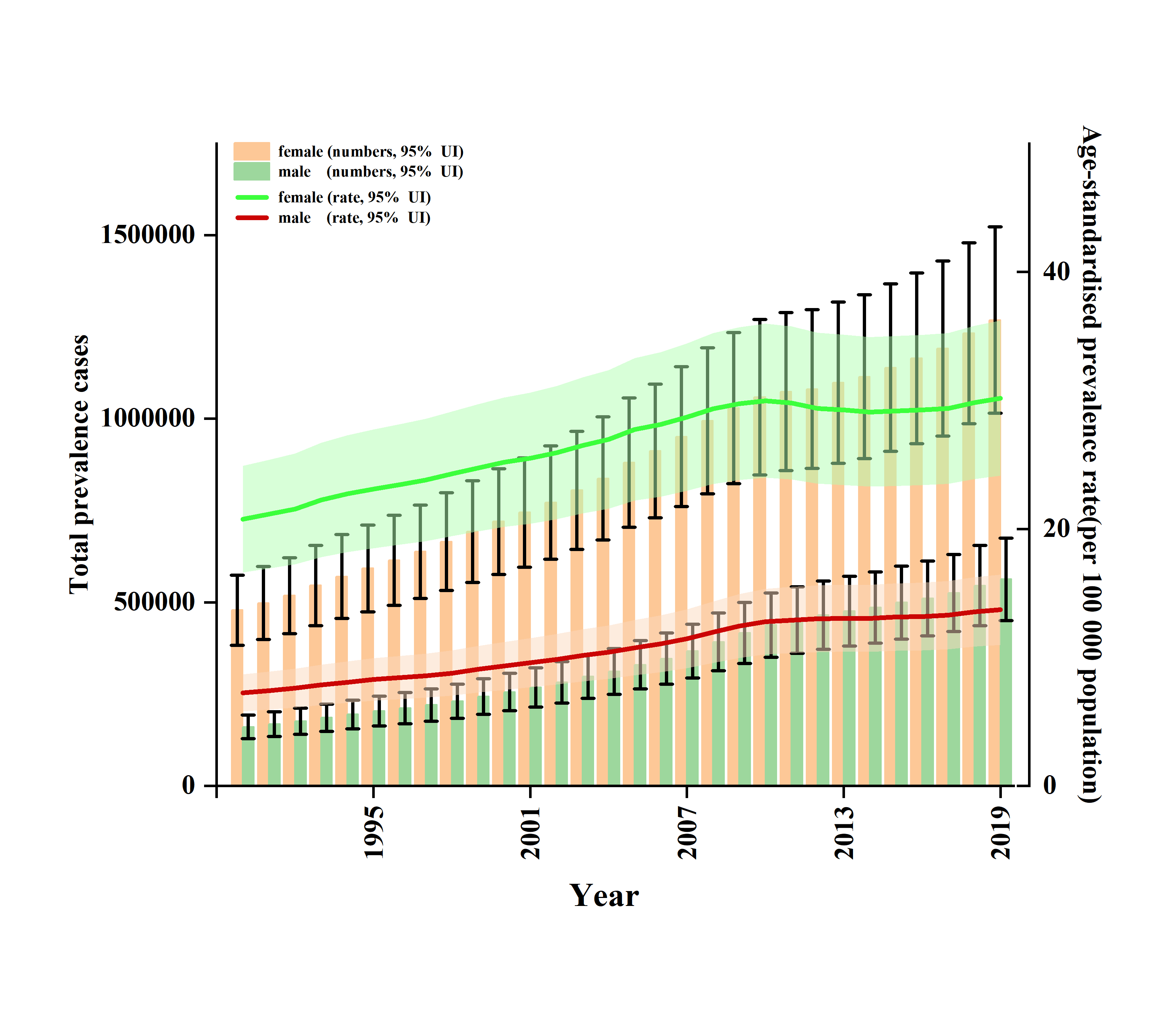


Error bars indicate the 95% uncertainty interval (UI) for number of prevalent cases. Shading indicates the 95% UI for the age-standardised prevalence rate. TC, thyroid cancer.

**Supplementary Figure S2:** The absolute number of deaths due to TC for 204 countries and territories in 2019.


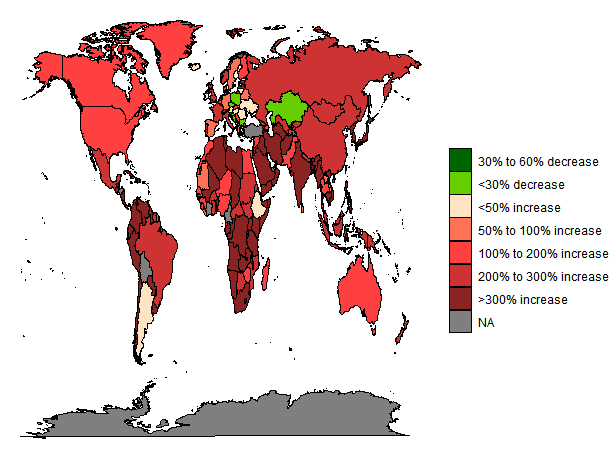


Thyroid cancer,TC

**Supplementary Figure S3:** The age-standardised death rate (per 100 000 population) of TC for 204 countries and territories in 2019.


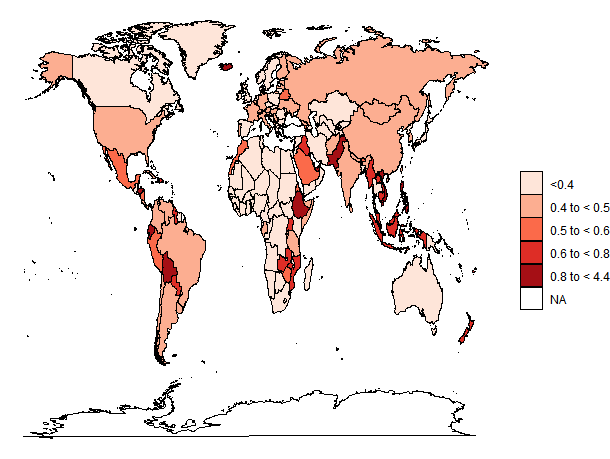


Thyroid cancer,TC

**Supplementary Figure S4:** The EAPC of age-standardised death rate for TC in 204 countries and territories between 1990 and 2019.


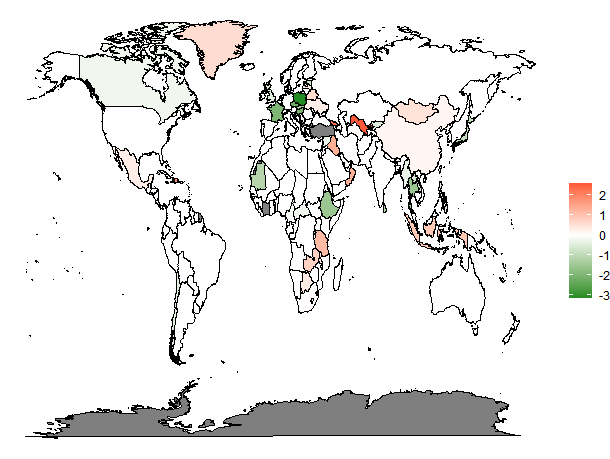
EAPC, estimated annual percentage change;Thyroid cancer,TC

**Supplementary Figure S5:**Trends from 1990 to 2019 in number and age-standardised death rate of TC at the global level.


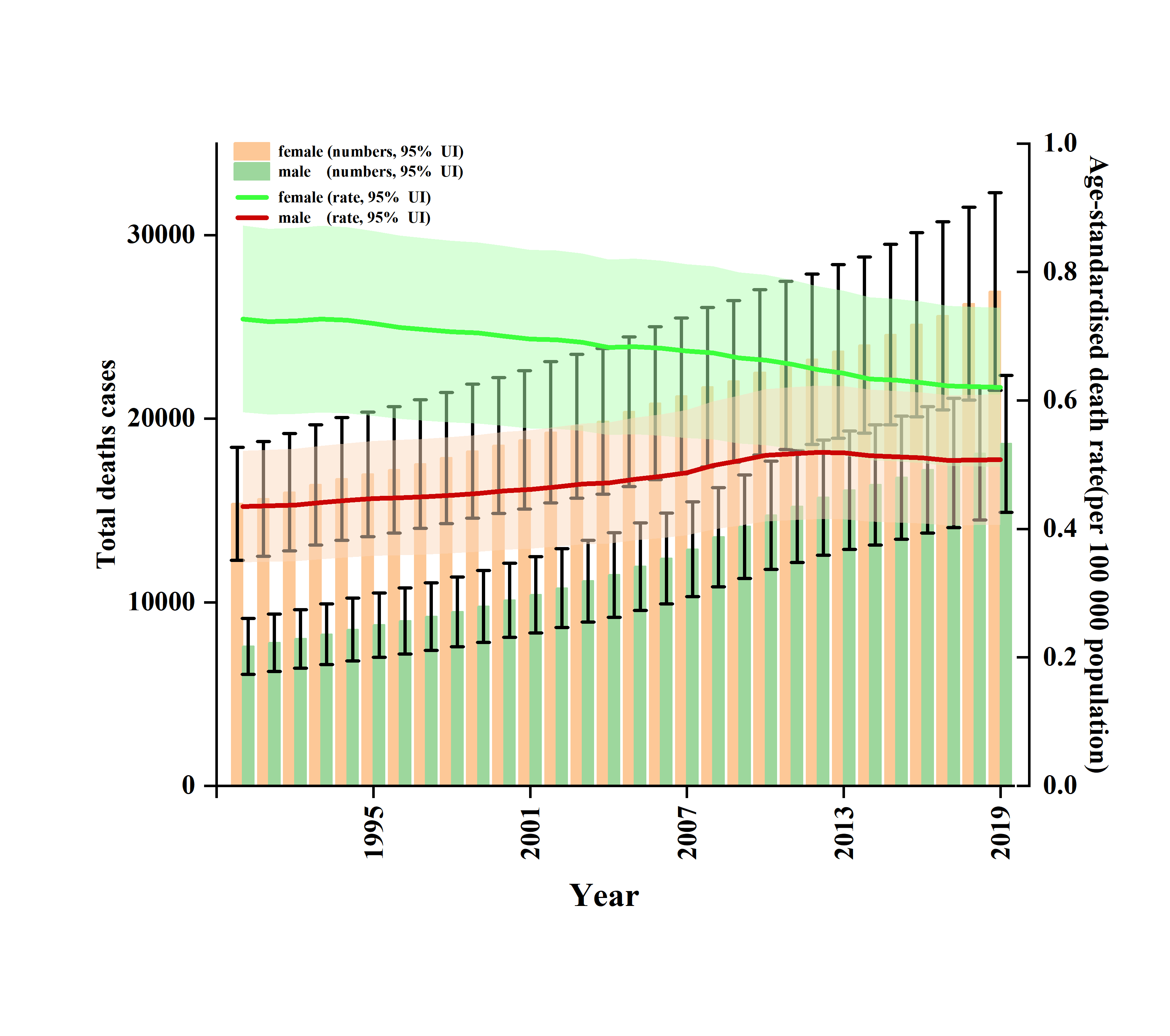


Error bars indicate the 95% uncertainty interval (UI) for number of death cases. Shading indicates

the 95% UI for the age-standardised death rate. TC, thyroid cancer.

**Supplementary Figure S6:** The absolute number of DALYs due to TC for 204 countries and territories in 2019.


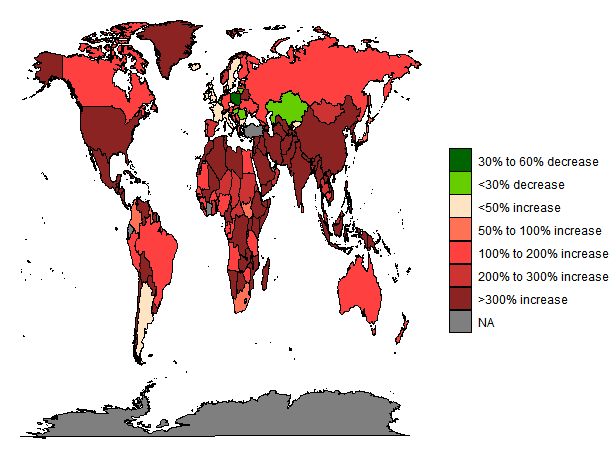


DALYs, disability adjusted life years; Thyroid cancer,TC

**Supplementary Figure S7:** The absolute number of incident cases due to TC for 204 countries and territories in 2019.


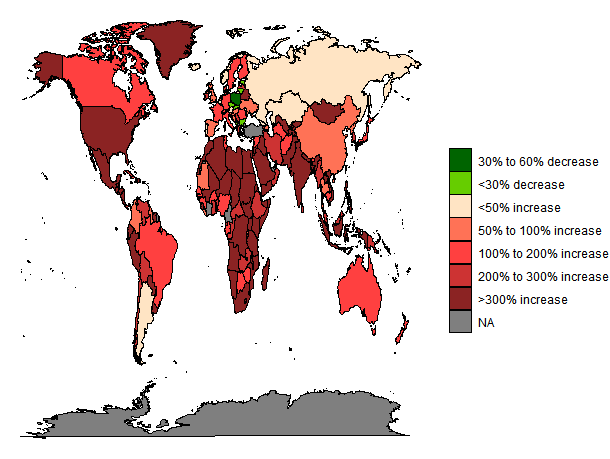


Thyroid cancer,TC

**Supplementary Figure S8:** The absolute number of YLLs due to TC for 204 countries and territories in 2019.


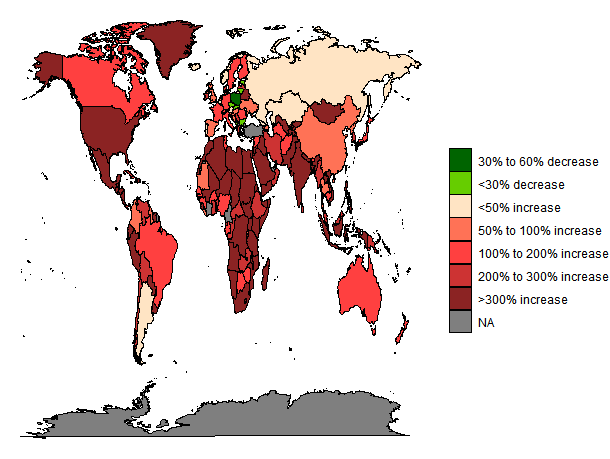


Thyroid cancer,TC;YLLs, years of life lost.

**Supplementary Figure S9:** The absolute number of YLDs due to TC for 204 countries and territories in 2019.


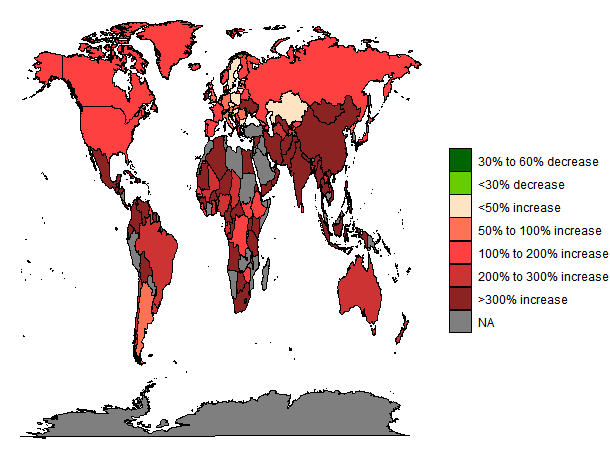


Thyroid cancer,TC;YLDs, years of life lived with disability.

**Supplementary Figure S10:** The age-standardised DALY rate (per 100 000 population) of TC for 204 countries and territories in 2019.


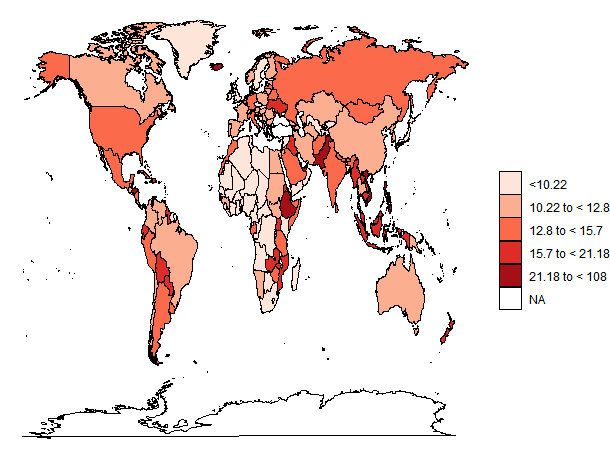


DALY, disability adjusted life year; Thyroid cancer,TC

**Supplementary Figure S11:** The EAPC of age-standardised DALY rate for TC in 204 countries and territories between 1990 and 2019.


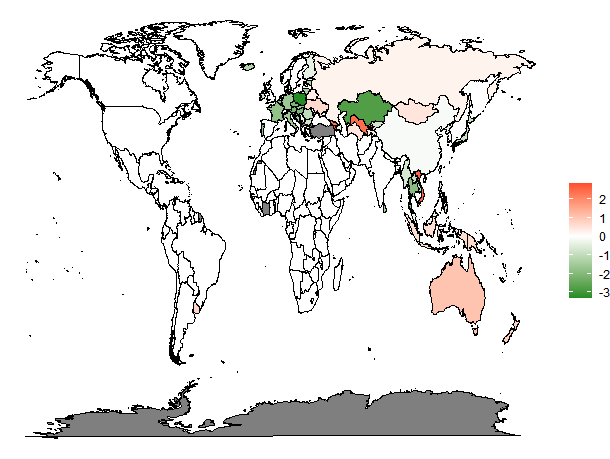


DALY, disability adjusted life year; EAPC, estimated annual percentage change;Thyroid cancer,TC

**Supplementary Figure S12:** The EAPC of age-standardised incidence rate for TC in 204 countries and territories between 1990 and 2019.


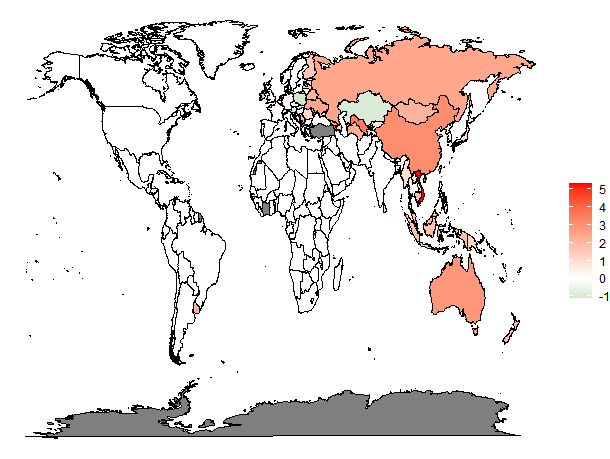


EAPC, estimated annual percentage change;Thyroid cancer,TC

**Supplementary Figure S13:** The EAPC of age-standardised YLL rate for TC in 204 countries and territories between 1990 and 2019.


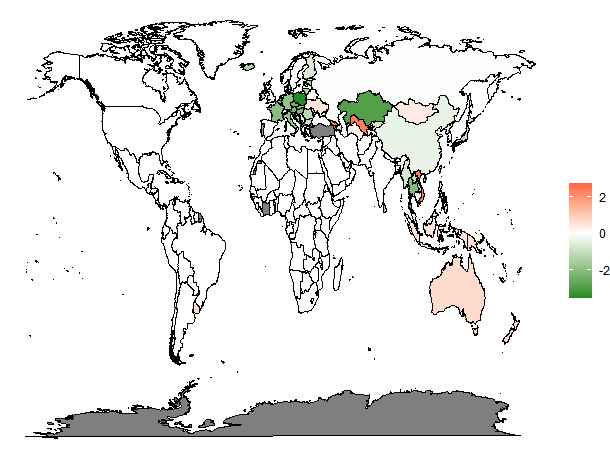


EAPC, estimated annual percentage change;Thyroid cancer,TC; YLL, years of life lost.

**Supplementary Figure S14:** The EAPC of age-standardised YLD rate for TC in 204 countries and territories between 1990 and 2019.


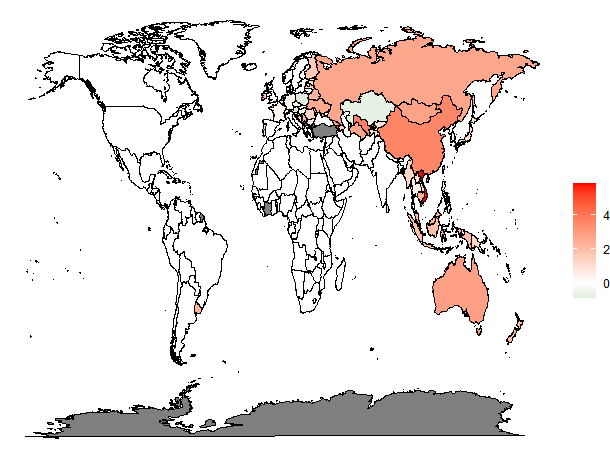


EAPC, estimated annual percentage change;Thyroid cancer,TC; YLD, years of life lived with disability.

**Supplementary Figure S15:** Global counts and age-standardised rates of YLLs and YLDs due to TC across age group in 2019.


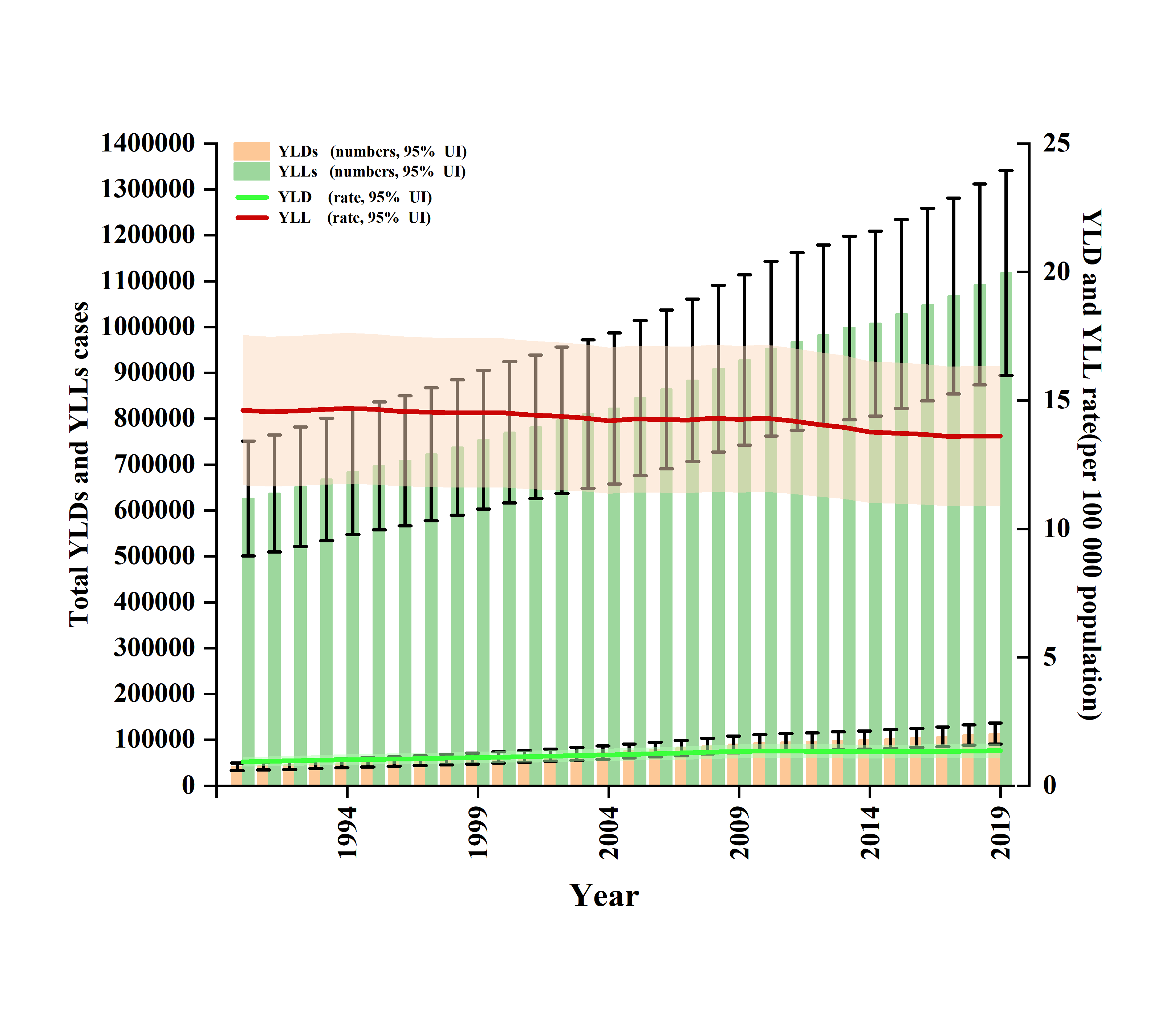


Error bars indicate the 95% uncertainty interval (UI) for YLLs and YLDs. Shading indicates the 95% UI for the rates. TC, thyroid cancer; YLDs, years of life lived with disability; YLLs, years of life lost.

**Supplementary Figure S16:**Trends from 1990 to 2019 in number and age-standardised DALY rate of TC at the global level.


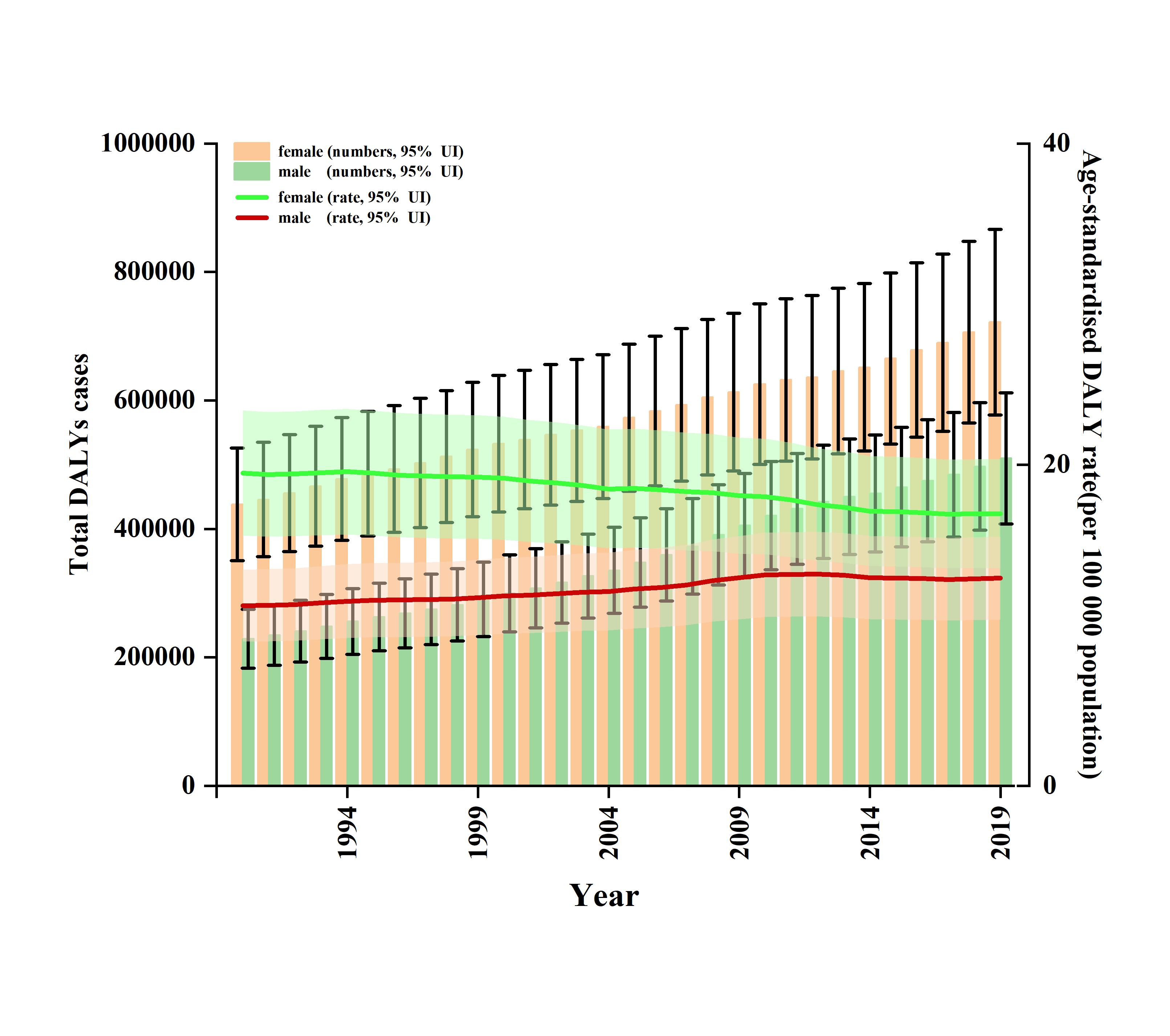


Error bars indicate the 95% uncertainty interval (UI) for number of DALYs cases. Shadingindicates the 95% UI for the age-standardised DALY rate. DALY, disability adjusted life year; TC, thyroid cancer.

**Supplementary Table S1:** Prevalent cases, deaths and disability adjusted life years (DALYs) of Thyroid cancer (TC) in 1990 and 2019 and their percentage change from 1990 to 2019

|  | **Prevalent cases** | | | **Deaths** | | | **DALYs** | | |
| --- | --- | --- | --- | --- | --- | --- | --- | --- | --- |
| **Characteristics** | **1990no**  **(95%UI)** | **2019no**  **(95%UI)** | **Percentage**  **change**  **(95%UI)** | **1990no**  **(95%UI)** | **2019no**  **(95%UI)** | **Percentage**  **change**  **(95%UI)** | **1990no**  **(95%UI)** | **2019no**  **(95%UI)** | **Percentage**  **change**  **(95%UI)** |
| Global | 639050 (596165-671632) | 1831760 (1656127-1985013) | 1.87 (1.61-2.15) | 22966 (21554-25228) | 45576 (41290-48775) | 0.98 (0.75-1.14) | 667462 (613563-732753) | 1231841 (1113585-1327064) | 0.85 (0.63-1.02) |
| Sex |  |  |  |  |  |  |  |  |  |
| Male | 160439 (148513-171372) | 562741 (502727-615420) | 2.51 (2.12-2.88) | 7596 (6939-8498) | 18635 (16822-20242) | 1.45 (1.07-1.78) | 229035 (208177-259563) | 509842 (461647-553336) | 1.23 (0.88-1.52) |
| Female | 478612 (434848-510132) | 1269019 (1123383-1393452) | 1.65 (1.37-1.96) | 15371 (14020-17250) | 26941 (23718-29329) | 0.75 (0.54-0.94) | 438427 (386614-489363) | 721999 (627829-792461) | 0.65 (0.43-0.88) |
| **SDI quintile** |  |  |  |  |  |  |  |  |  |
| High SDI | 258905 (251521-266861) | 558933 (507270-612715) | 1.16 (0.96-1.36) | 5861 (5475-6090) | 9376 (8005-10012) | 0.6 (0.44-0.68) | 139504 (132181-147230) | 204882 (183508-221176) | 0.47 (0.35-0.55) |
| High-middle SDI | 191304 (176475-201988) | 467744 (423256-516674) | 1.45 (1.2-1.76) | 6149 (5823-6452) | 9449 (8562-10154) | 0.54 (0.42-0.66) | 170356 (158241-180563) | 240969 (220217-262040) | 0.41 (0.31-0.54) |
| Low SDI | 21388 (14801-28946) | 80578 (65299-95847) | 2.77 (1.78-4.5) | 1859 (1495-2333) | 3881 (3179-4502) | 1.09 (0.66-1.6) | 65724 (49873-85336) | 130894 (107451-153143) | 0.99 (0.55-1.63) |
| Low-middle SDI | 52928 (42401-63115) | 214997 (182515-241862) | 3.06 (2.34-4) | 3559 (3092-4270) | 8873 (7802-9843) | 1.49 (1.05-1.87) | 119641 (102138-144707) | 268799 (234451-297879) | 1.25 (0.85-1.62) |
| Middle SDI | 114185 (100568-126068) | 508503 (443660-568712) | 3.45 (2.82-4.22) | 5524 (5102-6614) | 13968 (12460-15490) | 1.53 (1.08-1.86) | 171835 (158034-198063) | 385511 (341358-426264) | 1.24 (0.87-1.52) |
| **GBD region** |  |  |  |  |  |  |  |  |  |
| Andean Latin America | 2493 (2109-3082) | 15428 (11910-19625) | 5.19 (3.53-7.11) | 193 (170-232) | 624 (476-761) | 2.23 (1.29-3.08) | 5516 (4821-6634) | 15341 (11921-18813) | 1.78 (1.06-2.54) |
| Australasia | 4083 (3720-4478) | 14398 (11155-18454) | 2.53 (1.65-3.66) | 99 (93-105) | 236 (199-257) | 1.38 (0.97-1.61) | 2516 (2355-2726) | 5592 (4760-6245) | 1.22 (0.87-1.48) |
| Caribbean | 3363 (3083-3628) | 9238 (7693-11115) | 1.75 (1.25-2.31) | 153 (138-166) | 322 (269-373) | 1.11 (0.8-1.43) | 4334 (3846-4790) | 8612 (7132-10142) | 0.99 (0.68-1.33) |
| Central Asia | 5496 (4860-6365) | 11454 (10131-12975) | 1.08 (0.79-1.46) | 219 (193-256) | 309 (278-341) | 0.41 (0.21-0.62) | 6814 (5829-7875) | 9231 (8203-10321) | 0.35 (0.18-0.57) |
| Central Europe | 31688 (28916-33166) | 41636 (36005-48334) | 0.31 (0.13-0.59) | 1158 (1039-1195) | 1026 (900-1180) | -0.11 (-0.22-0.09) | 31064 (27105-32323) | 24290 (21221-28012) | -0.22 (-0.32-0) |
| Central Latin America | 11629 (10996-12182) | 53078 (44897-62689) | 3.56 (2.86-4.37) | 681 (632-708) | 1910 (1634-2187) | 1.8 (1.43-2.18) | 19122 (17830-19792) | 48382 (41446-56012) | 1.53 (1.19-1.91) |
| Central Sub-Saharan Africa | 870 (610-1179) | 2947 (2080-4148) | 2.39 (1.39-3.81) | 110 (81-148) | 242 (168-343) | 1.19 (0.66-1.79) | 3477 (2577-4594) | 7385 (5265-10251) | 1.12 (0.58-1.77) |
| East Asia | 77821 (64031-91818) | 330694 (274743-403528) | 3.25 (2.31-4.71) | 3504 (3037-4325) | 7621 (6346-8874) | 1.18 (0.64-1.68) | 109356 (93654-130567) | 197337 (165224-228530) | 0.8 (0.4-1.22) |
| Eastern Europe | 46269 (43328-52327) | 98832 (85611-114803) | 1.14 (0.81-1.48) | 1380 (1299-1518) | 1870 (1656-2088) | 0.36 (0.2-0.51) | 37214 (34767-41650) | 49426 (43997-55524) | 0.33 (0.17-0.48) |
| Eastern Sub-Saharan Africa | 11379 (7033-16770) | 37015 (28165-47702) | 2.25 (1.07-4.72) | 987 (719-1329) | 1795 (1403-2199) | 0.82 (0.33-1.52) | 36313 (24642-50651) | 62599 (49515-77614) | 0.72 (0.21-1.62) |
| High-income Asia Pacific | 53462 (50298-60051) | 123548 (105012-142979) | 1.87 (1.61-2.15) | 1194 (1110-1373) | 2754 (2128-3071) | 1.31 (0.76-1.56) | 28193 (26432-33054) | 49019 (41648-54668) | 0.85 (0.63-1.02) |
| High-income North America | 104713 (101134-107868) | 237369 (204321-275690) | 1.31 (0.79-1.74) | 1392 (1296-1446) | 2754 (2490-2887) | 0.98 (0.88-1.05) | 36534 (33992-39145) | 69048 (62830-74874) | 0.74 (0.31-0.91) |
| North Africa and Middle East | 29554 (24113-34046) | 163048 (130172-189792) | 1.27 (0.95-1.63) | 923 (753-1188) | 2290 (1981-2669) | 1.48 (1.09-2.04) | 30324 (25430-35353) | 74180 (62526-86119) | 0.89 (0.79-0.98) |
| Oceania | 343 (259-437) | 1114 (765-1522) | 4.52 (3.62-5.96) | 20 (16-27) | 50 (37-68) | 1.48 (0.97-2.1) | 645 (515-861) | 1576 (1156-2176) | 1.45 (1.05-1.99) |
| South Asia | 48650 (39844-60377) | 226467 (189126-263769) | 2.25 (1.49-3.33) | 3320 (2843-4214) | 9196 (7978-10477) | 1.77 (1.19-2.27) | 116556 (99247-149760) | 291575 (254403-330796) | 1.44 (0.94-2.1) |
| Southeast Asia | 48607 (37331-56449) | 194899 (153975-230803) | 3.65 (2.43-4.88) | 2441 (2098-2762) | 5862 (4999-6646) | 1.4 (1.05-1.79) | 74861 (62023-84632) | 164304 (136534-187956) | 1.5 (0.98-1.98) |
| Southern Latin America | 6303 (5725-6833) | 15087 (11622-19572) | 3.01 (2.29-3.92) | 340 (312-357) | 482 (443-518) | 0.42 (0.31-0.6) | 8727 (7877-9189) | 11411 (10559-12386) | 1.19 (0.85-1.58) |
| Southern Sub-Saharan Africa | 2274 (1921-2565) | 4909 (4160-5861) | 1.39 (0.85-2.17) | 124 (107-139) | 259 (219-293) | 1.08 (0.83-1.37) | 3878 (3392-4243) | 7497 (6317-8624) | 0.31 (0.2-0.5) |
| Tropical Latin America | 10746 (10199-11435) | 33189 (31029-36723) | 1.16 (0.8-1.7) | 598 (572-627) | 1237 (1133-1368) | 1.07 (0.95-1.23) | 17061 (16359-18030) | 31371 (29439-35742) | 0.93 (0.69-1.22) |
| Western Europe | 137458 (128720-143253) | 210571 (181812-241047) | 2.09 (1.87-2.39) | 3923 (3655-4054) | 4309 (3776-4614) | 0.1 (0.02-0.17) | 88850 (82749-92934) | 90233 (81225-98006) | 0.84 (0.74-1.01) |
| Western Sub-Saharan Africa | 1851 (1477-2212) | 6840 (5313-8600) | 0.53 (0.33-0.77) | 205 (159-239) | 429 (334-506) | 1.09 (0.75-1.48) | 6107 (4918-7124) | 13432 (10648-15987) | 0.02 (-0.05-0.09) |

percentage change from 1990 to 2019

Abbreviations: GBD=Global Burden of Disease. SDI=Socio-demographic Index. UI=uncertainty interval.

**Supplementary Table S2:**Prevalent cases, deaths and disability adjusted life years (DALYs) for Thyroid cancer (TC) by 204 countries or territories in 2019 and 2019, and their percentage change from 1990 to 2019

|  | **Prevalent cases** | | | **Deaths** | | | **DALYs** | | |
| --- | --- | --- | --- | --- | --- | --- | --- | --- | --- |
| **Characteristics** | **1990no**  **(95%UI)** | **2019no**  **(95%UI)** | **Percentage**  **change**  **(95%UI)** | **1990no**  **(95%UI)** | **2019no**  **(95%UI)** | **Percentage**  **change**  **(95%UI)** | **1990no**  **(95%UI)** | **2019no**  **(95%UI)** | **Percentage**  **change**  **(95%UI)** |
| Afghanistan | 681 (293-1184) | 3253 (1487-5607) | 3.78 (2.22-6.43) | 56 (34-84) | 118 (75-173) | 1.1 (0.48-1.93) | 1769 (966-2774) | 4229 (2421-6523) | 1.39 (0.67-2.46) |
| Albania | 323 (244-367) | 764 (551-1034) | 1.36 (0.64-2.39) | 13 (11-14) | 18 (14-25) | 0.45 (0.06-0.98) | 389 (334-424) | 475 (353-635) | 0.22 (-0.12-0.7) |
| Algeria | 3076 (2208-4110) | 15799 (10133-22012) | 4.14 (2.24-6.92) | 61 (48-85) | 172 (136-214) | 1.84 (0.97-2.91) | 2196 (1718-2810) | 5795 (4300-7459) | 1.64 (0.78-2.74) |
| American Samoa | 4 (3-6) | 13 (9-17) | 2.04 (0.98-3.43) | 0 (0-0) | 1 (0-1) | 1.61 (0.89-2.5) | 5 (4-7) | 13 (9-16) | 1.41 (0.72-2.26) |
| Andorra | 15 (11-22) | 49 (35-66) | 2.17 (1-3.76) | 0 (0-0) | 1 (1-1) | 1.4 (0.6-2.47) | 9 (7-13) | 19 (14-26) | 1.15 (0.4-2.12) |
| Angola | 141 (96-203) | 751 (494-1125) | 4.32 (2.19-7.39) | 19 (13-26) | 52 (36-77) | 1.78 (0.93-2.97) | 618 (440-855) | 1651 (1162-2382) | 1.67 (0.8-2.85) |
| Antigua and Barbuda | 7 (6-8) | 26 (21-33) | 2.64 (1.78-3.73) | 0 (0-0) | 1 (1-1) | 1.2 (0.8-1.66) | 8 (8-9) | 20 (17-24) | 1.35 (0.92-1.86) |
| Argentina | 4541 (3952-5054) | 8601 (6509-11321) | 0.89 (0.4-1.67) | 250 (223-266) | 292 (267-322) | 0.17 (0.05-0.39) | 6472 (5627-6899) | 7161 (6539-7932) | 0.11 (0-0.33) |
| Armenia | 162 (141-200) | 658 (531-800) | 3.05 (2.13-4.26) | 8 (7-10) | 25 (21-30) | 2.35 (1.38-3.15) | 210 (187-283) | 578 (479-688) | 1.75 (0.93-2.42) |
| Australia | 3710 (3359-4104) | 13290 (10077-17465) | 2.58 (1.63-3.79) | 79 (73-85) | 187 (157-205) | 1.37 (1.03-1.63) | 2027 (1879-2205) | 4489 (3841-5125) | 1.22 (0.89-1.51) |
| Austria | 4046 (3481-4527) | 4956 (3866-6284) | 0.22 (-0.07-0.6) | 101 (89-108) | 88 (75-98) | -0.13 (-0.22-0.01) | 2252 (1987-2445) | 1884 (1643-2140) | -0.16 (-0.26--0.02) |
| Azerbaijan | 500 (405-608) | 1531 (1175-1979) | 2.06 (1.29-3.1) | 22 (19-29) | 41 (33-55) | 0.88 (0.39-1.4) | 721 (612-873) | 1300 (1033-1661) | 0.8 (0.38-1.29) |
| Bahamas | 30 (26-34) | 86 (67-111) | 1.92 (1.2-2.9) | 1 (1-1) | 2 (2-3) | 1.33 (0.88-1.91) | 33 (30-37) | 75 (60-93) | 1.25 (0.79-1.88) |
| Bahrain | 37 (29-50) | 387 (291-499) | 9.59 (5.96-14.3) | 1 (1-1) | 5 (3-6) | 3.06 (1.88-4.57) | 33 (27-42) | 142 (109-183) | 3.27 (2-4.89) |
| Bangladesh | 4152 (2786-5949) | 17036 (11359-25991) | 3.1 (1.29-6.77) | 301 (229-390) | 646 (472-888) | 1.15 (0.47-2.26) | 10898 (7875-14508) | 19906 (14232-28069) | 0.83 (0.19-1.98) |
| Barbados | 34 (30-38) | 91 (72-114) | 1.69 (1.04-2.49) | 2 (2-2) | 3 (3-4) | 0.71 (0.4-1.07) | 44 (40-48) | 77 (64-93) | 0.76 (0.42-1.17) |
| Belarus | 2182 (1897-2548) | 4826 (3544-6511) | 1.21 (0.59-2.02) | 61 (55-70) | 96 (76-122) | 0.58 (0.23-1.03) | 1685 (1517-1930) | 2423 (1852-3152) | 0.44 (0.09-0.88) |
| Belgium | 3396 (2933-3812) | 4632 (3462-5975) | 0.36 (0-0.85) | 153 (138-167) | 112 (99-126) | -0.27 (-0.35--0.15) | 3060 (2751-3299) | 2331 (2062-2635) | -0.24 (-0.33--0.11) |
| Belize | 5 (4-5) | 34 (28-40) | 6.21 (4.7-8.07) | 0 (0-0) | 1 (1-1) | 2.69 (2.1-3.39) | 8 (7-8) | 32 (28-37) | 3.25 (2.55-4.1) |
| Benin | 57 (34-79) | 209 (135-303) | 2.66 (1.28-4.79) | 6 (4-7) | 14 (10-18) | 1.25 (0.66-2.09) | 183 (119-229) | 436 (309-597) | 1.39 (0.65-2.48) |
| Bermuda | 15 (13-18) | 32 (25-41) | 1.1 (0.6-1.74) | 1 (1-1) | 1 (1-1) | 0.39 (0.11-0.75) | 15 (14-17) | 18 (15-23) | 0.22 (-0.03-0.53) |
| Bhutan | 22 (12-35) | 104 (65-159) | 3.65 (1.42-8.48) | 2 (1-2) | 4 (3-5) | 1.4 (0.63-2.75) | 59 (37-86) | 114 (80-164) | 0.94 (0.21-2.33) |
| Bolivia (Plurinational State of) | 389 (255-556) | 2269 (1558-3166) | 4.83 (2.82-8.24) | 46 (34-60) | 143 (106-184) | 2.11 (1.28-3.41) | 1305 (946-1726) | 3547 (2608-4626) | 1.72 (0.96-2.92) |
| Bosnia and Herzegovina | 496 (332-579) | 844 (576-1123) | 0.7 (0.21-1.31) | 20 (14-23) | 23 (18-30) | 0.14 (-0.12-0.52) | 576 (403-644) | 559 (416-715) | -0.03 (-0.26-0.29) |
| Botswana | 9 (6-12) | 48 (31-68) | 4.61 (2.44-7.77) | 1 (0-1) | 1 (1-2) | 1.83 (0.85-3.03) | 18 (14-25) | 52 (36-72) | 1.86 (0.9-3.12) |
| Brazil | 10530 (9992-11198) | 31955 (29876-35619) | 2.03 (1.81-2.34) | 585 (560-612) | 1191 (1088-1334) | 1.04 (0.92-1.21) | 16695 (16003-17644) | 30147 (28236-34681) | 0.81 (0.7-1) |
| Brunei Darussalam | 41 (29-55) | 165 (129-208) | 3.08 (1.91-5.03) | 1 (1-1) | 3 (2-3) | 1.77 (1.15-2.63) | 30 (24-37) | 81 (66-96) | 1.7 (1.08-2.57) |
| Bulgaria | 1526 (1317-1725) | 1778 (1328-2297) | 0.17 (-0.15-0.59) | 68 (62-74) | 62 (49-78) | -0.09 (-0.29-0.18) | 1896 (1700-2076) | 1565 (1207-1972) | -0.17 (-0.36-0.07) |
| Burkina Faso | 119 (72-165) | 406 (268-562) | 2.42 (1.22-4.16) | 13 (8-17) | 26 (18-33) | 0.99 (0.51-1.58) | 397 (257-506) | 831 (587-1076) | 1.09 (0.54-1.83) |
| Burundi | 227 (124-368) | 686 (464-970) | 2.02 (0.67-5.3) | 20 (14-29) | 37 (27-50) | 0.85 (0.15-2.08) | 718 (451-1090) | 1375 (966-1901) | 0.92 (0.14-2.4) |
| Cabo Verde | 6 (5-7) | 35 (25-47) | 5.35 (3.33-7.89) | 1 (0-1) | 1 (1-2) | 1.28 (0.59-2.19) | 14 (11-16) | 36 (27-47) | 1.6 (0.9-2.47) |
| Cambodia | 678 (355-985) | 3474 (2075-4924) | 4.12 (2.44-6.88) | 56 (37-72) | 150 (100-192) | 1.68 (1.01-2.63) | 1765 (1087-2338) | 4174 (2685-5483) | 1.37 (0.71-2.28) |
| Cameroon | 101 (74-137) | 476 (299-718) | 3.71 (1.81-6.55) | 7 (6-9) | 19 (14-27) | 1.59 (0.86-2.62) | 251 (199-313) | 703 (479-990) | 1.8 (0.87-3.07) |
| Canada | 7639 (6831-8466) | 16642 (12578-21540) | 1.18 (0.61-1.9) | 140 (130-150) | 288 (250-317) | 1.06 (0.81-1.28) | 3592 (3333-3887) | 6544 (5736-7354) | 0.82 (0.62-1.03) |
| Central African Republic | 46 (30-64) | 87 (57-128) | 0.87 (0.28-1.8) | 7 (5-9) | 12 (8-17) | 0.68 (0.21-1.31) | 221 (162-297) | 374 (253-546) | 0.69 (0.19-1.37) |
| Chad | 58 (34-84) | 171 (111-233) | 1.93 (0.91-3.44) | 8 (5-10) | 15 (10-19) | 0.94 (0.5-1.6) | 220 (136-288) | 474 (331-620) | 1.15 (0.59-1.99) |
| Chile | 1462 (1289-1695) | 5648 (4252-7407) | 2.86 (1.79-4.25) | 74 (67-80) | 160 (141-178) | 1.17 (0.88-1.47) | 1865 (1703-2041) | 3571 (3187-3982) | 0.91 (0.67-1.21) |
| China | 69239 (55771-82950) | 310328 (255041-382138) | 3.48 (2.43-5.12) | 3319 (2862-4133) | 7239 (6012-8476) | 1.18 (0.63-1.72) | 103493 (87958-124715) | 187319 (156236-219112) | 0.81 (0.4-1.25) |
| Colombia | 3578 (3206-4051) | 12281 (9014-16716) | 2.43 (1.47-3.68) | 186 (175-198) | 396 (307-498) | 1.13 (0.65-1.69) | 5342 (5006-5694) | 9427 (7234-12047) | 0.76 (0.35-1.27) |
| Comoros | 14 (4-23) | 64 (37-99) | 3.52 (1.44-17.47) | 1 (1-2) | 4 (3-5) | 1.72 (0.79-4.54) | 45 (18-67) | 116 (78-161) | 1.59 (0.58-5.76) |
| Congo | 54 (37-76) | 195 (129-276) | 2.6 (1.24-4.62) | 7 (5-9) | 14 (10-18) | 0.96 (0.41-1.73) | 210 (158-271) | 410 (295-560) | 0.95 (0.34-1.81) |
| Cook Islands | 3 (2-4) | 4 (3-6) | 0.49 (-0.01-1.18) | 0 (0-0) | 0 (0-0) | 0.26 (-0.05-0.68) | 3 (2-4) | 3 (2-4) | 0.08 (-0.21-0.47) |
| Costa Rica | 475 (412-541) | 1479 (1089-1985) | 2.11 (1.24-3.31) | 13 (12-14) | 34 (26-44) | 1.55 (0.97-2.27) | 391 (357-424) | 891 (676-1163) | 1.28 (0.75-1.99) |
| C么te d'Ivoire | 157 (98-214) | 442 (280-641) | -0.05 (-0.33-0.33) | 13 (8-16) | 29 (20-38) | -0.23 (-0.41-0.03) | 439 (291-570) | 954 (651-1289) | -0.33 (-0.5--0.08) |
| Croatia | 2272 (1931-2620) | 2153 (1585-2835) | 1.58 (0.97-2.43) | 51 (44-56) | 39 (31-50) | 1.25 (0.8-1.78) | 1365 (1154-1516) | 908 (699-1168) | 0.99 (0.57-1.51) |
| Cuba | 1523 (1360-1719) | 3931 (3069-5056) | 1.94 (1.24-3.36) | 48 (44-52) | 108 (87-133) | 0.42 (0.13-0.93) | 1323 (1221-1440) | 2632 (2093-3291) | 0.38 (0.1-0.87) |
| Cyprus | 154 (107-188) | 452 (365-562) | 0.22 (-0.08-0.64) | 6 (5-8) | 9 (7-11) | -0.22 (-0.37-0) | 153 (113-179) | 211 (173-252) | -0.26 (-0.42--0.04) |
| Czechia | 4501 (4048-5015) | 5512 (4219-7013) | 1.82 (0.72-3.31) | 115 (104-123) | 90 (73-110) | 1.29 (0.66-2.06) | 2827 (2569-3035) | 2082 (1650-2577) | 1.17 (0.49-2.04) |
| Democratic People's Republic of Korea | 3887 (2391-5684) | 5900 (4056-8544) | 0.52 (-0.04-1.44) | 93 (69-122) | 165 (130-206) | 0.78 (0.32-1.4) | 3114 (2145-4263) | 4718 (3560-6067) | 0.52 (0.08-1.16) |
| Democratic Republic of the Congo | 594 (399-829) | 1780 (1206-2588) | 2 (1.02-3.4) | 74 (52-104) | 157 (103-230) | 1.13 (0.55-1.85) | 2303 (1632-3233) | 4721 (3201-6717) | 1.05 (0.47-1.76) |
| Denmark | 733 (652-844) | 1880 (1409-2461) | 1.57 (0.79-2.43) | 27 (25-31) | 49 (42-56) | 0.82 (0.49-1.09) | 620 (567-735) | 1039 (898-1178) | 0.68 (0.35-0.92) |
| Djibouti | 15 (9-22) | 109 (60-181) | 6.4 (3.14-12.05) | 1 (1-1) | 5 (3-7) | 4.24 (2.44-6.97) | 34 (23-48) | 164 (101-251) | 3.76 (1.94-6.66) |
| Dominica | 7 (5-8) | 10 (8-13) | 0.58 (0.16-1.12) | 0 (0-1) | 1 (0-1) | 0.34 (0.06-0.69) | 11 (9-12) | 15 (12-19) | 0.36 (0.07-0.75) |
| Dominican Republic | 319 (243-388) | 1718 (1072-2470) | 4.39 (2.44-6.75) | 19 (14-22) | 66 (45-89) | 2.46 (1.42-3.7) | 581 (438-678) | 1873 (1228-2606) | 2.22 (1.17-3.46) |
| Ecuador | 578 (475-935) | 5286 (3904-7195) | 8.15 (4.18-12.16) | 42 (36-67) | 217 (155-282) | 4.15 (1.53-6.17) | 1181 (1021-1876) | 5186 (3861-6720) | 3.39 (1.37-5.1) |
| Egypt | 4473 (3339-5660) | 16801 (11711-22940) | 2.76 (1.44-4.6) | 130 (111-190) | 271 (186-435) | 1.08 (0.47-1.76) | 5236 (4424-6342) | 9697 (6858-13444) | 0.85 (0.28-1.59) |
| El Salvador | 471 (251-563) | 912 (648-1270) | 0.94 (0.27-3.31) | 34 (17-39) | 35 (26-46) | 0.03 (-0.27-1.16) | 941 (507-1080) | 855 (639-1148) | -0.09 (-0.37-0.91) |
| Equatorial Guinea | 6 (4-8) | 45 (26-75) | 7.03 (3.36-14.49) | 1 (1-1) | 2 (2-3) | 1.29 (0.45-2.6) | 31 (21-42) | 69 (44-108) | 1.23 (0.33-2.74) |
| Eritrea | 70 (43-111) | 464 (292-703) | 5.62 (2.64-11.52) | 7 (5-11) | 27 (18-38) | 2.69 (1.32-4.72) | 280 (196-412) | 978 (650-1391) | 2.5 (1.12-4.65) |
| Estonia | 464 (404-533) | 688 (521-912) | 0.48 (0.07-1.02) | 14 (12-15) | 17 (13-22) | 0.28 (-0.04-0.63) | 356 (325-391) | 352 (271-453) | -0.01 (-0.25-0.27) |
| Eswatini | 18 (13-25) | 52 (30-80) | 1.86 (0.63-3.68) | 1 (1-2) | 3 (2-5) | 1.3 (0.57-2.31) | 47 (34-59) | 106 (69-150) | 1.28 (0.49-2.42) |
| Ethiopia | 6891 (3010-11735) | 17282 (12079-24370) | 1.51 (0.38-5.15) | 647 (394-942) | 848 (631-1090) | 0.31 (-0.13-1.11) | 24075 (13246-36968) | 28680 (21693-37284) | 0.19 (-0.25-1.16) |
| Fiji | 116 (63-155) | 226 (121-317) | 0.96 (0.27-1.98) | 5 (3-7) | 9 (5-12) | 0.74 (0.2-1.48) | 169 (97-219) | 266 (157-358) | 0.57 (0.08-1.24) |
| Finland | 1511 (1351-1680) | 2885 (2191-3755) | 0.91 (0.41-1.56) | 49 (45-53) | 68 (58-76) | 0.37 (0.16-0.57) | 1118 (1032-1207) | 1361 (1166-1541) | 0.22 (0.03-0.4) |
| France | 16750 (15079-18387) | 25534 (19043-33503) | 0.52 (0.12-1.03) | 637 (579-679) | 604 (513-685) | -0.05 (-0.16-0.08) | 13640 (12676-14467) | 12286 (10654-13942) | -0.1 (-0.21-0.04) |
| Gabon | 28 (21-37) | 89 (61-126) | 2.13 (1.02-3.67) | 3 (3-4) | 6 (4-7) | 0.7 (0.23-1.21) | 93 (72-117) | 160 (117-212) | 0.72 (0.2-1.31) |
| Gambia | 12 (8-16) | 50 (34-71) | 3.33 (1.66-5.95) | 1 (1-1) | 3 (2-4) | 2.28 (1.27-3.65) | 29 (21-39) | 92 (68-120) | 2.18 (1.11-3.66) |
| Georgia | 592 (480-710) | 906 (702-1115) | 0.53 (0.1-1.11) | 22 (18-25) | 32 (25-38) | 0.47 (0.15-0.88) | 607 (509-701) | 825 (635-991) | 0.36 (0.04-0.76) |
| Germany | 31611 (28284-34744) | 42913 (31964-57253) | 0.36 (0-0.81) | 1046 (944-1106) | 992 (870-1094) | -0.05 (-0.14-0.05) | 23544 (21051-24967) | 20704 (18328-23145) | -0.12 (-0.21--0.02) |
| Ghana | 105 (76-138) | 410 (290-543) | 2.92 (1.58-4.78) | 11 (7-14) | 30 (21-39) | 1.76 (0.98-2.89) | 350 (247-457) | 914 (680-1193) | 1.61 (0.82-2.68) |
| Greece | 2302 (2060-2551) | 3466 (2628-4548) | 0.51 (0.11-1) | 86 (78-93) | 105 (91-115) | 0.21 (0.08-0.36) | 1781 (1641-1915) | 1952 (1736-2159) | 0.1 (-0.02-0.22) |
| Greenland | 7 (5-8) | 11 (8-15) | 0.7 (0.2-1.44) | 0 (0-0) | 0 (0-1) | 1.03 (0.24-1.73) | 7 (5-8) | 10 (7-13) | 0.56 (0.1-1.08) |
| Grenada | 12 (10-14) | 24 (20-30) | 1.04 (0.58-1.65) | 1 (1-1) | 1 (1-1) | 0.17 (-0.01-0.41) | 22 (20-25) | 28 (24-33) | 0.27 (0.06-0.54) |
| Guam | 10 (8-15) | 46 (31-58) | 3.57 (1.18-5.49) | 0 (0-0) | 1 (1-2) | 3.71 (1.18-5.42) | 8 (7-12) | 37 (23-46) | 3.32 (0.93-4.93) |
| Guatemala | 284 (240-335) | 1582 (1191-2052) | 4.57 (3.04-6.47) | 27 (23-30) | 74 (58-92) | 1.78 (1.17-2.57) | 809 (711-919) | 2048 (1588-2588) | 1.53 (0.95-2.33) |
| Guinea | 117 (89-151) | 356 (248-502) | 2.04 (0.98-3.56) | 14 (11-17) | 25 (19-33) | 0.86 (0.38-1.43) | 425 (352-518) | 859 (636-1123) | 1.02 (0.46-1.73) |
| Guinea-Bissau | 15 (9-23) | 37 (23-51) | 1.37 (0.53-2.57) | 2 (1-2) | 3 (2-3) | 0.5 (0.1-1.02) | 57 (36-78) | 88 (60-116) | 0.54 (0.08-1.11) |
| Guyana | 41 (33-49) | 102 (74-137) | 1.5 (0.75-2.54) | 3 (2-3) | 5 (3-6) | 0.69 (0.26-1.25) | 83 (70-96) | 141 (107-183) | 0.7 (0.25-1.28) |
| Haiti | 269 (135-425) | 822 (473-1274) | 2.06 (1.01-3.92) | 32 (20-43) | 57 (36-84) | 0.8 (0.31-1.48) | 1013 (600-1402) | 1799 (1117-2657) | 0.78 (0.29-1.52) |
| Honduras | 489 (187-647) | 3512 (953-5395) | 6.18 (3.42-9.79) | 39 (15-49) | 185 (56-257) | 3.76 (2.46-5.12) | 1163 (474-1468) | 4918 (1523-7091) | 3.23 (1.94-4.66) |
| Hungary | 3183 (2802-3544) | 3829 (2972-4853) | 0.2 (-0.1-0.6) | 129 (116-137) | 99 (81-121) | -0.23 (-0.38--0.03) | 3179 (2845-3404) | 2272 (1837-2789) | -0.29 (-0.43--0.09) |
| Iceland | 175 (156-198) | 317 (263-374) | 0.81 (0.46-1.24) | 4 (4-4) | 6 (5-6) | 0.41 (0.23-0.64) | 97 (89-107) | 129 (112-147) | 0.33 (0.15-0.53) |
| India | 37011 (29507-47145) | 169543 (136587-205896) | 3.58 (2.27-4.88) | 2461 (2068-3250) | 7075 (5979-8315) | 1.87 (1.18-2.5) | 86623 (71999-114315) | 217465 (181112-254846) | 1.51 (0.93-2.05) |
| Indonesia | 13453 (8780-17012) | 47136 (31955-60457) | 2.5 (1.59-3.76) | 815 (606-954) | 1998 (1459-2389) | 1.45 (0.94-2.02) | 25472 (18284-30161) | 55359 (40142-67329) | 1.17 (0.71-1.72) |
| Iran (Islamic Republic of) | 4317 (3532-5222) | 27337 (16709-31333) | 5.33 (3.03-7.37) | 99 (86-118) | 341 (245-375) | 2.44 (1.39-3.28) | 3172 (2747-3697) | 10469 (6928-11742) | 2.3 (1.1-3.11) |
| Iraq | 1448 (1058-1940) | 10904 (7428-15294) | 6.53 (3.59-11.09) | 43 (33-61) | 159 (120-201) | 2.74 (1.39-4.42) | 1400 (1081-1860) | 5290 (3807-6979) | 2.78 (1.39-4.58) |
| Ireland | 710 (625-795) | 1929 (1416-2548) | 1.72 (0.98-2.71) | 25 (22-27) | 31 (26-35) | 0.25 (0.09-0.47) | 570 (515-616) | 728 (611-854) | 0.28 (0.1-0.51) |
| Israel | 833 (740-940) | 3436 (2567-4494) | 3.13 (2.03-4.51) | 38 (35-42) | 86 (74-97) | 1.24 (0.92-1.54) | 861 (791-940) | 1826 (1597-2064) | 1.12 (0.84-1.42) |
| Italy | 41655 (37775-45257) | 52610 (41811-65266) | 0.26 (0-0.59) | 646 (605-668) | 710 (608-772) | 0.1 (-0.01-0.19) | 16531 (15025-17604) | 15601 (13573-17413) | -0.06 (-0.14-0.04) |
| Jamaica | 143 (125-165) | 639 (471-851) | 3.47 (2.15-5.16) | 7 (7-8) | 20 (16-25) | 1.73 (1.1-2.52) | 185 (169-202) | 528 (407-663) | 1.86 (1.15-2.73) |
| Japan | 47159 (44270-50207) | 76771 (63058-92419) | 0.63 (0.34-0.97) | 1037 (955-1076) | 2139 (1611-2403) | 1.06 (0.67-1.27) | 23732 (22332-25106) | 34347 (28947-38324) | 0.45 (0.26-0.57) |
| Jordan | 458 (340-604) | 2750 (2123-3579) | 5.01 (3.04-8.45) | 11 (9-13) | 34 (28-43) | 2.25 (1.46-3.34) | 356 (287-437) | 1084 (877-1371) | 2.05 (1.29-3.18) |
| Kazakhstan | 3301 (2766-4143) | 4690 (3854-5711) | 0.42 (0.11-0.89) | 116 (93-139) | 99 (83-116) | -0.15 (-0.31-0.16) | 3759 (2976-4586) | 3031 (2540-3620) | -0.19 (-0.35-0.13) |
| Kenya | 329 (247-479) | 1751 (1245-2536) | 4.32 (2.83-6.41) | 18 (15-25) | 71 (54-97) | 2.87 (1.92-3.8) | 653 (517-895) | 2519 (1865-3477) | 2.86 (1.87-3.9) |
| Kiribati | 1 (1-2) | 3 (2-5) | 1.37 (0.68-2.31) | 0 (0-0) | 0 (0-0) | 0.58 (0.17-1.09) | 4 (3-6) | 7 (5-9) | 0.59 (0.19-1.11) |
| Kuwait | 316 (271-368) | 1306 (1037-1644) | 3.14 (2.13-4.42) | 4 (3-4) | 12 (10-14) | 2.27 (1.7-3.02) | 129 (114-145) | 372 (303-459) | 1.89 (1.35-2.56) |
| Kyrgyzstan | 351 (291-414) | 654 (526-798) | 0.86 (0.41-1.47) | 16 (14-18) | 18 (15-21) | 0.12 (-0.06-0.34) | 474 (420-532) | 537 (454-637) | 0.13 (-0.06-0.38) |
| Lao People's Democratic Republic | 264 (127-420) | 952 (559-1416) | 2.61 (1.18-5.3) | 28 (17-39) | 47 (31-61) | 0.68 (0.17-1.34) | 871 (515-1280) | 1367 (882-1838) | 0.57 (0.05-1.32) |
| Latvia | 597 (531-675) | 850 (637-1115) | 0.42 (0.05-0.92) | 18 (17-20) | 26 (20-32) | 0.39 (0.05-0.76) | 494 (451-545) | 554 (439-689) | 0.12 (-0.14-0.42) |
| Lebanon | 690 (490-925) | 3727 (2593-5084) | 4.4 (2.59-7.36) | 15 (12-21) | 35 (28-46) | 1.28 (0.73-2.28) | 480 (375-603) | 1070 (817-1377) | 1.23 (0.64-2.2) |
| Lesotho | 32 (22-44) | 87 (49-133) | 1.69 (0.52-3.62) | 4 (3-5) | 8 (5-11) | 1.02 (0.31-1.94) | 106 (76-138) | 225 (144-315) | 1.13 (0.35-2.2) |
| Liberia | 25 (14-34) | 93 (58-134) | 2.72 (1.45-4.72) | 3 (2-4) | 6 (4-7) | 0.65 (0.22-1.21) | 96 (62-125) | 179 (120-244) | 0.86 (0.31-1.62) |
| Libya | 479 (320-656) | 2218 (1440-3176) | 3.63 (1.89-6.62) | 9 (7-12) | 25 (18-33) | 1.64 (0.93-2.81) | 315 (234-408) | 857 (614-1166) | 1.72 (0.89-3.1) |
| Lithuania | 1079 (943-1228) | 1174 (885-1512) | 0.09 (-0.18-0.43) | 23 (21-25) | 27 (22-33) | 0.17 (-0.05-0.45) | 659 (596-720) | 633 (500-790) | -0.04 (-0.23-0.2) |
| Luxembourg | 158 (140-175) | 301 (239-382) | 0.91 (0.5-1.53) | 6 (5-6) | 6 (5-7) | 0.05 (-0.11-0.26) | 129 (118-140) | 134 (114-163) | 0.04 (-0.12-0.29) |
| Madagascar | 513 (361-733) | 1623 (1100-2309) | 2.16 (0.99-4.24) | 32 (27-40) | 79 (58-108) | 1.46 (0.71-2.41) | 1263 (1008-1639) | 2992 (2153-4108) | 1.37 (0.61-2.52) |
| Malawi | 451 (317-642) | 1239 (723-2011) | 1.75 (0.51-3.79) | 28 (23-35) | 56 (39-77) | 0.96 (0.36-1.77) | 1069 (809-1374) | 2032 (1309-3058) | 0.9 (0.2-1.89) |
| Malaysia | 2364 (1763-2978) | 11091 (8112-14685) | 3.69 (2.15-5.83) | 88 (76-103) | 234 (180-304) | 1.67 (0.98-2.5) | 2666 (2240-3102) | 6583 (5087-8478) | 1.47 (0.79-2.3) |
| Maldives | 15 (9-22) | 103 (80-135) | 5.72 (3.27-10.85) | 1 (1-1) | 2 (2-3) | 1.24 (0.56-2.53) | 31 (20-40) | 60 (49-74) | 0.98 (0.35-2.2) |
| Mali | 170 (131-220) | 609 (390-903) | 2.58 (1.26-4.56) | 15 (13-19) | 31 (23-41) | 1 (0.48-1.68) | 523 (430-639) | 1105 (782-1534) | 1.12 (0.45-1.99) |
| Malta | 97 (86-113) | 206 (167-253) | 1.12 (0.64-1.66) | 3 (3-4) | 5 (4-6) | 0.41 (0.19-0.66) | 79 (72-86) | 102 (87-119) | 0.3 (0.09-0.53) |
| Marshall Islands | 2 (2-3) | 7 (5-11) | 2.48 (1.34-4.24) | 0 (0-0) | 0 (0-0) | 1.16 (0.56-1.92) | 4 (4-6) | 10 (7-14) | 1.32 (0.67-2.2) |
| Mauritania | 31 (19-41) | 87 (55-129) | 1.82 (0.68-3.85) | 4 (2-5) | 5 (4-7) | 0.43 (0.03-1.03) | 104 (69-133) | 145 (98-200) | 0.4 (-0.07-1.13) |
| Mauritius | 97 (86-109) | 257 (196-326) | 1.66 (0.98-2.43) | 3 (3-3) | 6 (5-8) | 1 (0.61-1.49) | 91 (84-99) | 168 (134-206) | 0.84 (0.46-1.3) |
| Mexico | 5113 (4948-5358) | 26035 (21718-30915) | 4.09 (3.23-5.07) | 319 (304-329) | 944 (811-1089) | 1.96 (1.54-2.4) | 8676 (8387-8982) | 23996 (20734-27662) | 1.77 (1.37-2.18) |
| Micronesia (Federated States of) | 6 (4-9) | 17 (10-25) | 1.78 (0.61-3.25) | 0 (0-1) | 1 (0-1) | 0.54 (0.03-1.2) | 14 (11-19) | 22 (15-30) | 0.55 (0.02-1.24) |
| Monaco | 18 (13-23) | 31 (23-41) | 0.77 (0.2-1.54) | 1 (0-1) | 1 (1-1) | 0.26 (-0.04-0.65) | 13 (10-16) | 16 (12-20) | 0.27 (-0.08-0.72) |
| Mongolia | 48 (36-61) | 281 (166-388) | 4.89 (3-7.59) | 5 (3-7) | 13 (5-17) | 1.49 (0.78-2.41) | 147 (86-182) | 390 (184-526) | 1.64 (0.89-2.66) |
| Montenegro | 172 (145-206) | 303 (245-379) | 0.76 (0.35-1.31) | 4 (4-5) | 6 (5-8) | 0.53 (0.19-0.91) | 119 (103-137) | 166 (136-200) | 0.39 (0.08-0.76) |
| Morocco | 2924 (1872-3891) | 12012 (7916-17710) | 3.11 (1.57-5.58) | 99 (76-122) | 226 (168-301) | 1.29 (0.65-2.04) | 3341 (2629-4046) | 7099 (5234-9473) | 1.12 (0.47-1.93) |
| Mozambique | 392 (271-557) | 1953 (1185-3001) | 3.98 (1.94-7.12) | 40 (31-52) | 112 (78-155) | 1.81 (0.88-3) | 1377 (1028-1838) | 3967 (2648-5654) | 1.88 (0.87-3.21) |
| Myanmar | 3344 (1730-5155) | 9942 (6626-13643) | 1.97 (0.79-4.35) | 263 (173-359) | 456 (342-565) | 0.74 (0.24-1.44) | 8307 (5019-11658) | 12605 (9186-16043) | 0.52 (0.04-1.25) |
| Namibia | 34 (21-48) | 162 (101-252) | 3.79 (1.72-7.47) | 3 (2-4) | 7 (5-9) | 1.34 (0.64-2.34) | 89 (65-115) | 209 (145-291) | 1.34 (0.53-2.56) |
| Nauru | 1 (1-2) | 2 (1-3) | 0.93 (0.39-1.73) | 0 (0-0) | 0 (0-0) | 0.22 (-0.08-0.61) | 1 (1-2) | 2 (1-2) | 0.32 (-0.01-0.74) |
| Nepal | 775 (491-1190) | 3423 (2327-4897) | 3.41 (1.6-6.69) | 57 (43-76) | 154 (117-207) | 1.72 (0.94-2.74) | 2068 (1482-2834) | 4680 (3430-6331) | 1.26 (0.51-2.31) |
| Netherlands | 3603 (3224-3991) | 8071 (5955-10543) | 1.24 (0.67-1.98) | 101 (91-108) | 161 (135-180) | 0.6 (0.39-0.8) | 2306 (2096-2473) | 3453 (2883-3916) | 0.5 (0.31-0.69) |
| New Zealand | 373 (328-421) | 1107 (848-1411) | 1.97 (1.2-2.85) | 20 (18-23) | 49 (41-54) | 1.42 (0.78-1.77) | 489 (450-560) | 1103 (913-1215) | 1.25 (0.64-1.56) |
| Nicaragua | 116 (96-152) | 887 (658-1151) | 6.62 (4.27-9.43) | 7 (6-8) | 32 (26-38) | 3.85 (2.5-5.12) | 198 (173-249) | 813 (632-1003) | 3.1 (1.95-4.28) |
| Niger | 14 (11-19) | 72 (47-105) | 3.98 (2.31-6.14) | 1 (1-2) | 4 (3-5) | 1.89 (1.16-2.91) | 53 (42-68) | 170 (117-241) | 2.18 (1.27-3.46) |
| Nigeria | 669 (481-908) | 2786 (1901-4034) | 3.17 (1.72-5.14) | 88 (65-109) | 180 (134-224) | 1.04 (0.55-1.73) | 2384 (1858-2974) | 5218 (3918-6704) | 1.19 (0.62-2.01) |
| Niue | 0 (0-0) | 1 (0-1) | 0.7 (0.1-1.61) | 0 (0-0) | 0 (0-0) | -0.06 (-0.32-0.24) | 0 (0-1) | 0 (0-1) | 0.01 (-0.27-0.39) |
| North Macedonia | 275 (162-324) | 579 (409-788) | 1.1 (0.5-2.01) | 10 (8-12) | 13 (10-17) | 0.27 (-0.03-0.77) | 308 (207-346) | 357 (269-457) | 0.16 (-0.12-0.66) |
| Northern Mariana Islands | 5 (3-7) | 17 (12-23) | 2.69 (0.86-4.68) | 0 (0-0) | 0 (0-0) | 3.78 (1.39-5.49) | 3 (2-4) | 11 (8-14) | 2.98 (0.89-4.67) |
| Norway | 1148 (1050-1267) | 2408 (1981-2897) | 1.1 (0.68-1.61) | 38 (35-40) | 49 (43-53) | 0.29 (0.18-0.4) | 813 (772-864) | 1013 (911-1122) | 0.25 (0.13-0.37) |
| Oman | 133 (94-181) | 984 (721-1300) | 6.4 (3.9-10.25) | 3 (2-4) | 7 (6-9) | 1.71 (0.89-2.91) | 93 (69-125) | 284 (216-359) | 2.07 (1.11-3.44) |
| Pakistan | 6689 (5394-8285) | 36361 (25890-50429) | 4.44 (2.81-6.9) | 500 (432-599) | 1317 (1077-1610) | 1.63 (1.05-2.36) | 16908 (14563-19904) | 49409 (39271-60858) | 1.92 (1.26-2.78) |
| Palau | 4 (3-5) | 11 (8-15) | 1.97 (1.01-3.38) | 0 (0-0) | 0 (0-0) | 1.08 (0.5-1.79) | 4 (3-5) | 8 (6-10) | 1.13 (0.52-1.93) |
| Palestine | 190 (124-276) | 922 (636-1173) | 3.86 (2.23-6.7) | 6 (4-8) | 15 (11-18) | 1.46 (0.79-2.5) | 166 (116-226) | 444 (316-548) | 1.67 (0.91-2.85) |
| Panama | 239 (206-278) | 907 (658-1205) | 2.79 (1.65-4.33) | 9 (8-9) | 25 (19-31) | 1.85 (1.18-2.63) | 259 (236-285) | 652 (495-844) | 1.52 (0.88-2.28) |
| Papua New Guinea | 118 (82-172) | 526 (343-827) | 3.48 (2.05-5.6) | 9 (7-14) | 28 (20-43) | 1.99 (1.21-2.99) | 301 (223-451) | 910 (631-1402) | 2.02 (1.22-3.11) |
| Paraguay | 216 (178-260) | 1234 (860-1696) | 4.72 (2.88-7.17) | 13 (11-16) | 46 (32-60) | 2.41 (1.44-3.61) | 366 (311-426) | 1223 (844-1622) | 2.34 (1.35-3.57) |
| Peru | 1527 (1222-1912) | 7872 (5440-10822) | 4.16 (2.43-6.62) | 105 (88-125) | 264 (192-351) | 1.51 (0.8-2.49) | 3030 (2525-3578) | 6608 (4733-8900) | 1.18 (0.56-2.06) |
| Philippines | 11320 (8337-13868) | 29778 (22714-39024) | 1.63 (0.92-2.79) | 447 (361-532) | 1010 (812-1225) | 1.26 (0.74-1.97) | 13878 (10797-16349) | 29535 (23648-35586) | 1.13 (0.66-1.76) |
| Poland | 12242 (10992-13294) | 14015 (11065-18026) | 0.14 (-0.12-0.56) | 487 (424-504) | 365 (305-455) | -0.25 (-0.38-0.07) | 13133 (11125-13707) | 8366 (7022-10786) | -0.36 (-0.47--0.05) |
| Portugal | 3485 (3056-3912) | 5990 (4440-7804) | 0.72 (0.26-1.33) | 125 (113-135) | 116 (100-130) | -0.07 (-0.18-0.08) | 2641 (2404-2839) | 2393 (2092-2711) | -0.09 (-0.21-0.06) |
| Puerto Rico | 674 (588-758) | 949 (704-1269) | 0.41 (0.02-1) | 24 (21-26) | 27 (21-35) | 0.13 (-0.14-0.48) | 609 (545-664) | 612 (473-797) | 0.01 (-0.24-0.35) |
| Qatar | 32 (24-43) | 525 (367-757) | 15.3 (9.57-24.09) | 1 (0-1) | 3 (2-5) | 4.49 (2.81-6.92) | 21 (17-27) | 137 (99-194) | 5.42 (3.32-8.48) |
| Republic of Korea | 5407 (4266-11434) | 44075 (29771-56704) | 7.15 (1.64-11.01) | 140 (115-313) | 580 (426-679) | 3.14 (0.4-4.49) | 3986 (3296-8461) | 13779 (9787-16466) | 2.46 (0.17-3.59) |
| Republic of Moldova | 642 (559-723) | 1041 (835-1269) | 0.62 (0.29-1.01) | 21 (19-23) | 24 (20-28) | 0.15 (-0.02-0.33) | 643 (561-712) | 697 (577-832) | 0.08 (-0.09-0.28) |
| Romania | 3701 (3265-4151) | 6514 (5041-8177) | 0.76 (0.35-1.28) | 146 (134-155) | 163 (134-196) | 0.12 (-0.08-0.38) | 4218 (3764-4524) | 4120 (3312-5074) | -0.02 (-0.21-0.21) |
| Russian Federation | 30961 (29404-34110) | 70997 (58824-85551) | 1.29 (0.9-1.75) | 919 (873-987) | 1288 (1112-1482) | 0.4 (0.21-0.61) | 24637 (23235-26835) | 33718 (29037-39032) | 0.37 (0.19-0.56) |
| Rwanda | 319 (170-516) | 1124 (728-1675) | 2.53 (0.84-7.15) | 27 (19-39) | 49 (38-64) | 0.8 (0.14-1.95) | 1013 (649-1503) | 1702 (1239-2296) | 0.68 (0-2.04) |
| Saint Kitts and Nevis | 6 (6-7) | 18 (12-25) | 1.92 (0.88-3.06) | 0 (0-0) | 1 (0-1) | 0.63 (0.3-1.02) | 9 (8-10) | 17 (13-21) | 0.86 (0.38-1.39) |
| Saint Lucia | 16 (14-18) | 49 (39-60) | 2 (1.33-2.84) | 1 (1-1) | 2 (1-2) | 0.83 (0.53-1.19) | 23 (21-25) | 41 (34-49) | 0.8 (0.47-1.18) |
| Saint Vincent and the Grenadines | 13 (11-14) | 31 (25-37) | 1.39 (0.92-1.99) | 1 (1-1) | 1 (1-1) | 0.84 (0.57-1.16) | 18 (16-20) | 33 (28-39) | 0.86 (0.56-1.2) |
| Samoa | 20 (14-28) | 45 (27-66) | 1.3 (0.33-2.77) | 1 (1-1) | 1 (1-1) | 0.44 (0.05-0.97) | 24 (18-31) | 35 (25-47) | 0.45 (-0.02-1.12) |
| San Marino | 11 (9-14) | 25 (18-36) | 1.26 (0.54-2.29) | 0 (0-0) | 1 (0-1) | 0.74 (0.13-1.6) | 8 (6-9) | 12 (8-18) | 0.61 (0.02-1.45) |
| Sao Tome and Principe | 2 (1-3) | 7 (5-11) | 3.19 (1.61-6.17) | 0 (0-0) | 0 (0-0) | 1.19 (0.56-2.21) | 6 (4-8) | 13 (9-17) | 1.05 (0.34-2.18) |
| Saudi Arabia | 1095 (763-1590) | 20142 (13443-28317) | 17.4 (9.35-30.41) | 29 (21-46) | 121 (93-154) | 3.17 (1.49-5.2) | 963 (687-1417) | 5380 (3862-7281) | 4.59 (2.22-7.7) |
| Senegal | 96 (59-132) | 277 (176-387) | 1.89 (0.85-3.44) | 10 (6-12) | 20 (14-26) | 1.09 (0.56-1.82) | 298 (189-384) | 603 (411-785) | 1.02 (0.44-1.83) |
| Serbia | 1279 (1041-1519) | 2544 (1891-3378) | 0.99 (0.46-1.71) | 60 (50-70) | 85 (65-108) | 0.42 (0.08-0.86) | 1638 (1380-1896) | 2006 (1522-2596) | 0.22 (-0.07-0.61) |
| Seychelles | 3 (3-4) | 12 (10-15) | 2.44 (1.69-3.41) | 0 (0-0) | 0 (0-0) | 0.64 (0.36-0.97) | 6 (5-6) | 10 (8-11) | 0.73 (0.42-1.1) |
| Sierra Leone | 41 (24-60) | 142 (88-201) | 2.42 (1.1-4.38) | 5 (3-7) | 9 (6-12) | 0.79 (0.29-1.43) | 148 (91-196) | 293 (189-395) | 0.98 (0.39-1.81) |
| Singapore | 855 (757-973) | 2537 (1912-3263) | 1.97 (1.2-3.02) | 16 (15-17) | 33 (28-37) | 1.04 (0.77-1.4) | 445 (408-487) | 811 (693-964) | 0.82 (0.58-1.18) |
| Slovakia | 1174 (968-1340) | 1989 (1508-2642) | 0.69 (0.23-1.36) | 38 (33-42) | 41 (32-53) | 0.08 (-0.17-0.39) | 1005 (862-1109) | 1001 (771-1293) | 0 (-0.25-0.29) |
| Slovenia | 543 (393-714) | 811 (589-1089) | 0.49 (-0.03-1.26) | 17 (13-22) | 19 (14-25) | 0.15 (-0.19-0.62) | 413 (309-538) | 413 (307-544) | 0 (-0.32-0.47) |
| Solomon Islands | 23 (12-36) | 107 (55-176) | 3.7 (2.08-6.29) | 1 (1-2) | 3 (2-5) | 1.7 (0.84-2.73) | 45 (28-66) | 123 (76-180) | 1.75 (0.86-2.87) |
| Somalia | 211 (133-330) | 781 (466-1164) | 2.7 (1.18-5.34) | 19 (14-27) | 63 (43-88) | 2.27 (1.22-3.78) | 721 (512-1034) | 2318 (1565-3250) | 2.22 (1.15-3.82) |
| South Africa | 1624 (1386-1904) | 3107 (2628-4080) | 0.91 (0.57-1.77) | 84 (73-97) | 165 (144-188) | 0.97 (0.77-1.2) | 2608 (2352-2935) | 4377 (3883-5136) | 0.68 (0.49-0.96) |
| South Sudan | 145 (92-218) | 343 (205-578) | 1.37 (0.37-2.96) | 14 (10-18) | 25 (17-38) | 0.87 (0.27-1.73) | 454 (312-635) | 862 (550-1352) | 0.9 (0.24-1.96) |
| Spain | 9310 (8245-10236) | 19448 (14729-25936) | 1.09 (0.57-1.81) | 284 (261-302) | 419 (356-465) | 0.47 (0.3-0.64) | 6828 (6219-7265) | 8778 (7435-9934) | 0.29 (0.13-0.46) |
| Sri Lanka | 2338 (1771-2810) | 7099 (4928-10294) | 2.04 (0.98-3.55) | 91 (76-105) | 160 (117-215) | 0.76 (0.24-1.41) | 2720 (2194-3153) | 4189 (3009-5766) | 0.54 (0.05-1.14) |
| Sudan | 860 (473-1361) | 4572 (2675-6996) | 4.31 (2.12-8.03) | 43 (29-60) | 102 (71-135) | 1.36 (0.69-2.35) | 1359 (882-1935) | 3335 (2208-4667) | 1.45 (0.68-2.72) |
| Suriname | 25 (19-31) | 84 (65-107) | 2.31 (1.47-3.54) | 2 (1-2) | 4 (3-4) | 1.38 (0.93-1.94) | 43 (36-50) | 100 (81-121) | 1.31 (0.84-1.91) |
| Sweden | 1812 (1655-2018) | 2827 (2307-3433) | 0.56 (0.24-0.92) | 92 (84-100) | 97 (85-106) | 0.05 (-0.05-0.16) | 1778 (1648-1916) | 1830 (1653-2000) | 0.03 (-0.07-0.14) |
| Switzerland | 1498 (1326-1746) | 4050 (3001-5397) | 1.7 (0.85-2.74) | 40 (36-48) | 92 (74-105) | 1.3 (0.6-1.69) | 904 (821-1090) | 1828 (1442-2116) | 1.02 (0.37-1.36) |
| Syrian Arab Republic | 276 (190-371) | 1139 (733-1576) | 3.12 (1.72-5.53) | 9 (6-12) | 21 (13-28) | 1.18 (0.51-2.24) | 297 (207-381) | 619 (396-840) | 1.08 (0.41-2.12) |
| Taiwan (Province of China) | 4695 (4218-5237) | 14466 (10800-19758) | 2.08 (1.22-3.22) | 92 (86-98) | 217 (166-279) | 1.36 (0.79-2.02) | 2749 (2548-2958) | 5301 (4010-6962) | 0.93 (0.47-1.53) |
| Tajikistan | 64 (55-78) | 188 (147-242) | 1.92 (1.19-2.88) | 5 (3-8) | 10 (7-12) | 0.82 (0.27-1.46) | 147 (110-185) | 290 (216-364) | 0.98 (0.49-1.56) |
| Thailand | 7154 (5818-8971) | 20577 (13389-36196) | 1.88 (0.83-3.84) | 271 (231-325) | 577 (433-793) | 1.13 (0.56-1.87) | 8037 (6869-9629) | 13375 (9579-19927) | 0.66 (0.17-1.36) |
| Timor-Leste | 33 (18-49) | 136 (75-197) | 3.09 (1.03-5.94) | 3 (2-3) | 8 (5-11) | 2.1 (1.12-3.4) | 84 (53-114) | 211 (138-286) | 1.52 (0.57-2.73) |
| Togo | 55 (33-76) | 173 (108-248) | 2.14 (0.96-3.83) | 4 (2-5) | 10 (6-13) | 1.54 (0.89-2.39) | 130 (82-167) | 319 (213-434) | 1.45 (0.73-2.36) |
| Tokelau | 0 (0-0) | 0 (0-0) | 1.04 (0.28-2.16) | 0 (0-0) | 0 (0-0) | -0.12 (-0.36-0.22) | 0 (0-0) | 0 (0-0) | -0.08 (-0.36-0.3) |
| Tonga | 5 (4-7) | 11 (7-15) | 1.13 (0.4-2.23) | 0 (0-0) | 1 (0-1) | 0.77 (0.3-1.39) | 8 (6-10) | 13 (9-17) | 0.61 (0.15-1.22) |
| Trinidad and Tobago | 103 (92-114) | 256 (183-349) | 1.48 (0.72-2.49) | 5 (5-6) | 9 (7-12) | 0.76 (0.31-1.29) | 150 (140-161) | 252 (187-331) | 0.68 (0.22-1.22) |
| Tunisia | 942 (715-1216) | 4233 (2851-6113) | 3.49 (1.8-5.97) | 20 (16-26) | 49 (36-68) | 1.53 (0.79-2.6) | 629 (514-783) | 1497 (1064-2055) | 1.38 (0.6-2.49) |
| Trkiye | 6445 (4571-8442) | 27170 (20659-35897) | 3.22 (1.87-5.43) | 257 (196-316) | 455 (353-635) | 0.77 (0.27-1.47) | 7459 (5589-9182) | 12071 (9272-16054) | 0.62 (0.14-1.29) |
| Turkmenistan | 185 (163-207) | 635 (488-824) | 2.44 (1.61-3.46) | 8 (8-9) | 16 (13-20) | 0.9 (0.51-1.41) | 279 (249-305) | 550 (436-698) | 0.97 (0.55-1.5) |
| Tuvalu | 1 (1-1) | 2 (1-3) | 1.19 (0.47-2.38) | 0 (0-0) | 0 (0-0) | 0.39 (0-0.97) | 2 (1-2) | 2 (2-3) | 0.32 (-0.04-0.91) |
| Uganda | 389 (256-571) | 3161 (2011-4660) | 7.12 (3.32-12.92) | 33 (25-45) | 139 (98-176) | 3.21 (1.37-4.97) | 1116 (815-1543) | 5076 (3410-6801) | 3.55 (1.55-5.91) |
| Ukraine | 10343 (8577-13598) | 19254 (15231-23859) | 0.86 (0.25-1.58) | 325 (281-400) | 392 (329-467) | 0.21 (-0.09-0.51) | 8741 (7534-10998) | 11049 (9280-13079) | 0.26 (-0.07-0.59) |
| United Arab Emirates | 196 (127-281) | 3898 (1996-6221) | 18.85 (10.7-30.05) | 4 (2-5) | 42 (22-66) | 10.12 (5.87-15.62) | 157 (99-222) | 1873 (966-2948) | 10.95 (6.19-17.1) |
| United Kingdom | 12314 (11525-12922) | 21971 (17972-26441) | 0.78 (0.45-1.17) | 410 (384-428) | 499 (452-530) | 0.22 (0.14-0.32) | 9041 (8448-9583) | 10523 (9788-11330) | 0.16 (0.09-0.27) |
| United Republic of Tanzania | 1055 (744-1474) | 4598 (3069-6812) | 3.36 (1.76-5.99) | 74 (59-94) | 212 (160-274) | 1.85 (1.07-2.8) | 2563 (1980-3325) | 7220 (5233-9830) | 1.82 (0.96-3) |
| United States of America | 97065 (93652-100203) | 220711 (187749-257904) | 1.29 (0.69-2.22) | 1252 (1167-1301) | 2466 (2231-2582) | 1.55 (0.99-2.25) | 32935 (30601-35369) | 62493 (56922-67954) | 1.15 (0.67-1.81) |
| United States Virgin Islands | 10 (8-12) | 22 (17-27) | 1.27 (0.94-1.65) | 0 (0-0) | 1 (1-1) | 0.97 (0.89-1.05) | 12 (9-14) | 25 (20-30) | 0.9 (0.79-1) |
| Uruguay | 300 (259-354) | 837 (626-1096) | 1.79 (0.99-2.86) | 16 (15-19) | 30 (27-34) | 0.84 (0.55-1.12) | 389 (349-451) | 679 (610-748) | 0.75 (0.46-1.01) |
| Uzbekistan | 292 (244-399) | 1911 (1534-2330) | 5.54 (3.46-7.8) | 17 (13-28) | 54 (45-65) | 2.22 (0.87-3.55) | 470 (390-697) | 1731 (1429-2084) | 2.68 (1.38-3.99) |
| Vanuatu | 6 (4-9) | 23 (14-34) | 3.13 (1.64-5.69) | 0 (0-1) | 1 (1-2) | 2.22 (1.31-3.56) | 13 (9-19) | 41 (28-58) | 2.17 (1.23-3.65) |
| Venezuela (Bolivarian Republic of) | 863 (779-967) | 5483 (3956-7455) | 5.35 (3.5-7.78) | 48 (44-51) | 184 (139-239) | 2.83 (1.88-4) | 1343 (1257-1449) | 4781 (3573-6302) | 2.56 (1.62-3.74) |
| Viet Nam | 7479 (5269-10363) | 64088 (38833-87569) | 7.57 (4.15-12.56) | 373 (279-602) | 1204 (925-1533) | 2.23 (1.03-3.6) | 10834 (8103-15189) | 36453 (26526-47439) | 2.36 (1.09-3.86) |
| Yemen | 466 (254-761) | 2806 (1699-4195) | 5.02 (2.57-9.58) | 23 (16-34) | 72 (52-97) | 2.1 (1.14-3.58) | 734 (475-1090) | 2359 (1630-3283) | 2.22 (1.11-4.02) |
| Zambia | 350 (224-528) | 1809 (1175-2681) | 4.17 (2.03-8.34) | 24 (18-32) | 67 (50-88) | 1.83 (0.89-3.1) | 906 (648-1250) | 2547 (1817-3477) | 1.81 (0.8-3.27) |
| Zimbabwe | 557 (389-732) | 1453 (768-2123) | 1.61 (0.69-2.92) | 32 (26-39) | 75 (50-101) | 1.32 (0.7-2.12) | 1010 (790-1223) | 2527 (1582-3497) | 1.5 (0.76-2.49) |

Abbreviations: UI=uncertainty interval.

**Supplementary Table S3 :**Age-standardised prevalence, death and DALY rates forThyroid cancer (TC) by 204 countries or territories in 1990 and 2019, and their temporal trends from 1990 to 2019

|  | **Age-standardised prevalence rate**  **per 100,000 population** | | | **Age-standardized death rate**  **per 100,000 population** | | | **Age-standardized DALY rate**  **per 100,000 population** | | |
| --- | --- | --- | --- | --- | --- | --- | --- | --- | --- |
| **Characteristics** | **1990no**  **(95%UI)** | **2019no**  **(95%UI)** | **EAPC No.**  **(95%CI)** | **1990no**  **(95%UI)** | **2019no**  **(95%UI)** | **EAPC No.**  **(95%CI)** | **1990no**  **(95%UI)** | **2019no**  **(95%UI)** | **EAPC No.**  **(95%CI)** |
| Afghanistan | 8.51 (3.55-14.85) | 13.74 (6.66-23.08) | 1.88% (1.75-2.01) | 0.8 (0.5-1.16) | 0.89 (0.62-1.24) | 0.52% (0.43-0.61) | 22.71 (12.48-35.48) | 24.18 (15.22-35.44) | 0.33% (0.22-0.44) |
| Albania | 11.91 (8.93-13.5) | 22.31 (16.4-29.93) | 1.98% (1.8-2.16) | 0.61 (0.55-0.68) | 0.45 (0.33-0.6) | -1.25% (-1.49--1) | 16.18 (14.08-17.71) | 12.57 (9.34-16.63) | -1.07% (-1.32--0.82) |
| Algeria | 16.56 (12.19-21.95) | 36.22 (24.02-49.53) | 2.83% (2.74-2.93) | 0.5 (0.4-0.77) | 0.54 (0.43-0.67) | 0.42% (0.31-0.53) | 13.99 (10.98-18.76) | 15.13 (11.66-19.13) | 0.44% (0.35-0.54) |
| American Samoa | 13.18 (10.27-17.66) | 23.9 (17.51-31.81) | 2.37% (2.01-2.74) | 1.06 (0.83-1.32) | 1.2 (0.79-1.53) | 0.66% (0.41-0.91) | 22.13 (17.8-27.53) | 26.34 (18.54-33.43) | 0.79% (0.55-1.04) |
| Andorra | 25.21 (18.09-35.96) | 37.93 (27.05-50.99) | 1.33% (1.07-1.59) | 0.66 (0.49-0.89) | 0.56 (0.42-0.72) | -0.57% (-0.63--0.52) | 15.86 (11.69-22.1) | 14.42 (10.4-18.97) | -0.4% (-0.43--0.37) |
| Angola | 2.12 (1.45-3.02) | 4.02 (2.64-6.13) | 2.29% (2.12-2.47) | 0.49 (0.34-0.68) | 0.5 (0.34-0.74) | 0.04% (0.01-0.08) | 12.43 (8.76-17.2) | 11.95 (8.32-17.69) | -0.19% (-0.24--0.14) |
| Antigua and Barbuda | 13.93 (11.88-16.31) | 23.78 (19.02-29.6) | 1.97% (1.68-2.26) | 0.65 (0.58-0.73) | 0.8 (0.68-0.94) | 0.73% (0.42-1.03) | 16.28 (14.42-18.24) | 19.19 (16.07-22.61) | 0.58% (0.27-0.89) |
| Argentina | 14.09 (12.26-15.7) | 17.41 (13.15-22.89) | 0.37% (0.14-0.6) | 0.78 (0.7-0.83) | 0.54 (0.5-0.6) | -1.38% (-1.64--1.13) | 19.9 (17.3-21.23) | 13.84 (12.65-15.3) | -1.39% (-1.67--1.11) |
| Armenia | 5.02 (4.37-6.16) | 16.98 (13.76-20.58) | 5.21% (4.55-5.88) | 0.3 (0.27-0.38) | 0.63 (0.52-0.74) | 3.03% (2.34-3.72) | 7.37 (6.54-9.81) | 14.43 (11.99-17.07) | 2.82% (2.16-3.49) |
| Australia | 19.95 (18.03-22.05) | 41.28 (31.22-54.53) | 3.01% (2.6-3.41) | 0.41 (0.38-0.44) | 0.44 (0.38-0.49) | 0.79% (0.6-0.98) | 10.61 (9.83-11.55) | 12.31 (10.54-14.14) | 1.01% (0.81-1.2) |
| Austria | 41.4 (35.67-46.43) | 36.64 (28.63-46.78) | -0.87% (-1.07--0.66) | 0.83 (0.74-0.89) | 0.47 (0.41-0.53) | -2.28% (-2.42--2.14) | 20.4 (18.03-22.2) | 11.89 (10.4-13.61) | -2.23% (-2.39--2.08) |
| Azerbaijan | 7.95 (6.53-9.69) | 12.96 (9.92-16.63) | 1.64% (1.37-1.91) | 0.43 (0.36-0.58) | 0.47 (0.36-0.64) | 0.15% (0.03-0.26) | 12.58 (10.78-15.66) | 12.47 (9.96-16.22) | -0.34% (-0.48--0.2) |
| Bahamas | 14.16 (12.31-16.07) | 19.5 (15.25-25.1) | 1.38% (1.23-1.52) | 0.68 (0.62-0.75) | 0.65 (0.53-0.79) | 0.08% (-0.06-0.21) | 18.56 (16.74-20.54) | 17.85 (14.38-22.23) | 0.11% (-0.03-0.26) |
| Bahrain | 12.27 (9.73-15.64) | 25.92 (19.39-33.27) | 3.25% (2.65-3.85) | 0.82 (0.66-0.97) | 0.73 (0.49-0.93) | 0.14% (-0.46-0.74) | 17.1 (14.09-20.51) | 14.63 (10.42-18.65) | -0.2% (-0.72-0.31) |
| Bangladesh | 4.97 (3.42-6.97) | 10.74 (7.25-16.19) | 2.85% (2.66-3.03) | 0.58 (0.45-0.75) | 0.49 (0.37-0.68) | -0.47% (-0.65--0.28) | 16.69 (12.53-21.92) | 13.76 (9.9-19.08) | -0.55% (-0.67--0.44) |
| Barbados | 13.92 (12.28-15.72) | 22.17 (17.58-27.97) | 1.67% (1.53-1.81) | 0.65 (0.59-0.71) | 0.67 (0.56-0.8) | 0.24% (0.14-0.34) | 16.35 (14.96-17.81) | 16.93 (13.85-20.51) | 0.23% (0.12-0.33) |
| Belarus | 18.26 (15.8-21.32) | 35.5 (25.82-47.9) | 2.12% (1.86-2.37) | 0.48 (0.43-0.55) | 0.61 (0.48-0.77) | 0.52% (0.13-0.92) | 13.53 (12.22-15.38) | 16.42 (12.5-21.32) | 0.24% (-0.18-0.66) |
| Belgium | 26.15 (22.68-29.18) | 28.49 (21.44-36.76) | 0.37% (0.17-0.57) | 0.99 (0.88-1.07) | 0.47 (0.42-0.53) | -2.56% (-3.09--2.04) | 20.89 (18.72-22.48) | 11.91 (10.41-13.58) | -1.91% (-2.28--1.53) |
| Belize | 3.98 (3.44-4.56) | 9.16 (7.58-10.81) | 2.79% (2.53-3.05) | 0.3 (0.27-0.33) | 0.37 (0.31-0.42) | 0.88% (0.66-1.1) | 7.35 (6.64-8.14) | 10.09 (8.64-11.6) | 1.15% (0.92-1.37) |
| Benin | 1.9 (1.12-2.57) | 2.54 (1.66-3.64) | 0.89% (0.77-1) | 0.3 (0.2-0.36) | 0.28 (0.2-0.36) | -0.23% (-0.3--0.16) | 7.6 (4.93-9.42) | 6.98 (4.94-9.36) | -0.33% (-0.4--0.26) |
| Bermuda | 23.04 (20.15-26.69) | 32.69 (25.79-41.14) | 1.15% (1.03-1.27) | 0.98 (0.88-1.09) | 0.64 (0.52-0.78) | -1.5% (-1.75--1.24) | 23.74 (21.34-26.41) | 15.97 (12.98-19.78) | -1.36% (-1.62--1.1) |
| Bhutan | 4.91 (2.8-7.52) | 13.97 (9.08-20.68) | 3.64% (3.55-3.74) | 0.62 (0.44-0.91) | 0.71 (0.53-0.97) | 0.38% (0.34-0.42) | 17.14 (11.54-25.02) | 17.99 (12.9-25.45) | 0.03% (-0.03-0.08) |
| Bolivia (Plurinational State of) | 8.73 (5.79-12.3) | 22.04 (15.08-30.76) | 3.01% (2.85-3.17) | 1.47 (1.09-1.92) | 1.71 (1.27-2.18) | 0.51% (0.49-0.54) | 35.88 (26.11-47.36) | 38.49 (28.39-50.01) | 0.17% (0.14-0.2) |
| Bosnia and Herzegovina | 10.18 (6.88-11.9) | 17.15 (11.74-22.91) | 2.16% (1.74-2.58) | 0.52 (0.38-0.57) | 0.4 (0.3-0.5) | -1.19% (-1.35--1.04) | 13.16 (9.34-14.59) | 10.08 (7.5-12.92) | -1.19% (-1.33--1.06) |
| Botswana | 0.88 (0.62-1.24) | 2.2 (1.4-3.09) | 2.73% (2.44-3.01) | 0.08 (0.06-0.11) | 0.1 (0.08-0.13) | 0.28% (-0.04-0.59) | 2.33 (1.75-3.15) | 2.93 (2.09-3.93) | 0.37% (0.02-0.71) |
| Brazil | 9.01 (8.56-9.57) | 12.99 (12.15-14.46) | 1.28% (0.92-1.64) | 0.69 (0.65-0.72) | 0.52 (0.47-0.58) | -0.87% (-0.97--0.77) | 16.79 (16.06-17.65) | 12.63 (11.84-14.5) | -0.88% (-1.01--0.75) |
| Brunei Darussalam | 23.15 (17.09-30.42) | 37.47 (29.58-46.51) | 2.05% (1.83-2.26) | 1.27 (1.01-1.53) | 1.25 (1.01-1.45) | 0.29% (0.15-0.44) | 27.13 (21.51-32.82) | 26.03 (21.17-30.3) | 0.24% (0.09-0.4) |
| Bulgaria | 13.79 (11.93-15.59) | 17.4 (13.01-22.67) | 0.8% (0.6-1.01) | 0.56 (0.52-0.61) | 0.46 (0.36-0.57) | -0.9% (-0.99--0.82) | 15.98 (14.37-17.42) | 13.23 (10.16-16.84) | -0.81% (-0.91--0.7) |
| Burkina Faso | 1.95 (1.17-2.63) | 2.71 (1.76-3.71) | 1.08% (0.95-1.22) | 0.31 (0.2-0.39) | 0.29 (0.19-0.36) | -0.35% (-0.51--0.19) | 7.82 (4.96-9.9) | 7.25 (4.98-9.2) | -0.39% (-0.58--0.21) |
| Burundi | 5.77 (3.22-9.16) | 7.92 (5.41-11.15) | 1.25% (1.1-1.39) | 0.78 (0.54-1.1) | 0.75 (0.54-0.99) | -0.25% (-0.31--0.2) | 22.83 (15.08-33.61) | 20.87 (14.95-28.06) | -0.5% (-0.57--0.42) |
| Cabo Verde | 2.43 (1.95-3.05) | 6.59 (4.67-8.72) | 3.63% (3.29-3.96) | 0.24 (0.16-0.29) | 0.3 (0.22-0.4) | 0.09% (-0.18-0.36) | 5.95 (4.35-6.97) | 7.76 (5.74-10.05) | 0.47% (0.23-0.7) |
| Cambodia | 10.31 (5.63-14.7) | 23.33 (13.94-32.81) | 2.81% (2.72-2.91) | 1.24 (0.84-1.59) | 1.33 (0.9-1.67) | 0.12% (0.07-0.18) | 33.01 (21.04-42.71) | 32.5 (20.95-42.23) | -0.18% (-0.25--0.12) |
| Cameroon | 1.4 (1.02-1.85) | 2.13 (1.37-3.2) | 1.42% (1.27-1.57) | 0.16 (0.13-0.21) | 0.15 (0.11-0.21) | -0.28% (-0.36--0.19) | 4.36 (3.43-5.43) | 4.09 (2.88-5.67) | -0.26% (-0.35--0.17) |
| Canada | 24.74 (22.08-27.47) | 32.36 (24.32-42.16) | 0.76% (0.33-1.18) | 0.44 (0.4-0.47) | 0.41 (0.36-0.45) | -0.31% (-0.56--0.05) | 11.38 (10.54-12.3) | 10.84 (9.48-12.27) | -0.33% (-0.61--0.04) |
| Central African Republic | 2.6 (1.75-3.59) | 2.44 (1.6-3.62) | 0.03% (-0.09-0.15) | 0.61 (0.44-0.82) | 0.57 (0.37-0.86) | -0.19% (-0.24--0.13) | 15.64 (11.37-21.01) | 14.09 (9.39-20.8) | -0.32% (-0.37--0.26) |
| Chad | 1.52 (0.86-2.14) | 1.78 (1.13-2.44) | 0.5% (0.37-0.63) | 0.27 (0.16-0.34) | 0.26 (0.17-0.33) | -0.12% (-0.22--0.02) | 6.77 (4.09-8.76) | 6.45 (4.37-8.48) | -0.18% (-0.29--0.08) |
| Chile | 12.58 (11.13-14.51) | 25.22 (18.9-33.15) | 2.6% (2.45-2.74) | 0.77 (0.7-0.85) | 0.67 (0.59-0.74) | -0.48% (-0.55--0.41) | 17.79 (16.28-19.48) | 15.27 (13.65-17.03) | -0.53% (-0.61--0.45) |
| China | 6.29 (5.14-7.53) | 16.17 (13.28-19.83) | 3.52% (3.36-3.67) | 0.42 (0.37-0.53) | 0.39 (0.32-0.45) | 0.06% (-0.09-0.2) | 10.87 (9.33-13.21) | 9.7 (8.11-11.27) | -0.2% (-0.31--0.1) |
| Colombia | 14.77 (13.3-16.64) | 23.52 (17.31-32.01) | 1.63% (1.41-1.84) | 1.09 (1.01-1.16) | 0.75 (0.58-0.94) | -1.26% (-1.53--0.98) | 26.66 (25-28.36) | 17.96 (13.81-23.03) | -1.32% (-1.61--1.03) |
| Comoros | 4.31 (1.29-6.82) | 9.75 (5.91-14.74) | 2.9% (2.6-3.2) | 0.59 (0.34-0.8) | 0.76 (0.56-0.98) | 0.87% (0.77-0.97) | 16.45 (7.68-23.51) | 20.62 (14.46-27.76) | 0.7% (0.5-0.91) |
| Congo | 3.61 (2.47-5) | 4.77 (3.19-6.74) | 1.32% (1.06-1.57) | 0.68 (0.5-0.88) | 0.56 (0.4-0.75) | -0.72% (-0.83--0.62) | 17 (12.88-21.5) | 13.33 (9.56-18.03) | -0.92% (-1.04--0.8) |
| Cook Islands | 18.65 (14.14-24.44) | 20.47 (14.34-27.48) | -0.42% (-0.77--0.06) | 0.83 (0.66-1.02) | 0.54 (0.44-0.66) | -2.25% (-2.66--1.84) | 20.93 (16.7-26.39) | 13.77 (10.8-17.28) | -2.24% (-2.67--1.82) |
| Costa Rica | 20.54 (18.02-23.28) | 28.22 (20.87-37.79) | 0.83% (0.55-1.12) | 0.75 (0.68-0.82) | 0.67 (0.51-0.86) | -0.72% (-0.96--0.48) | 19.51 (17.77-21.13) | 17.27 (13.12-22.5) | -0.76% (-1.01--0.51) |
| Cte d'Ivoire | 1.98 (1.23-2.64) | 2.28 (1.46-3.22) | 0.49% (0.42-0.56) | 0.32 (0.21-0.39) | 0.28 (0.19-0.36) | -0.57% (-0.68--0.46) | 7.97 (5.2-10.11) | 6.87 (4.78-9.12) | -0.69% (-0.82--0.56) |
| Croatia | 36.2 (30.74-41.46) | 32.37 (23.97-43.19) | 0.14% (-0.17-0.46) | 0.82 (0.71-0.91) | 0.45 (0.35-0.56) | -1.68% (-1.99--1.37) | 21.57 (18.28-23.93) | 11.81 (9.04-15.51) | -1.71% (-2--1.41) |
| Cuba | 14.32 (12.81-16.17) | 24.6 (19.04-31.4) | 2% (1.84-2.16) | 0.47 (0.43-0.51) | 0.57 (0.46-0.7) | 0.9% (0.7-1.1) | 12.67 (11.69-13.8) | 14.91 (11.88-18.65) | 0.73% (0.53-0.93) |
| Cyprus | 18.79 (13.07-23.14) | 25.61 (20.71-32.17) | 0.81% (0.38-1.25) | 0.85 (0.62-1.01) | 0.49 (0.4-0.57) | -2.26% (-2.38--2.13) | 18.91 (13.95-22.02) | 11.38 (9.38-13.59) | -2.15% (-2.33--1.98) |
| Czechia | 36.09 (32.36-40.15) | 34.55 (26.27-44.73) | -0.35% (-0.64--0.06) | 0.84 (0.76-0.89) | 0.43 (0.34-0.52) | -2.32% (-2.44--2.2) | 21.24 (19.35-22.89) | 11.17 (8.87-13.97) | -2.22% (-2.34--2.09) |
| Democratic People's Republic of Korea | 19.06 (11.86-27.68) | 18.38 (12.54-26.97) | -0.13% (-0.44-0.18) | 0.6 (0.45-0.76) | 0.52 (0.42-0.65) | -0.4% (-0.51--0.29) | 17.08 (12.34-22.86) | 14.6 (11.06-18.82) | -0.51% (-0.57--0.45) |
| Democratic Republic of the Congo | 2.49 (1.69-3.5) | 3.18 (2.1-4.67) | 0.74% (0.56-0.92) | 0.49 (0.33-0.7) | 0.47 (0.3-0.71) | -0.09% (-0.12--0.07) | 12.12 (8.52-17.14) | 11.18 (7.38-16.45) | -0.29% (-0.32--0.26) |
| Denmark | 11.54 (10.25-13.3) | 23.15 (17.41-30.56) | 2.29% (1.88-2.71) | 0.34 (0.31-0.39) | 0.42 (0.37-0.47) | 0.51% (0.13-0.88) | 8.57 (7.87-10.19) | 10.5 (9.07-11.92) | 0.37% (0-0.74) |
| Djibouti | 4.46 (2.91-6.55) | 9.97 (5.76-16.49) | 3.25% (2.95-3.55) | 0.56 (0.41-0.74) | 0.76 (0.55-1.06) | 1.26% (1.15-1.36) | 15.16 (10.77-20.73) | 20.23 (13.41-29.68) | 1.16% (1.05-1.26) |
| Dominica | 10.95 (9-13.12) | 13.15 (9.93-16.93) | 0.81% (0.67-0.94) | 0.64 (0.56-0.73) | 0.69 (0.55-0.85) | 0.54% (0.37-0.71) | 16.2 (14-18.56) | 17.34 (13.67-21.66) | 0.45% (0.31-0.59) |
| Dominican Republic | 6.17 (4.5-7.5) | 16.29 (10.17-23.41) | 3.45% (3.27-3.63) | 0.51 (0.37-0.6) | 0.72 (0.5-0.96) | 1.68% (1.32-2.04) | 13.25 (9.79-15.54) | 19 (12.51-26.28) | 1.68% (1.32-2.04) |
| Ecuador | 7.79 (6.45-12.76) | 31.91 (23.62-43.3) | 4.98% (4.27-5.7) | 0.8 (0.69-1.27) | 1.5 (1.06-1.94) | 2.77% (2.39-3.15) | 19.27 (16.65-30.68) | 33.46 (24.88-43.54) | 2.31% (1.92-2.71) |
| Egypt | 9.19 (7.59-11.08) | 18.75 (13.05-25.54) | 2.37% (2.17-2.58) | 0.4 (0.34-0.69) | 0.42 (0.28-0.74) | 0.25% (0.18-0.33) | 12.52 (10.72-16.96) | 12.38 (8.64-18.38) | 0.12% (0.02-0.23) |
| El Salvador | 13.35 (6.71-16.05) | 15.54 (11.06-21.69) | 0.51% (0.1-0.92) | 1.14 (0.58-1.31) | 0.58 (0.44-0.76) | -2.37% (-2.98--1.76) | 29.46 (15.26-33.88) | 14.57 (10.86-19.59) | -2.43% (-3.05--1.8) |
| Equatorial Guinea | 2.04 (1.34-2.95) | 5.62 (3.29-9.38) | 4.26% (3.94-4.58) | 0.52 (0.36-0.72) | 0.51 (0.35-0.75) | -0.04% (-0.15-0.07) | 13.31 (9.25-18.39) | 11.66 (7.72-17.86) | -0.46% (-0.62--0.31) |
| Eritrea | 3.47 (2.2-5.34) | 9.13 (5.81-13.76) | 3.44% (3.27-3.61) | 0.66 (0.45-0.97) | 0.97 (0.67-1.33) | 1.47% (1.31-1.63) | 18.86 (13.24-27.4) | 25.98 (17.62-36.53) | 1.27% (1.13-1.42) |
| Estonia | 25.33 (22.07-29.08) | 36.17 (27.02-48.53) | 1.31% (1.09-1.54) | 0.67 (0.62-0.74) | 0.62 (0.47-0.78) | -0.74% (-0.97--0.52) | 18.31 (16.67-20.04) | 14.95 (11.36-19.35) | -1.23% (-1.46--0.99) |
| Eswatini | 3.84 (2.72-5.23) | 6.14 (3.63-9.24) | 1.48% (1.3-1.66) | 0.53 (0.37-0.67) | 0.63 (0.41-0.85) | 0.76% (0.25-1.27) | 12.92 (9.33-16.56) | 15.55 (10.06-21.7) | 0.82% (0.27-1.38) |
| Ethiopia | 19.5 (9.25-32.25) | 23.03 (16.22-31.79) | 0.24% (-0.04-0.52) | 2.88 (1.9-4.01) | 1.92 (1.4-2.46) | -1.64% (-1.8--1.49) | 85.92 (51.79-126.3) | 50.69 (37.62-65.28) | -2.15% (-2.31--1.99) |
| Fiji | 20.45 (10.93-27.47) | 24.21 (12.98-33.91) | 0.48% (0.29-0.67) | 1.58 (0.92-2.05) | 1.4 (0.83-1.81) | -0.48% (-0.7--0.25) | 39.08 (22.42-50.45) | 33.27 (19.7-43.98) | -0.65% (-0.83--0.47) |
| Finland | 23.88 (21.39-26.54) | 35.09 (26.59-46.01) | 1.66% (1.3-2.03) | 0.69 (0.63-0.74) | 0.54 (0.46-0.59) | -0.71% (-0.85--0.58) | 16.37 (15.13-17.69) | 13.16 (11.38-15) | -0.53% (-0.7--0.35) |
| France | 24.7 (22.14-27.15) | 27.87 (20.83-36.57) | 0.32% (-0.44-1.08) | 0.76 (0.69-0.8) | 0.42 (0.37-0.47) | -2.17% (-2.49--1.86) | 17.86 (16.68-18.97) | 10.89 (9.51-12.44) | -1.85% (-2.35--1.34) |
| Gabon | 4.18 (3.12-5.43) | 6.19 (4.26-8.71) | 1.22% (1.11-1.33) | 0.63 (0.48-0.79) | 0.58 (0.44-0.74) | -0.37% (-0.47--0.28) | 15.35 (11.89-19.31) | 13.69 (10.09-17.58) | -0.51% (-0.62--0.4) |
| Gambia | 1.78 (1.2-2.49) | 3.16 (2.18-4.35) | 1.76% (1.5-2.01) | 0.26 (0.2-0.33) | 0.3 (0.24-0.38) | 0.46% (0.32-0.61) | 6.29 (4.62-8.32) | 7.59 (5.75-9.81) | 0.53% (0.34-0.72) |
| Georgia | 9.53 (7.76-11.4) | 18.91 (14.49-23.27) | 2.9% (2.46-3.34) | 0.37 (0.31-0.42) | 0.55 (0.43-0.66) | 2.29% (1.76-2.82) | 9.77 (8.24-11.27) | 15.42 (11.96-18.55) | 2.39% (1.89-2.89) |
| Germany | 30.8 (27.59-33.82) | 35.16 (26.23-46.93) | 0.26% (0.12-0.41) | 0.82 (0.74-0.86) | 0.5 (0.44-0.55) | -1.91% (-2.17--1.65) | 19.93 (17.65-21.2) | 13 (11.52-14.67) | -1.69% (-1.91--1.46) |
| Ghana | 0.97 (0.72-1.27) | 1.57 (1.13-2.08) | 1.19% (1.03-1.35) | 0.17 (0.11-0.22) | 0.19 (0.13-0.25) | 0.32% (0.2-0.43) | 4.36 (3-5.55) | 4.69 (3.38-6.13) | 0.16% (0.04-0.29) |
| Greece | 17.08 (15.31-18.83) | 22.22 (16.9-29.16) | 0.66% (0.39-0.94) | 0.58 (0.52-0.63) | 0.4 (0.36-0.44) | -1.84% (-2.11--1.58) | 12.19 (11.22-13.1) | 9.66 (8.65-10.83) | -1.21% (-1.38--1.04) |
| Greenland | 12.19 (9.26-15.12) | 15.37 (10.9-20.19) | 1.05% (0.74-1.36) | 0.58 (0.5-0.73) | 0.64 (0.41-0.8) | 0.28% (-0.01-0.58) | 15.43 (12.93-18.35) | 14.59 (9.83-18.52) | -0.12% (-0.37-0.14) |
| Grenada | 18.65 (15.91-21.49) | 20.51 (16.87-24.73) | 0.43% (0.06-0.8) | 1.24 (1.1-1.37) | 0.97 (0.86-1.13) | -0.41% (-0.74--0.08) | 32.54 (28.71-36.22) | 24.34 (21.12-28.53) | -0.56% (-0.9--0.22) |
| Guam | 9.29 (7.57-13.42) | 24.93 (16.88-31.42) | 3.72% (2.95-4.49) | 0.5 (0.42-0.69) | 0.74 (0.46-0.91) | 1.79% (1.36-2.22) | 10.49 (8.78-14.9) | 19.51 (11.97-24.48) | 2.69% (2.24-3.14) |
| Guatemala | 5.43 (4.57-6.44) | 11.7 (8.8-15.32) | 1.6% (1.06-2.13) | 0.76 (0.67-0.85) | 0.68 (0.54-0.83) | -1.58% (-2.1--1.07) | 18.79 (16.51-21.22) | 16.92 (13.18-21.33) | -1.51% (-2.01--1) |
| Guinea | 2.6 (2-3.32) | 3.92 (2.74-5.35) | 1.55% (1.43-1.66) | 0.4 (0.33-0.52) | 0.43 (0.33-0.55) | 0.34% (0.28-0.4) | 10.9 (9.02-13.59) | 11.74 (8.87-15.35) | 0.4% (0.35-0.46) |
| Guinea-Bissau | 2.37 (1.35-3.46) | 2.81 (1.76-3.94) | 0.63% (0.52-0.74) | 0.41 (0.26-0.53) | 0.35 (0.23-0.45) | -0.54% (-0.58--0.5) | 11.13 (7.01-14.92) | 9.12 (6.07-11.86) | -0.68% (-0.72--0.63) |
| Guyana | 7.38 (6.02-8.9) | 13.28 (9.72-17.9) | 1.73% (1.52-1.93) | 0.69 (0.59-0.79) | 0.72 (0.56-0.91) | 0.31% (0.23-0.39) | 18.36 (15.69-21.18) | 20.05 (15.34-25.81) | 0.49% (0.41-0.57) |
| Haiti | 5.7 (3-8.77) | 8.03 (4.65-12.33) | 1.4% (1.23-1.57) | 0.98 (0.65-1.32) | 0.82 (0.53-1.19) | -0.4% (-0.53--0.26) | 25.95 (16.08-35.35) | 21.57 (13.56-31.63) | -0.41% (-0.56--0.26) |
| Honduras | 17.33 (6.33-22.95) | 48.13 (12.97-74.05) | 3.46% (3.12-3.8) | 1.87 (0.66-2.33) | 3.17 (0.95-4.35) | 1.94% (1.7-2.18) | 48.65 (18.48-61.26) | 76.23 (23.25-108.35) | 1.62% (1.42-1.83) |
| Hungary | 24.2 (21.4-27) | 26.12 (20.27-32.98) | -0.11% (-0.31-0.08) | 0.89 (0.8-0.95) | 0.52 (0.42-0.63) | -2.11% (-2.37--1.86) | 22.54 (20.24-24.1) | 13.3 (10.74-16.4) | -2.09% (-2.29--1.88) |
| Iceland | 67.38 (59.67-76.36) | 71.43 (59.51-84.72) | 0.05% (-0.27-0.38) | 1.39 (1.26-1.53) | 1 (0.87-1.14) | -1.16% (-1.33--0.99) | 35.55 (32.38-39.28) | 25.92 (22.44-29.58) | -1.15% (-1.29--1) |
| India | 5.02 (4.06-6.43) | 12.14 (9.79-14.74) | 3.11% (2.94-3.28) | 0.54 (0.45-0.7) | 0.63 (0.53-0.73) | 0.48% (0.33-0.63) | 14.83 (12.42-19.55) | 17.2 (14.37-20.14) | 0.47% (0.32-0.62) |
| Indonesia | 9.29 (6.23-11.51) | 16.93 (11.57-21.58) | 2% (1.85-2.16) | 0.84 (0.65-0.98) | 1 (0.75-1.18) | 0.7% (0.58-0.82) | 21.87 (16.06-25.6) | 23.79 (17.49-28.57) | 0.35% (0.23-0.48) |
| Iran (Islamic Republic of) | 11.44 (9.38-13.85) | 30.19 (18.75-34.55) | 3.37% (3.19-3.54) | 0.42 (0.36-0.53) | 0.49 (0.36-0.53) | 0.86% (0.47-1.25) | 10.41 (8.96-12.29) | 13.16 (8.93-14.62) | 1.19% (0.85-1.53) |
| Iraq | 12.85 (9.41-17.15) | 32.6 (22.45-45.18) | 3.93% (3.51-4.35) | 0.53 (0.41-0.78) | 0.7 (0.54-0.86) | 1.25% (0.95-1.55) | 14.74 (11.41-20.47) | 19.13 (14.14-24.62) | 1.17% (0.9-1.43) |
| Ireland | 19.31 (16.92-21.65) | 30.35 (22.23-40.42) | 2.17% (1.91-2.43) | 0.6 (0.54-0.65) | 0.41 (0.35-0.46) | -0.97% (-1.23--0.72) | 14.36 (13.02-15.5) | 10.51 (8.78-12.33) | -0.72% (-0.94--0.51) |
| Israel | 18.16 (16.11-20.49) | 34.09 (25.39-44.64) | 2.06% (1.58-2.54) | 0.8 (0.73-0.88) | 0.73 (0.62-0.81) | -0.63% (-0.87--0.38) | 17.95 (16.52-19.66) | 16.65 (14.51-18.81) | -0.53% (-0.81--0.26) |
| Italy | 56.26 (50.89-61.13) | 55.45 (44.12-68.64) | -0.29% (-0.55--0.03) | 0.73 (0.68-0.76) | 0.47 (0.42-0.51) | -1.64% (-1.69--1.59) | 20.14 (18.17-21.55) | 13.33 (11.62-15.11) | -1.6% (-1.67--1.53) |
| Jamaica | 7.88 (6.85-9.1) | 21.21 (15.71-28.21) | 3.55% (3.01-4.09) | 0.4 (0.37-0.44) | 0.66 (0.52-0.82) | 1.92% (1.62-2.22) | 10.22 (9.3-11.25) | 17.72 (13.65-22.27) | 2.06% (1.73-2.41) |
| Japan | 28.75 (27.05-30.64) | 35.51 (29.33-42.99) | 0.91% (0.65-1.16) | 0.63 (0.58-0.66) | 0.49 (0.39-0.54) | -0.95% (-1.03--0.88) | 14.19 (13.33-15.04) | 10.96 (9.64-12.28) | -0.91% (-1.01--0.82) |
| Jordan | 19.97 (15.21-25.82) | 29.21 (22.66-38.04) | 1.13% (0.89-1.37) | 0.82 (0.66-1.04) | 0.58 (0.47-0.73) | -1.48% (-1.65--1.3) | 20.61 (16.9-25.73) | 14.39 (11.71-18.22) | -1.62% (-1.85--1.39) |
| Kazakhstan | 22.16 (18.57-27.84) | 23.6 (19.37-28.71) | -0.34% (-0.72-0.05) | 0.89 (0.73-1.07) | 0.58 (0.49-0.68) | -2.35% (-2.65--2.04) | 26.83 (21.46-32.64) | 16.32 (13.77-19.41) | -2.72% (-3.06--2.39) |
| Kenya | 2.14 (1.62-3.18) | 4.39 (3.16-6.27) | 2.74% (2.54-2.94) | 0.2 (0.17-0.27) | 0.31 (0.24-0.42) | 2% (1.74-2.26) | 5.61 (4.48-7.69) | 8.28 (6.24-11.3) | 1.93% (1.66-2.19) |
| Kiribati | 2.47 (1.84-3.48) | 3.09 (2.24-4.49) | 0.47% (0.29-0.65) | 0.41 (0.32-0.65) | 0.38 (0.29-0.54) | -0.41% (-0.49--0.32) | 9.95 (7.76-14.39) | 8.72 (6.73-12.28) | -0.63% (-0.71--0.54) |
| Kuwait | 26.25 (22.65-29.95) | 31.25 (25.29-38.88) | 0.43% (0-0.87) | 0.68 (0.57-0.77) | 0.56 (0.45-0.67) | -0.51% (-0.84--0.18) | 16.09 (13.94-18) | 12.7 (10.41-15.31) | -0.73% (-1.09--0.38) |
| Kyrgyzstan | 9.94 (8.3-11.65) | 11.18 (9.05-13.56) | 0.93% (0.6-1.28) | 0.52 (0.46-0.58) | 0.4 (0.35-0.47) | -0.59% (-0.76--0.42) | 14.43 (12.81-16.22) | 10.56 (8.99-12.44) | -0.85% (-1.01--0.69) |
| Lao People's Democratic Republic | 9.29 (4.56-14.76) | 15.56 (9.29-22.83) | 1.63% (1.53-1.73) | 1.33 (0.86-1.82) | 1.11 (0.75-1.45) | -0.81% (-0.88--0.74) | 35.89 (21.74-51.56) | 27.73 (18.23-36.56) | -1.1% (-1.18--1.03) |
| Latvia | 18.86 (16.72-21.4) | 28.49 (21.46-37.43) | 1.31% (1-1.62) | 0.53 (0.48-0.58) | 0.63 (0.5-0.77) | 0.29% (-0.11-0.7) | 14.7 (13.45-16.24) | 15.88 (12.58-19.87) | -0.15% (-0.54-0.24) |
| Lebanon | 25.36 (18.2-34) | 68.5 (48.1-93.3) | 3.83% (3.67-3.99) | 0.7 (0.56-0.99) | 0.67 (0.52-0.88) | 0.05% (-0.08-0.19) | 19.25 (15.17-24.53) | 20.19 (15.5-25.89) | 0.4% (0.21-0.59) |
| Lesotho | 2.55 (1.77-3.5) | 5.11 (2.9-7.78) | 3.22% (2.77-3.66) | 0.41 (0.28-0.53) | 0.64 (0.4-0.87) | 2.38% (2.07-2.69) | 9.73 (7-12.69) | 15.88 (9.99-22.06) | 2.56% (2.22-2.9) |
| Liberia | 1.77 (1.02-2.42) | 2.56 (1.59-3.66) | 1.41% (1.16-1.66) | 0.31 (0.19-0.39) | 0.27 (0.17-0.36) | -0.37% (-0.53--0.21) | 7.77 (4.91-9.97) | 6.71 (4.45-9.06) | -0.46% (-0.64--0.27) |
| Libya | 16.88 (11.6-22.91) | 29.92 (20.33-42) | 2.37% (2.01-2.73) | 0.48 (0.36-0.68) | 0.48 (0.36-0.65) | 0.11% (-0.05-0.28) | 13.39 (10-17.65) | 13.89 (10.24-18.73) | 0.29% (0.11-0.47) |
| Lithuania | 25.99 (22.58-29.56) | 28.76 (21.65-37.64) | -0.16% (-0.67-0.35) | 0.52 (0.48-0.56) | 0.47 (0.38-0.58) | -1.11% (-1.59--0.62) | 15.22 (13.8-16.65) | 12.97 (10.19-16.29) | -1.35% (-1.86--0.83) |
| Luxembourg | 32.5 (28.74-36.05) | 35.11 (27.98-44.31) | 0.01% (-0.33-0.34) | 1.03 (0.95-1.11) | 0.57 (0.49-0.68) | -2.24% (-2.31--2.16) | 24.8 (22.6-26.83) | 14.46 (12.16-17.61) | -2.1% (-2.2--2) |
| Madagascar | 5.66 (4.14-7.76) | 7.76 (5.36-11) | 1.26% (1.03-1.49) | 0.54 (0.45-0.67) | 0.66 (0.48-0.9) | 0.86% (0.78-0.94) | 16.9 (13.84-20.93) | 18.71 (13.54-25.41) | 0.56% (0.45-0.66) |
| Malawi | 6.45 (4.74-8.75) | 9.03 (5.57-14.1) | 1.46% (1.31-1.61) | 0.65 (0.53-0.79) | 0.69 (0.51-0.91) | 0.34% (0.24-0.45) | 19.21 (15.36-23.77) | 19.22 (13.32-26.74) | 0.06% (-0.05-0.16) |
| Malaysia | 17.09 (13.26-21.05) | 34.64 (25.42-45.81) | 2.37% (2.25-2.5) | 0.96 (0.83-1.16) | 0.93 (0.71-1.19) | -0.36% (-0.56--0.16) | 24.65 (20.96-28.94) | 23.1 (17.83-29.86) | -0.42% (-0.53--0.31) |
| Maldives | 11.59 (7.13-16.6) | 23.32 (18.53-29.4) | 2.4% (2.16-2.64) | 1.34 (0.92-1.75) | 0.87 (0.68-1.07) | -1.98% (-2.15--1.81) | 30.25 (20.22-39.8) | 18.32 (14.73-22.41) | -2.21% (-2.44--1.98) |
| Mali | 2.76 (2.16-3.52) | 4.07 (2.68-6) | 1.15% (0.98-1.33) | 0.36 (0.29-0.47) | 0.32 (0.25-0.44) | -0.4% (-0.45--0.34) | 10.09 (8.32-12.48) | 9.14 (6.79-12.45) | -0.45% (-0.52--0.37) |
| Malta | 22.96 (20.3-26.64) | 32.03 (25.84-39.18) | 1.16% (0.79-1.53) | 0.81 (0.73-0.89) | 0.51 (0.44-0.59) | -1.53% (-1.66--1.39) | 18.52 (17.01-20.25) | 12.97 (11-15.16) | -1.21% (-1.38--1.03) |
| Marshall Islands | 8.12 (6.36-11.05) | 13.81 (9.49-20.22) | 1.73% (1.59-1.86) | 0.94 (0.75-1.25) | 0.99 (0.72-1.3) | 0.09% (-0.06-0.24) | 22.69 (18.31-30.15) | 24.95 (17.98-33.82) | 0.24% (0.07-0.42) |
| Mauritania | 2.24 (1.35-3.01) | 2.98 (1.89-4.42) | 0.95% (0.82-1.07) | 0.36 (0.23-0.45) | 0.25 (0.17-0.33) | -1.18% (-1.25--1.11) | 9.04 (5.89-11.48) | 6.03 (4.07-8.16) | -1.35% (-1.43--1.26) |
| Mauritius | 10.26 (9.12-11.49) | 15.28 (11.75-19.31) | 1.52% (1.18-1.85) | 0.45 (0.42-0.49) | 0.37 (0.3-0.45) | -0.22% (-0.55-0.11) | 11.3 (10.4-12.22) | 9.64 (7.77-11.86) | -0.12% (-0.45-0.22) |
| Mexico | 8.96 (8.65-9.41) | 20.39 (17.01-24.19) | 2.69% (2.51-2.87) | 0.78 (0.73-0.8) | 0.83 (0.71-0.95) | 0.23% (0.12-0.34) | 18.11 (17.4-18.67) | 19.95 (17.27-22.96) | 0.33% (0.22-0.44) |
| Micronesia (Federated States of) | 9.64 (6.72-13.56) | 18.33 (11.19-26.63) | 2.07% (1.88-2.26) | 1.06 (0.82-1.39) | 1.14 (0.82-1.49) | 0.17% (0.12-0.22) | 26.29 (20.05-34.51) | 28.04 (19.6-37.71) | 0.1% (0.03-0.17) |
| Monaco | 39.39 (29.64-50.25) | 54.31 (40.25-72.87) | 1.3% (1.16-1.44) | 0.85 (0.65-1.05) | 0.79 (0.62-0.96) | -0.11% (-0.16--0.06) | 22.06 (17.06-27.79) | 21.44 (16.49-27.34) | 0.02% (-0.04-0.07) |
| Mongolia | 3.72 (2.65-4.84) | 9.07 (4.92-12.68) | 3.68% (3.36-4) | 0.49 (0.24-0.62) | 0.58 (0.22-0.79) | 0.52% (0.24-0.8) | 12.76 (6.79-15.97) | 15.02 (6.37-20.31) | 0.47% (0.21-0.72) |
| Montenegro | 26.16 (22.15-31.21) | 35.51 (28.74-44.43) | 1.24% (1.16-1.32) | 0.69 (0.6-0.79) | 0.67 (0.55-0.8) | -0.12% (-0.25-0.01) | 18.59 (16.11-21.45) | 17.84 (14.59-21.56) | -0.22% (-0.37--0.06) |
| Morocco | 14.66 (10.36-19.04) | 31.88 (21.41-46.6) | 2.61% (2.55-2.67) | 0.7 (0.51-0.94) | 0.74 (0.56-1.01) | 0.2% (0.13-0.27) | 19.72 (15.5-23.89) | 20.72 (15.48-27.62) | 0.13% (0.09-0.18) |
| Mozambique | 4.1 (2.88-5.81) | 9.78 (5.94-14.98) | 3.38% (3.16-3.59) | 0.64 (0.5-0.83) | 0.96 (0.68-1.33) | 1.84% (1.65-2.02) | 17.77 (13.46-23.35) | 25.98 (18.04-36.46) | 1.77% (1.55-1.98) |
| Myanmar | 10.54 (5.71-15.99) | 17.84 (11.95-24.42) | 1.61% (1.49-1.74) | 1.13 (0.77-1.51) | 1.03 (0.79-1.27) | -0.44% (-0.59--0.28) | 30.69 (19.46-42.55) | 25.52 (18.89-32.17) | -0.81% (-0.98--0.65) |
| Namibia | 3.47 (2.28-4.82) | 8.14 (5.19-12.34) | 3.14% (2.94-3.34) | 0.38 (0.29-0.53) | 0.46 (0.35-0.62) | 0.7% (0.52-0.88) | 10.49 (7.8-13.63) | 12.43 (8.88-17.03) | 0.62% (0.4-0.83) |
| Nauru | 16.41 (11.64-22.22) | 27.2 (17.33-38.26) | 1.37% (1.2-1.54) | 1.02 (0.8-1.25) | 1.12 (0.79-1.43) | 0.25% (-0.01-0.52) | 26.01 (19.71-32.91) | 29.47 (20.21-38.39) | 0.31% (0.04-0.59) |
| Nepal | 5.05 (3.26-7.61) | 12.17 (8.24-17.14) | 3.02% (2.87-3.17) | 0.56 (0.42-0.75) | 0.7 (0.53-0.93) | 0.86% (0.68-1.03) | 16.48 (12.26-22.08) | 18.69 (13.92-25.09) | 0.47% (0.25-0.68) |
| Netherlands | 20.26 (18.15-22.5) | 31.54 (23.38-41.47) | 1.76% (1.61-1.91) | 0.5 (0.46-0.54) | 0.46 (0.39-0.52) | -0.18% (-0.31--0.05) | 12.11 (10.99-13.02) | 11.42 (9.54-13.05) | -0.13% (-0.24--0.02) |
| New Zealand | 10.09 (8.85-11.36) | 18.87 (14.58-23.9) | 2.25% (2.02-2.48) | 0.52 (0.47-0.59) | 0.64 (0.53-0.7) | 0.76% (0.56-0.95) | 12.86 (11.88-14.64) | 16.3 (13.25-17.95) | 0.82% (0.61-1.02) |
| Nicaragua | 5 (4.16-6.6) | 16.3 (12.07-20.93) | 4.91% (4.61-5.2) | 0.43 (0.37-0.54) | 0.79 (0.63-0.94) | 2.16% (1.65-2.67) | 10.63 (9.3-13.67) | 17.5 (13.7-21.23) | 1.88% (1.53-2.23) |
| Niger | 0.23 (0.17-0.31) | 0.37 (0.25-0.56) | 1.84% (1.74-1.95) | 0.04 (0.03-0.06) | 0.04 (0.03-0.06) | -0.22% (-0.28--0.16) | 1.12 (0.87-1.51) | 1.11 (0.8-1.62) | -0.1% (-0.18--0.01) |
| Nigeria | 1.09 (0.8-1.45) | 2 (1.38-2.78) | 2.42% (2.19-2.66) | 0.21 (0.15-0.26) | 0.22 (0.16-0.28) | 0.25% (0.19-0.31) | 4.9 (3.73-6.08) | 5.07 (3.73-6.36) | 0.2% (0.14-0.26) |
| Niue | 15.4 (11.09-20.99) | 26.83 (17.68-38.23) | 1.79% (1.52-2.06) | 0.81 (0.64-1.02) | 0.81 (0.6-1.03) | -0.15% (-0.29-0) | 19.59 (15.17-25.05) | 20.34 (14.86-26.6) | -0.06% (-0.22-0.1) |
| North Macedonia | 13.09 (7.79-15.38) | 19.08 (13.58-25.8) | 0.7% (0.32-1.08) | 0.56 (0.42-0.63) | 0.43 (0.34-0.53) | -1.58% (-1.9--1.25) | 15.4 (10.64-17.3) | 11.41 (8.67-14.61) | -1.79% (-2.13--1.44) |
| Northern Mariana Islands | 13.08 (9.58-18.91) | 30.29 (21.13-39.78) | 2.83% (2.48-3.18) | 0.54 (0.43-0.72) | 0.85 (0.56-1.03) | 2.17% (1.9-2.43) | 12.34 (9.6-17.17) | 21 (13.96-26.04) | 2.39% (2.15-2.63) |
| Norway | 21.77 (19.91-23.97) | 31.6 (26.07-38.07) | 1.58% (1.32-1.84) | 0.54 (0.51-0.57) | 0.49 (0.43-0.53) | -0.2% (-0.29--0.11) | 13.37 (12.73-14.24) | 11.72 (10.55-12.99) | -0.27% (-0.38--0.16) |
| Oman | 10.88 (7.79-14.99) | 26.47 (20.14-32.74) | 3.28% (2.67-3.89) | 0.43 (0.32-0.6) | 0.49 (0.41-0.57) | 1.09% (0.83-1.34) | 10.64 (7.8-14.29) | 11.85 (9.68-14.02) | 0.92% (0.6-1.23) |
| Pakistan | 8.08 (6.63-9.88) | 19.41 (14.17-26.2) | 3.13% (2.97-3.3) | 0.82 (0.71-1.01) | 1.08 (0.9-1.32) | 0.88% (0.6-1.17) | 23.94 (20.77-28.23) | 32.29 (26.35-39.3) | 0.93% (0.64-1.23) |
| Palau | 28.68 (20.64-39.06) | 45.23 (32.19-59.61) | 1.38% (1.2-1.56) | 1.32 (1.02-1.7) | 1.33 (1.03-1.65) | -0.04% (-0.09-0.02) | 33.39 (25.51-43.54) | 35.32 (27.04-44.94) | 0.15% (0.07-0.23) |
| Palestine | 16.14 (10.54-23.24) | 26.33 (18.46-32.91) | 1.5% (0.97-2.05) | 0.73 (0.52-0.95) | 0.73 (0.5-0.89) | -0.08% (-0.33-0.16) | 17.07 (11.98-23.06) | 16.59 (11.76-20.3) | -0.14% (-0.41-0.13) |
| Panama | 12.14 (10.51-14.03) | 21.56 (15.63-28.69) | 1.92% (1.76-2.07) | 0.56 (0.51-0.62) | 0.59 (0.45-0.75) | 0.59% (0.41-0.76) | 14.99 (13.67-16.5) | 15.67 (11.89-20.25) | 0.54% (0.34-0.74) |
| Papua New Guinea | 4.07 (2.84-5.99) | 6.77 (4.44-10.57) | 1.57% (1.41-1.73) | 0.55 (0.4-0.8) | 0.65 (0.46-0.97) | 0.63% (0.51-0.75) | 13.48 (9.96-20.01) | 15.79 (11.1-24.07) | 0.6% (0.48-0.71) |
| Paraguay | 7.63 (6.28-9.21) | 19.46 (13.44-26.8) | 3.09% (2.92-3.27) | 0.62 (0.51-0.72) | 0.85 (0.59-1.11) | 1.26% (1.08-1.43) | 15 (12.62-17.44) | 20.98 (14.56-27.73) | 1.25% (1.07-1.43) |
| Peru | 9.57 (7.63-11.89) | 23.27 (16.04-32.06) | 3.31% (3.04-3.57) | 0.88 (0.74-1.04) | 0.83 (0.6-1.1) | -0.19% (-0.37--0.01) | 22.39 (18.61-26.58) | 20.33 (14.58-27.34) | -0.32% (-0.49--0.14) |
| Philippines | 25.04 (18.35-30.63) | 29.61 (22.63-38.56) | 0.26% (0.02-0.51) | 1.53 (1.29-1.83) | 1.34 (1.08-1.63) | -0.53% (-0.74--0.31) | 39.07 (31.06-46.48) | 34.27 (27.58-41.26) | -0.56% (-0.76--0.35) |
| Poland | 28.85 (25.76-31.26) | 25.89 (20.38-33.34) | -0.72% (-0.98--0.47) | 1.13 (0.98-1.17) | 0.52 (0.43-0.65) | -3.15% (-3.51--2.78) | 30.3 (25.59-31.64) | 13.25 (11.1-17.28) | -3.34% (-3.76--2.92) |
| Portugal | 27.34 (24.04-30.58) | 34.39 (25.19-45.18) | 0.32% (0.06-0.58) | 0.94 (0.84-1.01) | 0.46 (0.4-0.51) | -2.69% (-3.07--2.3) | 19.79 (18-21.23) | 11.62 (10.09-13.3) | -2.08% (-2.37--1.8) |
| Puerto Rico | 18.87 (16.45-21.19) | 20 (14.75-27) | 0.61% (0.3-0.92) | 0.67 (0.59-0.73) | 0.39 (0.3-0.49) | -1.66% (-2.04--1.28) | 16.85 (15.1-18.39) | 10.46 (8.01-13.62) | -1.41% (-1.8--1.02) |
| Qatar | 10.79 (8.42-14.11) | 22.54 (16.34-30.84) | 3.01% (2.52-3.52) | 0.69 (0.55-0.88) | 0.58 (0.44-0.75) | -0.4% (-0.69--0.12) | 14.24 (11.49-17.78) | 11.47 (8.71-15.23) | -0.64% (-0.95--0.32) |
| Republic of Korea | 13.25 (10.51-28.13) | 57.73 (37.52-74.76) | 6.39% (4.39-8.42) | 0.55 (0.44-1.24) | 0.68 (0.5-0.79) | 1.08% (0.01-2.17) | 12.35 (10.19-26.87) | 16.56 (11.45-19.76) | 1.78% (0.47-3.11) |
| Republic of Moldova | 13.63 (11.83-15.38) | 20.15 (16.24-24.43) | 1.66% (1.42-1.9) | 0.46 (0.43-0.51) | 0.42 (0.35-0.49) | -0.31% (-0.57--0.05) | 13.85 (12.19-15.32) | 12.75 (10.6-15.21) | -0.17% (-0.45-0.12) |
| Romania | 13.76 (12.13-15.4) | 23.54 (18.2-29.63) | 1.52% (1.29-1.76) | 0.53 (0.49-0.56) | 0.45 (0.37-0.55) | -0.89% (-1.11--0.66) | 15.19 (13.66-16.24) | 12.94 (10.35-16) | -1.01% (-1.26--0.76) |
| Russian Federation | 17.64 (16.76-19.42) | 36.58 (30.07-44.2) | 3.06% (2.7-3.42) | 0.53 (0.5-0.57) | 0.56 (0.48-0.64) | -0.03% (-0.37-0.31) | 13.83 (13.04-15.05) | 15.7 (13.5-18.17) | 0.22% (-0.13-0.57) |
| Rwanda | 6.32 (3.53-10.06) | 10.97 (7.45-15.58) | 2.12% (1.78-2.47) | 0.85 (0.61-1.19) | 0.8 (0.62-1) | -0.61% (-0.79--0.43) | 25.17 (16.84-36.37) | 21.3 (16.08-27.79) | -1.09% (-1.32--0.87) |
| Saint Kitts and Nevis | 19.83 (17.33-22.64) | 23.81 (15.34-32.1) | 0.42% (0.21-0.62) | 1 (0.91-1.11) | 0.93 (0.78-1.1) | -0.13% (-0.37-0.12) | 26.58 (23.93-29.43) | 24.16 (18.67-29.65) | -0.35% (-0.61--0.09) |
| Saint Lucia | 16.85 (14.63-19.16) | 22.42 (18.16-27.61) | 0.92% (0.65-1.2) | 1 (0.91-1.09) | 0.74 (0.63-0.86) | -1.33% (-1.75--0.91) | 24.79 (22.51-27.13) | 18.92 (15.84-22.49) | -1.09% (-1.49--0.69) |
| Saint Vincent and the Grenadines | 16.34 (14.22-18.63) | 23.1 (18.94-27.81) | 1.16% (0.99-1.34) | 0.96 (0.88-1.05) | 0.97 (0.84-1.11) | 0.24% (-0.15-0.63) | 23.92 (21.66-26.16) | 24.84 (21.11-28.93) | 0.23% (-0.1-0.57) |
| Samoa | 17.01 (11.87-23.83) | 24.89 (14.84-36.57) | 0.92% (0.63-1.21) | 0.85 (0.67-1.28) | 0.75 (0.55-1.09) | -0.63% (-0.68--0.57) | 23.54 (18.38-31.17) | 21.26 (15.12-28.52) | -0.53% (-0.61--0.46) |
| San Marino | 38.91 (30.89-46.89) | 53.61 (38.52-75.07) | 1.15% (1.08-1.23) | 1.07 (0.84-1.3) | 0.9 (0.59-1.33) | -0.48% (-0.58--0.39) | 24.45 (19.4-29.72) | 21.88 (14.13-32.84) | -0.33% (-0.4--0.25) |
| Sao Tome and Principe | 1.7 (1.07-2.4) | 3.82 (2.57-5.53) | 2.99% (2.82-3.15) | 0.16 (0.12-0.25) | 0.22 (0.16-0.34) | 1.11% (1-1.22) | 5.9 (4.1-7.67) | 7.41 (5.23-9.92) | 0.92% (0.78-1.06) |
| Saudi Arabia | 9.97 (6.99-15.08) | 50.55 (36.23-67.54) | 6.45% (6.12-6.78) | 0.51 (0.37-0.85) | 0.66 (0.52-0.81) | 1.06% (0.7-1.41) | 12.24 (8.87-19.66) | 18.92 (14.49-24) | 1.8% (1.52-2.09) |
| Senegal | 1.95 (1.15-2.68) | 2.5 (1.59-3.49) | 0.87% (0.64-1.09) | 0.3 (0.19-0.38) | 0.28 (0.18-0.35) | -0.24% (-0.36--0.11) | 7.61 (4.7-9.7) | 6.75 (4.55-8.79) | -0.34% (-0.5--0.17) |
| Serbia | 10.97 (8.94-12.95) | 20.25 (15.11-26.83) | 2.11% (1.95-2.26) | 0.56 (0.47-0.65) | 0.54 (0.42-0.68) | -0.15% (-0.29--0.01) | 14.4 (12.18-16.54) | 13.83 (10.44-17.95) | -0.28% (-0.43--0.12) |
| Seychelles | 5.95 (4.95-7.07) | 9.74 (8.03-11.91) | 1.21% (0.9-1.53) | 0.4 (0.31-0.47) | 0.37 (0.31-0.43) | -0.86% (-1.13--0.59) | 9.64 (7.96-10.99) | 8.56 (7.27-9.93) | -0.95% (-1.2--0.7) |
| Sierra Leone | 1.58 (0.9-2.25) | 2.4 (1.43-3.42) | 1.54% (1.38-1.71) | 0.27 (0.17-0.35) | 0.26 (0.16-0.33) | -0.08% (-0.13--0.02) | 6.75 (4.13-8.81) | 6.45 (4-8.61) | -0.04% (-0.1-0.01) |
| Singapore | 29.33 (26.08-33.36) | 31.55 (23.94-40.48) | 0.41% (0.04-0.79) | 0.78 (0.71-0.86) | 0.44 (0.37-0.5) | -1.95% (-2.04--1.86) | 18.64 (17.02-20.28) | 10.43 (8.95-12.38) | -1.97% (-2.1--1.84) |
| Slovakia | 20.44 (16.93-23.36) | 24.75 (18.7-32.78) | 0.49% (0.37-0.61) | 0.64 (0.55-0.71) | 0.45 (0.35-0.58) | -1.5% (-1.6--1.39) | 17.13 (14.65-18.88) | 11.61 (8.95-14.92) | -1.6% (-1.72--1.49) |
| Slovenia | 22.88 (16.65-30.19) | 24.44 (18.03-32.71) | 0.3% (0.17-0.44) | 0.69 (0.52-0.88) | 0.44 (0.33-0.58) | -1.69% (-1.78--1.6) | 17.13 (12.8-22.49) | 10.9 (8.08-14.32) | -1.68% (-1.78--1.59) |
| Solomon Islands | 10.78 (5.7-16.84) | 21.24 (11.71-34.11) | 2.12% (1.87-2.37) | 0.93 (0.66-1.37) | 1.14 (0.85-1.6) | 0.71% (0.54-0.88) | 25.72 (17.1-37.94) | 31.26 (21.01-44.12) | 0.66% (0.47-0.85) |
| Somalia | 4.26 (2.75-6.53) | 5.89 (3.67-8.69) | 1.67% (1.46-1.87) | 0.68 (0.49-0.92) | 0.87 (0.6-1.22) | 1.19% (1.07-1.31) | 19.53 (14.1-27.45) | 23.78 (16.32-33.48) | 0.99% (0.88-1.09) |
| South Africa | 5.51 (4.76-6.29) | 5.72 (4.87-7.38) | 0.02% (-0.26-0.3) | 0.4 (0.33-0.47) | 0.4 (0.34-0.45) | -0.08% (-0.42-0.26) | 10.43 (9.26-11.87) | 9.21 (8.11-10.78) | -0.47% (-0.78--0.16) |
| South Sudan | 3.52 (2.29-5.21) | 5.2 (3.17-8.62) | 1.66% (1.43-1.89) | 0.53 (0.39-0.72) | 0.63 (0.44-0.91) | 0.83% (0.7-0.95) | 14.4 (10.23-19.91) | 16.91 (11.14-25.66) | 0.75% (0.6-0.9) |
| Spain | 20.35 (17.97-22.48) | 28.33 (21.33-37.73) | 1.12% (0.83-1.42) | 0.53 (0.49-0.56) | 0.42 (0.36-0.46) | -0.86% (-0.9--0.82) | 13.51 (12.21-14.42) | 10.73 (9.05-12.28) | -0.83% (-0.9--0.77) |
| Sri Lanka | 15.82 (12.24-18.94) | 27.54 (19.14-39.71) | 1.49% (1.16-1.82) | 0.88 (0.74-1.01) | 0.65 (0.48-0.86) | -1.22% (-1.56--0.89) | 22.1 (18.33-25.5) | 16.2 (11.67-22.22) | -1.4% (-1.74--1.05) |
| Sudan | 6.47 (3.63-10.02) | 15.38 (9.46-23.23) | 3.03% (2.87-3.18) | 0.46 (0.31-0.62) | 0.54 (0.39-0.71) | 0.73% (0.59-0.87) | 12.22 (7.98-17.19) | 14.31 (9.77-19.49) | 0.69% (0.56-0.83) |
| Suriname | 7.91 (6.13-9.5) | 13.18 (10.21-16.79) | 1.65% (1.29-2.02) | 0.59 (0.5-0.67) | 0.62 (0.5-0.74) | 0.27% (0-0.54) | 15.21 (12.66-17.38) | 16.22 (13.09-19.6) | 0.25% (-0.01-0.52) |
| Sweden | 15.74 (14.4-17.6) | 19.42 (15.88-23.5) | 1.04% (0.88-1.21) | 0.58 (0.53-0.63) | 0.44 (0.39-0.48) | -0.83% (-1.03--0.63) | 12.77 (11.92-13.76) | 10.05 (9.17-11.01) | -0.57% (-0.76--0.38) |
| Switzerland | 17.46 (15.5-20.35) | 31.45 (23.3-41.78) | 1.65% (0.96-2.34) | 0.38 (0.34-0.45) | 0.5 (0.41-0.56) | 0.62% (0.13-1.11) | 9.48 (8.63-11.43) | 11.87 (9.39-13.8) | 0.45% (-0.02-0.92) |
| Syrian Arab Republic | 3.51 (2.25-4.83) | 7.63 (4.95-10.56) | 2.78% (2.6-2.97) | 0.18 (0.12-0.23) | 0.18 (0.12-0.24) | -0.37% (-0.57--0.18) | 4.61 (3.02-6) | 4.62 (2.99-6.27) | -0.27% (-0.47--0.08) |
| Taiwan (Province of China) | 23.54 (21.21-26.18) | 42.24 (31.3-57.91) | 1.87% (1.48-2.25) | 0.61 (0.57-0.65) | 0.55 (0.42-0.71) | -0.4% (-0.46--0.33) | 15.78 (14.68-16.95) | 14.22 (10.86-18.68) | -0.46% (-0.56--0.35) |
| Tajikistan | 1.82 (1.53-2.23) | 2.51 (1.95-3.21) | 0.94% (0.59-1.3) | 0.2 (0.11-0.28) | 0.24 (0.14-0.31) | 0.73% (0.6-0.86) | 4.77 (3.3-6.26) | 5.35 (3.53-6.75) | 0.29% (0.12-0.47) |
| Thailand | 13.97 (11.51-17.51) | 21.14 (13.94-37.43) | 0.6% (0.04-1.17) | 0.81 (0.69-0.96) | 0.58 (0.43-0.79) | -1.59% (-1.83--1.36) | 19.72 (16.88-23.92) | 13.4 (9.65-20.03) | -1.99% (-2.29--1.69) |
| Timor-Leste | 6.5 (3.88-9.34) | 14.33 (8.19-20.65) | 2.92% (2.65-3.19) | 0.91 (0.65-1.18) | 1 (0.68-1.33) | 0.34% (0.18-0.49) | 23.26 (15.71-30.49) | 24.23 (15.76-32.56) | 0.1% (-0.1-0.29) |
| Togo | 2.42 (1.41-3.26) | 2.84 (1.75-4.05) | 0.56% (0.3-0.83) | 0.3 (0.18-0.38) | 0.28 (0.17-0.37) | -0.35% (-0.39--0.31) | 7.78 (4.78-9.83) | 6.95 (4.49-9.35) | -0.46% (-0.52--0.41) |
| Tokelau | 11.05 (7.35-16.13) | 20.65 (12.79-29.95) | 2.03% (1.9-2.17) | 0.84 (0.65-1.11) | 0.77 (0.57-0.99) | -0.23% (-0.32--0.15) | 20.76 (15.49-28.27) | 19.09 (13.7-25.44) | -0.34% (-0.43--0.26) |
| Tonga | 7.51 (5.61-10.3) | 12.39 (8.59-17.7) | 1.44% (1.13-1.74) | 0.56 (0.44-0.72) | 0.66 (0.5-0.85) | 0.5% (0.25-0.76) | 13.28 (10.39-17.34) | 15.71 (11.66-21.1) | 0.45% (0.18-0.72) |
| Trinidad and Tobago | 10.34 (9.19-11.5) | 14.65 (10.35-19.95) | 0.75% (0.4-1.09) | 0.63 (0.59-0.68) | 0.51 (0.39-0.66) | -1.34% (-1.71--0.97) | 16.56 (15.39-17.81) | 13.9 (10.29-18.24) | -1.23% (-1.6--0.85) |
| Tunisia | 13.98 (10.9-17.75) | 32.01 (21.71-45.98) | 2.98% (2.84-3.11) | 0.4 (0.31-0.55) | 0.41 (0.3-0.56) | 0.14% (0.09-0.19) | 10.69 (8.73-13.35) | 11.69 (8.32-15.98) | 0.32% (0.28-0.37) |
| Trkiye | 14.01 (9.87-18.24) | 29.04 (22.18-38.23) | 2.79% (2.54-3.04) | 0.73 (0.56-0.91) | 0.53 (0.41-0.75) | -1.08% (-1.25--0.91) | 18.71 (14.16-23.02) | 13.45 (10.35-17.88) | -1.24% (-1.36--1.12) |
| Turkmenistan | 7.13 (6.26-8.08) | 12.59 (9.71-16.32) | 3.1% (2.4-3.8) | 0.43 (0.39-0.47) | 0.4 (0.32-0.5) | -0.05% (-0.53-0.42) | 12.14 (11.02-13.34) | 12.05 (9.6-15.28) | 0.34% (-0.19-0.88) |
| Tuvalu | 10.54 (7.39-14.94) | 16.43 (11.03-23.37) | 1.24% (1.04-1.45) | 0.98 (0.76-1.29) | 0.9 (0.65-1.19) | -0.41% (-0.48--0.33) | 24.47 (18.68-33.02) | 22.2 (15.69-30.22) | -0.41% (-0.48--0.34) |
| Uganda | 3.46 (2.33-4.98) | 11.39 (7.4-16.06) | 4.41% (4.11-4.72) | 0.48 (0.37-0.66) | 0.89 (0.64-1.09) | 2.48% (2.24-2.72) | 12.9 (9.71-17.82) | 24.32 (17.23-31.07) | 2.38% (2.12-2.65) |
| Ukraine | 15.81 (13.13-20.64) | 32.02 (25.29-39.82) | 2.67% (2.42-2.93) | 0.46 (0.4-0.56) | 0.53 (0.45-0.64) | 0.18% (-0.03-0.39) | 12.59 (10.9-15.76) | 16.55 (13.95-19.59) | 0.59% (0.35-0.84) |
| United Arab Emirates | 14.2 (8.27-19.98) | 29.94 (16.08-45.83) | 2.42% (2.16-2.67) | 0.79 (0.35-1.17) | 0.82 (0.41-1.23) | 0.12% (-0.3-0.55) | 19.69 (9.7-28.3) | 22.6 (11.68-34.23) | 0.48% (0.18-0.77) |
| United Kingdom | 16.81 (15.8-17.56) | 23.57 (19.34-28.33) | 1.37% (1.29-1.46) | 0.45 (0.42-0.47) | 0.39 (0.36-0.41) | -0.52% (-0.61--0.42) | 10.92 (10.24-11.56) | 9.63 (8.99-10.46) | -0.4% (-0.49--0.31) |
| United Republic of Tanzania | 5.92 (4.24-8.1) | 11.09 (7.73-15.8) | 2.48% (2.22-2.73) | 0.64 (0.52-0.81) | 0.82 (0.63-1.03) | 1.11% (0.98-1.23) | 17.84 (13.99-22.77) | 22.09 (16.55-28.8) | 1% (0.86-1.14) |
| United States of America | 34.21 (32.99-35.29) | 47.8 (40.83-55.94) | 1.15% (0.97-1.33) | 0.39 (0.36-0.4) | 0.44 (0.4-0.46) | 0.42% (0.36-0.48) | 11.02 (10.21-11.87) | 12.4 (11.25-13.55) | 0.4% (0.31-0.48) |
| United States Virgin Islands | 8.98 (7.17-11.05) | 14.82 (11.13-19.05) | 2.16% (1.9-2.42) | 0.47 (0.39-0.56) | 0.56 (0.46-0.66) | 1.08% (0.83-1.33) | 12.28 (10.06-14.67) | 14.56 (11.65-17.69) | 1.03% (0.82-1.24) |
| Uruguay | 8.86 (7.65-10.46) | 20.33 (15.16-26.67) | 2.47% (1.87-3.07) | 0.42 (0.38-0.49) | 0.56 (0.5-0.61) | 0.5% (0.07-0.94) | 10.46 (9.39-12.12) | 14.25 (12.82-15.69) | 0.65% (0.19-1.1) |
| Uzbekistan | 2.02 (1.66-2.86) | 6.26 (5.03-7.65) | 4.09% (3.84-4.34) | 0.15 (0.12-0.26) | 0.31 (0.26-0.37) | 2.6% (2.32-2.87) | 3.72 (3.01-5.82) | 7.42 (6.07-8.77) | 2.42% (2.21-2.62) |
| Vanuatu | 5.78 (3.69-8.7) | 10.1 (6.27-14.93) | 1.42% (1.15-1.69) | 0.7 (0.49-1) | 0.85 (0.59-1.17) | 0.54% (0.42-0.65) | 16.99 (11.97-25.04) | 21.25 (14.47-29.9) | 0.55% (0.41-0.69) |
| Venezuela (Bolivarian Republic of) | 6.68 (5.98-7.45) | 17.74 (12.82-24.09) | 3.26% (2.9-3.61) | 0.51 (0.46-0.54) | 0.65 (0.49-0.84) | 0.61% (0.46-0.77) | 12.39 (11.51-13.3) | 16.12 (12.11-21.23) | 0.67% (0.53-0.8) |
| Viet Nam | 14.6 (10.49-19.72) | 57.61 (35.62-77.76) | 5.97% (5.47-6.48) | 0.93 (0.69-1.58) | 1.34 (1.04-1.69) | 2.09% (1.73-2.45) | 24.16 (18.03-36.08) | 36.32 (27.08-46.92) | 2.33% (1.92-2.74) |
| Yemen | 6.23 (3.56-9.84) | 13.26 (8.36-19.62) | 3.25% (3.04-3.46) | 0.49 (0.34-0.71) | 0.54 (0.4-0.73) | 0.5% (0.39-0.61) | 12.4 (8.27-17.87) | 14.19 (10.12-19.22) | 0.63% (0.52-0.74) |
| Zambia | 6.41 (4.24-9.46) | 13.43 (9.07-19.39) | 2.81% (2.54-3.08) | 0.73 (0.57-0.95) | 0.89 (0.68-1.14) | 0.72% (0.62-0.81) | 21.39 (15.93-28.78) | 25.19 (18.52-33.21) | 0.53% (0.44-0.63) |
| Zimbabwe | 9.15 (6.42-11.88) | 13.09 (7.37-18.92) | 1.54% (1.23-1.85) | 0.8 (0.67-0.95) | 1.03 (0.73-1.36) | 1.5% (1.2-1.79) | 20.54 (16.3-24.8) | 28.34 (18.73-38.35) | 1.86% (1.51-2.21) |

Abbreviations: CI=confidence interval. DALY=disability adjusted life year. EAPC=estimated annual percentage change. UI=uncertainty interval.

**Supplementary Table S4:** Incident cases, YLLs and YLDs of Thyroid cancer (TC) in 1990 and 2019 and their percentage change from 1990 to 2019 and their percentage change from 1990 to 2019.

|  | **Incident cases** | | | **YLLs** | | | **YLDs** | | |
| --- | --- | --- | --- | --- | --- | --- | --- | --- | --- |
| **Characteristics** | **1990no(95%UI)** | **2019no(95%UI)** | **Percentage**  **change**  **(95%UI))** | **1990no(95%UI)** | **2019no(95%UI)** | **Percentage**  **change**  **(95%UI)** | **1990no(95%UI)** | **2019no(95%UI)** | **Percentage**  **change**  **(95%UI)** |
| Global | 87583 (82236-92717) | 233847 (211637-252807) | 1.67 (1.43-1.92) | 626076 (577031-688376) | 1117973 (1025894-1202740) | 0.79 (0.58-0.95) | 41387 (28516-56049) | 113868 (78726-154536) | 1.75 (1.5-2.01) |
| Sex |  |  |  |  |  |  |  |  |  |
| Male | 23787 (22215-25699) | 76014 (68232-82923) | 2.2 (1.81-2.56) | 217642 (198214-246021) | 472127 (427234-514238) | 1.17 (0.83-1.47) | 11393 (8029-15198) | 37714 (26182-51552) | 2.31 (1.93-2.69) |
| Female | 63796 (58659-68290) | 157833 (140395-173068) | 1.47 (1.22-1.75) | 408433 (361099-457477) | 645846 (564364-705496) | 0.58 (0.37-0.81) | 29994 (20545-40782) | 76154 (51836-105074) | 1.54 (1.27-1.83) |
| **SDI quintile** |  |  |  |  |  |  |  |  |  |
| High SDI | 32896 (31785-33893) | 68405 (62053-74858) | 1.08 (0.9-1.27) | 123150 (117764-127465) | 170437 (154179-179022) | 0.38 (0.27-0.45) | 16354 (11411-22086) | 34445 (23877-46851) | 1.11 (0.92-1.32) |
| High-middle SDI | 25516 (23779-26871) | 57965 (52409-64001) | 1.27 (1.05-1.53) | 158165 (147670-166429) | 212269 (194657-229179) | 0.34 (0.23-0.46) | 12190 (8425-16532) | 28700 (19827-39702) | 1.35 (1.12-1.65) |
| Low SDI | 3872 (2923-5044) | 11680 (9586-13741) | 1.95 (1.27-2.97) | 64119 (48920-83439) | 125636 (103058-146397) | 0.96 (0.52-1.58) | 1605 (972-2382) | 5258 (3457-7571) | 2.28 (1.46-3.64) |
| Low-middle SDI | 8603 (7223-10106) | 30107 (25984-33694) | 2.49 (1.86-3.17) | 115938 (98617-140112) | 254946 (223048-283938) | 1.2 (0.81-1.57) | 3703 (2404-5231) | 13852 (9262-19200) | 2.74 (2.07-3.52) |
| Middle SDI | 16647 (15141-18610) | 65559 (57599-73072) | 2.86 (2.28-3.47) | 164324 (151405-190170) | 353961 (313200-392260) | 1.15 (0.8-1.43) | 7511 (5137-10261) | 31550 (21555-43586) | 3.2 (2.59-3.87) |
| **GBD region** |  |  |  |  |  |  |  |  |  |
| Andean Latin America | 426 (368-517) | 2145 (1659-2688) | 4.03 (2.68-5.53) | 5338 (4668-6425) | 14365 (11162-17655) | 1.69 (0.99-2.43) | 178 (120-260) | 976 (626-1421) | 4.5 (2.97-6.46) |
| Australasia | 519 (479-565) | 1738 (1359-2228) | 2.35 (1.53-3.37) | 2258 (2141-2409) | 4725 (4010-5099) | 1.09 (0.74-1.29) | 257 (177-364) | 867 (557-1289) | 2.37 (1.5-3.57) |
| Caribbean | 486 (448-521) | 1246 (1045-1482) | 1.57 (1.14-2.04) | 4113 (3650-4553) | 8021 (6669-9473) | 0.95 (0.65-1.29) | 221 (150-300) | 590 (391-820) | 1.67 (1.19-2.21) |
| Central Asia | 759 (673-878) | 1467 (1306-1650) | 0.93 (0.67-1.27) | 6457 (5536-7421) | 8512 (7624-9471) | 0.32 (0.15-0.53) | 357 (240-512) | 719 (488-995) | 1.01 (0.69-1.38) |
| Central Europe | 4352 (3970-4532) | 5313 (4618-6120) | 0.22 (0.05-0.48) | 28999 (25463-29873) | 21679 (18928-24957) | -0.25 (-0.35--0.04) | 2066 (1427-2794) | 2611 (1772-3627) | 0.26 (0.09-0.54) |
| Central Latin America | 1798 (1698-1864) | 7183 (6143-8404) | 3 (2.41-3.66) | 18335 (17152-18943) | 45030 (38603-51985) | 1.46 (1.12-1.82) | 787 (546-1062) | 3352 (2313-4634) | 3.26 (2.56-4.01) |
| Central Sub-Saharan Africa | 192 (138-258) | 520 (363-729) | 1.71 (0.97-2.63) | 3402 (2518-4500) | 7166 (5099-9882) | 1.11 (0.57-1.74) | 75 (45-113) | 220 (130-350) | 1.94 (1.08-3.1) |
| East Asia | 11092 (9401-13053) | 41580 (34751-50204) | 2.75 (1.94-3.89) | 104297 (89513-125433) | 176951 (148178-207487) | 0.7 (0.31-1.11) | 5059 (3354-7089) | 20386 (13432-29298) | 3.03 (2.14-4.34) |
| Eastern Europe | 6113 (5750-6870) | 12257 (10669-14146) | 1.01 (0.71-1.31) | 34246 (32191-38031) | 43268 (38430-48636) | 0.26 (0.11-0.42) | 2968 (2048-4183) | 6158 (4143-8691) | 1.07 (0.77-1.43) |
| Eastern Sub-Saharan Africa | 2063 (1400-2886) | 5343 (4114-6792) | 1.59 (0.77-3.07) | 35453 (24025-49414) | 60192 (47613-74621) | 0.7 (0.18-1.57) | 860 (478-1360) | 2406 (1533-3654) | 1.8 (0.82-3.65) |
| High-income Asia Pacific | 6725 (6336-7583) | 15660 (13134-18056) | 1.33 (0.8-1.73) | 24906 (23554-29345) | 41342 (34412-45254) | 0.66 (0.25-0.82) | 3287 (2232-4561) | 7678 (5161-10820) | 1.34 (0.8-1.76) |
| High-income North America | 12626 (12140-13004) | 28296 (24461-32790) | 1.24 (0.93-1.59) | 29950 (28546-30821) | 54376 (51049-56428) | 0.82 (0.74-0.88) | 6585 (4620-8953) | 14673 (9973-19800) | 1.23 (0.92-1.58) |
| North Africa and Middle East | 3882 (3233-4451) | 19253 (15675-22281) | 3.96 (3.15-5.26) | 28477 (23841-33192) | 64576 (54795-74666) | 1.27 (0.88-1.76) | 1847 (1197-2569) | 9605 (6205-13522) | 4.2 (3.32-5.56) |
| Oceania | 54 (42-70) | 162 (114-220) | 1.99 (1.32-2.91) | 621 (498-828) | 1500 (1092-2073) | 1.42 (0.92-2.05) | 24 (15-36) | 76 (46-119) | 2.12 (1.34-3.14) |
| South Asia | 7930 (6672-9896) | 31534 (26591-36439) | 2.98 (1.96-3.89) | 113167 (96137-144929) | 277119 (242233-315800) | 1.45 (0.94-1.92) | 3389 (2238-4882) | 14455 (9658-20222) | 3.27 (2.17-4.33) |
| Southeast Asia | 7200 (5805-8275) | 25581 (20569-29886) | 2.55 (1.95-3.31) | 71624 (59527-80817) | 152068 (126427-174531) | 1.12 (0.8-1.49) | 3237 (2088-4458) | 12235 (8065-17164) | 2.78 (2.11-3.6) |
| Southern Latin America | 959 (882-1023) | 2002 (1561-2562) | 1.09 (0.62-1.71) | 8300 (7533-8712) | 10461 (9762-11216) | 0.26 (0.16-0.44) | 427 (296-591) | 950 (608-1395) | 1.23 (0.66-1.99) |
| Southern Sub-Saharan Africa | 345 (296-380) | 739 (633-859) | 1.14 (0.83-1.55) | 3723 (3260-4085) | 7162 (6027-8269) | 0.92 (0.68-1.22) | 155 (102-213) | 335 (226-471) | 1.16 (0.82-1.63) |
| Tropical Latin America | 1636 (1566-1730) | 4528 (4246-5027) | 1.77 (1.59-2.03) | 16335 (15711-17391) | 29253 (27508-33253) | 0.79 (0.69-0.96) | 726 (509-979) | 2118 (1466-2923) | 1.92 (1.65-2.26) |
| Western Europe | 18052 (16979-18739) | 26217 (22591-30005) | 0.45 (0.26-0.67) | 80117 (74786-82281) | 77259 (70181-81908) | -0.04 (-0.1-0.03) | 8733 (6105-11812) | 12974 (8853-18049) | 0.49 (0.28-0.71) |
| Western Sub-Saharan Africa | 375 (298-437) | 1083 (857-1325) | 1.89 (1.35-2.55) | 5957 (4780-6949) | 12948 (10265-15417) | 1.17 (0.77-1.63) | 150 (98-211) | 484 (306-720) | 2.23 (1.57-3.07) |

Abbreviations: GBD=Global Burden of Disease. SDI=Socio-demographic Index. UI=uncertainty interval. YLDs=years of life lived with disability. YLLs=years of life lost.

**Supplementary Table S5 :**Age-standardised incidence, YLL and YLD rates for Thyroid cancer (TC) in 1990 and 2019 and their temporal trends from 1990 to 2019 and their temporal trends from 1990 to 2019.

|  | **Age-standardised incidence rate per 100000 population** | | | **Age-standardised YLL rate per 100000 population** | | | **Age-standardised YLD rate per 100000 population** | | |
| --- | --- | --- | --- | --- | --- | --- | --- | --- | --- |
| **Characteristics** | **1990no(95%UI)** | **2019no(95%UI)** | **EAPC no(95%UI)** | **1990no(95%UI)** | **2019no(95%UI)** | **EAPC no(95%UI)** | **1990no(95%UI)** | **2019no(95%UI)** | **EAPC no(95%UI)** |
| Global | 2.01 (1.9-2.12) | 2.83 (2.56-3.06) | 1.25% (1.12-1.37) | 14.62 (13.59-15.98) | 13.61 (12.48-14.64) | -0.26% (-0.3--0.23) | 0.94 (0.65-1.26) | 1.37 (0.95-1.87) | 1.39% (1.26-1.53) |
| Sex |  |  |  |  |  |  |  |  |  |
| Male | 1.16 (1.09-1.24) | 1.9 (1.71-2.07) | 1.89% (1.77-2.02) | 10.67 (9.73-12.01) | 11.99 (10.88-13.04) | 0.51% (0.44-0.58) | 0.54 (0.38-0.72) | 0.93 (0.65-1.27) | 2.06% (1.93-2.2) |
| Female | 2.82 (2.61-3.02) | 3.74 (3.32-4.1) | 0.98% (0.85-1.12) | 18.16 (16.18-20.3) | 15.14 (13.21-16.54) | -0.73% (-0.77--0.68) | 1.31 (0.91-1.78) | 1.81 (1.23-2.49) | 1.1% (0.96-1.25) |
| **SDI quintile** |  |  |  |  |  |  |  |  |  |
| High SDI | 3.4 (3.28-3.5) | 4.59 (4.17-5.03) | 1.21% (0.9-1.51) | 12.18 (11.67-12.6) | 9.96 (9.14-10.45) | -0.67% (-0.78--0.56) | 1.69 (1.18-2.29) | 2.33 (1.6-3.22) | 1.26% (0.95-1.57) |
| High-middle SDI | 2.28 (2.13-2.4) | 3.06 (2.76-3.38) | 1.05% (0.94-1.16) | 14.37 (13.43-15.12) | 10.84 (9.94-11.68) | -1.13% (-1.21--1.05) | 1.08 (0.75-1.46) | 1.52 (1.05-2.1) | 1.22% (1.1-1.34) |
| Low SDI | 1.23 (0.98-1.57) | 1.63 (1.34-1.9) | 0.95% (0.9-0.99) | 20.65 (16.41-26.18) | 18.88 (15.5-21.88) | -0.38% (-0.42--0.34) | 0.47 (0.3-0.69) | 0.69 (0.47-0.98) | 1.28% (1.23-1.32) |
| Low-middle SDI | 1.13 (0.97-1.33) | 1.91 (1.66-2.13) | 1.8% (1.74-1.85) | 15.63 (13.51-18.84) | 16.89 (14.81-18.77) | 0.25% (0.17-0.33) | 0.45 (0.3-0.63) | 0.86 (0.58-1.17) | 2.18% (2.12-2.23) |
| Middle SDI | 1.31 (1.2-1.51) | 2.49 (2.19-2.77) | 2.36% (2.28-2.44) | 13.77 (12.74-16.24) | 13.84 (12.31-15.34) | 0.16% (0.09-0.23) | 0.56 (0.38-0.76) | 1.18 (0.81-1.62) | 2.73% (2.65-2.81) |
| **GBD region** |  |  |  |  |  |  |  |  |  |
| Andean Latin America | 1.76 (1.53-2.12) | 3.63 (2.79-4.54) | 2.68% (2.43-2.93) | 23.02 (20.09-27.6) | 25.11 (19.4-30.82) | 0.43% (0.28-0.57) | 0.69 (0.47-1.01) | 1.63 (1.05-2.36) | 3.15% (2.86-3.45) |
| Australasia | 2.31 (2.13-2.52) | 4.44 (3.47-5.72) | 2.72% (2.37-3.07) | 9.84 (9.34-10.48) | 10.71 (9.13-11.53) | 0.67% (0.5-0.83) | 1.15 (0.78-1.62) | 2.23 (1.42-3.33) | 2.76% (2.41-3.12) |
| Caribbean | 1.7 (1.57-1.82) | 2.43 (2.04-2.89) | 1.42% (1.29-1.55) | 14.84 (13.26-16.34) | 15.61 (12.98-18.45) | 0.38% (0.18-0.59) | 0.76 (0.52-1.03) | 1.15 (0.76-1.6) | 1.59% (1.48-1.71) |
| Central Asia | 1.44 (1.27-1.67) | 1.69 (1.51-1.89) | 0.42% (0.11-0.73) | 12.57 (10.86-14.54) | 10.66 (9.59-11.8) | -0.97% (-1.19--0.74) | 0.66 (0.45-0.95) | 0.81 (0.55-1.11) | 0.55% (0.23-0.87) |
| Central Europe | 3.04 (2.77-3.16) | 3.09 (2.67-3.57) | -0.17% (-0.26--0.08) | 19.85 (17.4-20.44) | 11.2 (9.77-12.92) | -2.29% (-2.5--2.09) | 1.44 (0.99-1.94) | 1.53 (1.03-2.15) | -0.01% (-0.1-0.07) |
| Central Latin America | 1.8 (1.69-1.87) | 2.9 (2.49-3.4) | 1.57% (1.49-1.65) | 19.49 (18.11-20.19) | 18.7 (16-21.58) | -0.19% (-0.33--0.05) | 0.75 (0.53-1) | 1.34 (0.93-1.85) | 1.92% (1.83-2.02) |
| Central Sub-Saharan Africa | 0.72 (0.51-0.98) | 0.8 (0.54-1.14) | 0.34% (0.27-0.4) | 12.44 (9.05-16.64) | 11.33 (7.86-16.06) | -0.34% (-0.36--0.32) | 0.25 (0.15-0.38) | 0.3 (0.18-0.49) | 0.59% (0.5-0.68) |
| East Asia | 1.07 (0.92-1.27) | 2.11 (1.77-2.54) | 2.59% (2.45-2.72) | 10.6 (9.17-12.81) | 8.83 (7.38-10.29) | -0.45% (-0.55--0.36) | 0.47 (0.32-0.65) | 1.03 (0.68-1.49) | 2.98% (2.84-3.12) |
| Eastern Europe | 2.28 (2.15-2.56) | 4.25 (3.7-4.93) | 2.44% (2.17-2.7) | 12.46 (11.72-13.8) | 13.67 (12.14-15.36) | 0% (-0.32-0.32) | 1.11 (0.76-1.55) | 2.15 (1.43-3.04) | 2.59% (2.31-2.88) |
| Eastern Sub-Saharan Africa | 1.94 (1.41-2.65) | 2.19 (1.72-2.72) | 0.3% (0.15-0.45) | 33.97 (24.48-46.08) | 26.88 (21.06-32.95) | -0.93% (-1.03--0.84) | 0.75 (0.44-1.14) | 0.9 (0.59-1.35) | 0.52% (0.38-0.66) |
| High-income Asia Pacific | 3.32 (3.13-3.75) | 4.98 (4.19-5.79) | 2.16% (1.38-2.94) | 12.45 (11.74-14.64) | 9.96 (8.68-10.83) | -0.49% (-0.92--0.07) | 1.61 (1.1-2.24) | 2.5 (1.66-3.55) | 2.29% (1.5-3.1) |
| High-income North America | 3.96 (3.82-4.08) | 5.4 (4.64-6.27) | 1.04% (0.85-1.23) | 8.99 (8.58-9.24) | 9.43 (8.95-9.79) | 0.14% (0.06-0.22) | 2.06 (1.44-2.81) | 2.79 (1.88-3.79) | 1.01% (0.82-1.2) |
| North Africa and Middle East | 1.7 (1.44-2) | 3.46 (2.89-3.96) | 2.62% (2.53-2.71) | 13.64 (11.17-17.07) | 13.18 (11.36-15.16) | -0.05% (-0.14-0.05) | 0.78 (0.52-1.08) | 1.7 (1.12-2.38) | 2.88% (2.8-2.97) |
| Oceania | 1.45 (1.15-1.86) | 1.8 (1.3-2.4) | 0.68% (0.57-0.78) | 17.55 (14.19-23.02) | 18.25 (13.56-24.81) | 0.16% (0.09-0.22) | 0.6 (0.39-0.88) | 0.79 (0.48-1.2) | 0.81% (0.69-0.94) |
| South Asia | 1.05 (0.9-1.32) | 1.9 (1.61-2.19) | 2.06% (1.94-2.18) | 15.48 (13.27-19.69) | 17.48 (15.24-19.92) | 0.38% (0.23-0.53) | 0.41 (0.28-0.59) | 0.85 (0.57-1.18) | 2.53% (2.41-2.65) |
| Southeast Asia | 2.25 (1.86-2.55) | 3.72 (3.01-4.32) | 1.76% (1.7-1.82) | 24.19 (20.62-27.34) | 23.61 (19.84-26.9) | -0.04% (-0.1-0.02) | 0.95 (0.63-1.29) | 1.73 (1.15-2.41) | 2.09% (2.02-2.15) |
| Southern Latin America | 2.04 (1.88-2.18) | 2.58 (2-3.31) | 0.67% (0.53-0.81) | 17.7 (16.1-18.58) | 13.01 (12.15-13.94) | -1.16% (-1.36--0.97) | 0.9 (0.63-1.25) | 1.23 (0.79-1.82) | 0.92% (0.79-1.06) |
| Southern Sub-Saharan Africa | 1 (0.85-1.1) | 1.13 (0.97-1.3) | 0.43% (0.35-0.52) | 11.38 (9.97-12.61) | 11.47 (9.71-13.09) | 0.18% (-0.06-0.42) | 0.42 (0.29-0.58) | 0.49 (0.33-0.68) | 0.53% (0.44-0.63) |
| Tropical Latin America | 1.53 (1.46-1.61) | 1.82 (1.71-2.02) | 0.67% (0.41-0.93) | 16.1 (15.46-16.95) | 11.98 (11.24-13.58) | -0.92% (-1.04--0.8) | 0.65 (0.46-0.86) | 0.85 (0.59-1.17) | 0.96% (0.67-1.26) |
| Western Europe | 3.63 (3.41-3.77) | 3.93 (3.4-4.5) | 0.17% (-0.03-0.36) | 14.75 (13.72-15.14) | 9.69 (8.91-10.24) | -1.58% (-1.62--1.54) | 1.78 (1.23-2.41) | 1.97 (1.34-2.77) | 0.25% (0.04-0.46) |
| Western Sub-Saharan Africa | 0.36 (0.28-0.42) | 0.43 (0.34-0.52) | 0.69% (0.64-0.75) | 5.76 (4.52-6.75) | 5.5 (4.27-6.49) | -0.16% (-0.19--0.13) | 0.13 (0.09-0.18) | 0.18 (0.11-0.25) | 1.07% (1-1.14) |

Abbreviations: CI=confidence interval. EAPC=estimated annual percentage change. GBD=Global Burden of Disease. SDI=Socio-demographic Index. UI=uncertainty interval. YLD=years of life lived with disability. YLL=years of life lost.

**Supplementary Table S6:**Incident cases for Thyroid cancer (TC) by 204 countries or territories in 2019

| **Location** | **Incident cases** |
| --- | --- |
| Afghanistan | 439.456702(220.7846953－725.5027668) |
| Albania | 95.98825354(70.28458382－128.1013952) |
| Algeria | 1827.371233(1214.485307－2508.298613) |
| American Samoa | 1.798263898(1.315729238－2.352995746) |
| Andorra | 5.868636082(4.208439745－7.882259787) |
| Angola | 123.3786982(84.85765637－182.516377) |
| Antigua and Barbuda | 3.388038505(2.747205775－4.161310721) |
| Argentina | 1155.251651(885.8590074－1494.050372) |
| Armenia | 91.13075542(74.22613405－109.5756195) |
| Australia | 1580.055781(1204.229441－2072.979512) |
| Austria | 607.6309794(479.5047253－768.7188879) |
| Azerbaijan | 194.5783817(149.4807611－250.0178206) |
| Bahrain | 45.07373188(34.05387672－57.79758495) |
| Bangladesh | 2322.278652(1587.059224－3465.284806) |
| Barbados | 12.31522264(9.93946009－15.23475142) |
| Belarus | 601.3897039(446.0972878－807.3081441) |
| Belgium | 589.4579606(449.9358469－754.9985188) |
| Belize | 4.373533477(3.662523078－5.117491796) |
| Benin | 33.4238077(22.93573027－46.98961227) |
| Bermuda | 4.120640139(3.312324737－5.126598346) |
| Bhutan | 14.23006475(9.348367833－20.89245619) |
| Bolivia | 361.7919392(257.0277336－488.983693) |
| Bosnia and Herzegovina | 109.4400747(76.83717318－144.6581355) |
| Botswana | 6.217953446(4.078499499－8.756619938) |
| Brazil | 4359.129504(4083.925044－4873.366784) |
| Brunei | 19.99778001(15.78169585－24.8837075) |
| Bulgaria | 240.7543919(184.3573807－308.2730734) |
| Burkina Faso | 64.85375927(43.87005602－86.16763474) |
| Burundi | 102.5268742(71.37623718－141.8768259) |
| Cambodia | 494.7920238(305.4625464－675.7741736) |
| Cameroon | 65.86009113(42.75837124－95.88610988) |
| Canada | 2024.309336(1540.068942－2612.140596) |
| Cape Verde | 4.784929844(3.491452346－6.283654125) |
| Central African Republic | 19.71397565(13.02855691－28.86961646) |
| Chad | 30.46075385(20.37980992－40.25994834) |
| Chile | 732.1574695(556.1852495－946.7010289) |
| China | 39079.13171(32278.92351－47658.34087) |
| Colombia | 1619.074431(1204.604251－2149.565431) |
| Comoros | 9.781708908(6.249933081－14.25269168) |
| Congo | 32.33998779(22.64831072－44.73168873) |
| Cook Islands | 0.576921537(0.435134038－0.745088416) |
| Costa Rica | 184.2957951(137.1538346－243.0476892) |
| Cote d'Ivoire | 71.13826149(47.291346－98.42272578) |
| Croatia | 265.4733897(197.3293733－345.5508036) |
| Cuba | 507.2151335(401.0097946－643.0186687) |
| Cyprus | 55.59277285(45.1908479－67.99031925) |
| Czech Republic | 671.3876401(520.0161434－848.9963252) |
| Democratic Republic of the Congo | 323.3069042(217.5957458－466.041763) |
| Denmark | 242.2260367(184.1370273－313.5431116) |
| Djibouti | 15.17540957(8.844673784－24.59453894) |
| Dominica | 1.622918489(1.252882575－2.044693956) |
| Dominican Republic | 235.187665(151.966287－333.2908466) |
| Ecuador | 738.7946582(550.6885108－987.6492326) |
| Egypt | 2008.068703(1405.914127－2720.231117) |
| El Salvador | 125.1014798(91.33415651－171.5922179) |
| Equatorial Guinea | 6.705675333(4.099442568－10.72489723) |
| Eritrea | 71.37508731(46.58892435－105.0175117) |
| Estonia | 89.087939(67.37656918－116.5225104) |
| Ethiopia | 2502.204898(1792.334711－3370.893632) |
| Federated States of Micronesia | 2.444491316(1.554764135－3.476070272) |
| Fiji | 31.89973776(17.51461007－43.96124383) |
| Finland | 366.9892701(281.1580997－474.705861) |
| France | 3226.028469(2439.626901－4173.914448) |
| Gabon | 14.17102929(10.12533059－19.37311631) |
| Georgia | 122.903252(96.39632495－149.841285) |
| Germany | 5444.021026(4116.955237－7151.557316) |
| Ghana | 69.24165171(51.13096063－90.04402317) |
| Greece | 460.8358455(353.8305826－597.0044251) |
| Greenland | 1.543291481(1.062258698－1.998369192) |
| Grenada | 3.468614394(2.927087466－4.107111025) |
| Guam | 6.008378791(4.016069855－7.51203444) |
| Guatemala | 227.2921797(174.5094456－288.5728255) |
| Guinea | 58.58687879(42.66724946－78.68171958) |
| Guinea-Bissau | 6.112632647(4.008243334－8.274049614) |
| Guyana | 14.50005802(10.81368395－19.26467989) |
| Haiti | 135.6366668(81.62746514－200.2844861) |
| Honduras | 527.8182164(154.4515164－780.0559653) |
| Hungary | 494.4580449(389.170185－617.8056834) |
| Iceland | 38.64783294(32.38555891－45.31904297) |
| India | 23822.80367(19467.44876－28644.48521) |
| Indonesia | 6672.18472(4626.352519－8372.339944) |
| Iran | 3197.953294(1998.741647－3648.907302) |
| Iraq | 1282.324245(894.5700825－1778.654367) |
| Ireland | 231.8958281(171.1340353－306.5973942) |
| Israel | 436.0612423(331.3790001－564.4145159) |
| Italy | 6277.53743(5017.094383－7737.195446) |
| Jamaica | 83.9753129(63.03837897－109.8535394) |
| Japan | 10133.61645(8280.546004－12092.18291) |
| Jordan | 321.274879(249.2267995－417.0924147) |
| Kazakhstan | 580.1527721(480.3405111－701.0750262) |
| Kenya | 238.7575761(172.2769194－338.3643593) |
| Kiribati | 0.516489925(0.383069622－0.741773406) |
| Kuwait | 149.2927152(119.5624671－186.0897799) |
| Kyrgyzstan | 83.18839859(68.1379304－100.5849576) |
| Laos | 139.7995289(86.4726914－196.1624306) |
| Latvia | 113.4763574(86.27773119－146.5740574) |
| Lebanon | 431.296268(305.8656993－582.256865) |
| Lesotho | 15.98668934(9.821050055－23.16232244) |
| Liberia | 14.40159477(9.365057701－20.09772246) |
| Libya | 257.8767633(170.3890775－363.9974315) |
| Lithuania | 149.4895285(113.8486665－190.9877587) |
| Luxembourg | 36.93202114(29.58950078－46.69581338) |
| Macedonia | 72.30435312(52.10774665－96.99764975) |
| Madagascar | 234.3243709(164.2808086－325.3215026) |
| Malawi | 174.8078916(108.0197535－271.4162383) |
| Malaysia | 1378.989124(1024.667701－1813.405917) |
| Maldives | 12.80694172(10.23730704－16.37243181) |
| Mali | 88.78870495(60.26467989－126.5661456) |
| Malta | 26.02902321(21.23512157－31.72728465) |
| Marshall Islands | 1.029611398(0.71726086－1.482485952) |
| Mauritania | 13.45900414(8.80057084－19.04916081) |
| Mauritius | 32.61976674(25.27381323－40.99094529) |
| Mexico | 3533.537902(2967.259908－4174.221712) |
| Moldova | 130.3501428(105.8846377－157.439215) |
| Mongolia | 40.64092081(21.94228532－55.69258028) |
| Montenegro | 37.82978412(30.72944013－46.72167665) |
| Morocco | 1461.999881(1013.030208－2108.983472) |
| Mozambique | 298.9988582(193.1198847－440.3680194) |
| Myanmar | 1436.741952(996.4162669－1901.593956) |
| Namibia | 22.6665068(15.02757697－33.47326065) |
| Nepal | 489.7620128(341.210498－681.3902143) |
| Netherlands | 1000.786361(742.9858635－1295.525146) |
| New Zealand | 157.7977595(122.8458027－195.6491119) |
| Nicaragua | 119.6301199(91.39962222－151.5326591) |
| Niger | 10.46894432(7.113240779－15.031889) |
| Nigeria | 447.1424115(321.7018484－602.6234649) |
| North Korea | 763.4855882(543.666925－1071.902474) |
| Northern Mariana Islands | 2.155577664(1.528209311－2.783005195) |
| Norway | 299.0512366(247.8509391－358.4687861) |
| Oman | 111.5366909(82.32232261－146.1497165) |
| Pakistan | 4884.82454(3650.129859－6521.309739) |
| Palestine | 110.4037387(76.58348695－139.0121893) |
| Panama | 115.6675499(85.33635556－151.9853671) |
| Papua New Guinea | 80.44086493(53.7651238－122.6476972) |
| Paraguay | 168.4044284(117.5781686－228.9464542) |
| Peru | 1044.575473(735.1846278－1430.882843) |
| Philippines | 4014.581918(3101.533922－5168.598924) |
| Poland | 1797.844942(1429.468645－2294.802469) |
| Portugal | 743.5334323(559.4589614－960.6136002) |
| Principality of Monaco | 4.093706298(3.061318443－5.268249561) |
| Puerto Rico | 123.8814105(93.22170972－162.9663791) |
| Qatar | 58.77811393(41.14321859－84.68226828) |
| Republic of Nauru | 0.275173809(0.176933728－0.38241809) |
| Republic of Niue | 0.07306355(0.05061746－0.099136838) |
| Republic of Palau | 1.422402643(1.03093329－1.877117188) |
| Republic of San Marino | 3.236513448(2.345567181－4.444707892) |
| Romania | 832.5028384(653.0081407－1041.48949) |
| Russian Federation | 8773.369628(7283.603698－10521.22879) |
| Rwanda | 157.917359(109.0044564－223.9337472) |
| Saint Kitts and Nevis | 2.511497772(1.7195948－3.275433572) |
| Saint Lucia | 6.416721144(5.240567264－7.777028152) |
| Saint Vincent and the Grenadines | 4.283915697(3.577671284－5.048437392) |
| Samoa | 5.652587312(3.578570841－8.146445282) |
| Sao Tome and Principe | 0.99785217(0.686823139－1.399576911) |
| Saudi Arabia | 2258.993949(1527.766894－3157.331312) |
| Senegal | 46.28244761(30.65467134－61.39041841) |
| Serbia | 344.1502297(260.6025359－453.8761456) |
| Seychelles | 1.590257039(1.327326565－1.899414566) |
| Sierra Leone | 22.77000007(14.02959436－31.32240043) |
| Singapore | 297.5708687(225.5882211－381.1783321) |
| Slovakia | 247.6396778(189.3687744－324.8004502) |
| Slovenia | 102.9713585(76.45926373－136.4707765) |
| Solomon Islands | 14.22600601(7.931387123－22.56642321) |
| Somalia | 133.8007968(86.63590486－193.4366041) |
| South Africa | 467.5437146(405.7458347－582.8403012) |
| South Korea | 5208.567662(3564.475136－6623.38965) |
| South Sudan | 56.96016662(36.42142281－89.98987341) |
| Spain | 2426.220892(1845.609459－3208.036675) |
| Sri Lanka | 886.3275096(622.3334362－1272.831193) |
| Sudan | 569.1902857(353.7689742－853.3572986) |
| Suriname | 11.85905892(9.405757851－14.74121314) |
| Swaziland | 8.485251906(5.290471524－12.20983858) |
| Sweden | 381.6514921(314.0056341－455.942306) |
| Switzerland | 508.593843(381.760292－675.2750479) |
| Syria | 138.243486(89.36176156－190.0570921) |
| Taiwan | 1736.910136(1309.709779－2357.472691) |
| Tajikistan | 27.97181715(21.96878524－35.22939936) |
| Tanzania | 654.2390378(462.7579534－922.8226268) |
| Thailand | 2667.48579(1794.199969－4451.916378) |
| The Bahamas | 11.17488442(8.829491714－14.24544995) |
| The Gambia | 7.788562108(5.601226824－10.37110975) |
| Timor-Leste | 21.33687407(12.98141465－29.48635824) |
| Togo | 26.55208062(16.96990634－37.02713405) |
| Tokelau | 0.038227015(0.025309543－0.053477205) |
| Tonga | 1.564678692(1.123528196－2.162252507) |
| Trinidad and Tobago | 34.87323665(25.21706946－46.53994021) |
| Tunisia | 494.4138771(337.1218927－717.3922545) |
| Turkey | 3270.588286(2493.220006－4322.47722) |
| Turkmenistan | 80.00079319(62.53804181－103.1092984) |
| Tuvalu | 0.27374222(0.18992851－0.379854761) |
| Uganda | 443.462772(291.1414482－627.388923) |
| Ukraine | 2399.988284(1920.462886－2930.293441) |
| United Arab Emirates | 444.2437797(228.9042868－709.6704114) |
| United Kingdom | 2781.58597(2291.332833－3322.896567) |
| United States | 26270.12082(22444.41982－30609.50135) |
| Uruguay | 114.2749382(87.43277858－146.3443384) |
| Uzbekistan | 246.8066242(200.3168903－298.719869) |
| Vanuatu | 3.638743945(2.360866988－5.236494001) |
| Venezuela | 730.7411068(534.4572632－975.2534917) |
| Vietnam | 7788.66868(5008.516682－10487.05544) |
| Virgin Islands, U.S. | 3.197173993(2.52186469－3.926444491) |
| Yemen | 355.5421716(227.6537226－516.5372344) |
| Zambia | 244.0712167(165.6777024－353.5335192) |
| Zimbabwe | 217.7808785(125.4505943－306.9406809) |
